# Comparison of primary analysis strategies of randomized controlled trials with multiple endpoints with application to kidney transplantation

**DOI:** 10.1038/s41598-026-38979-6

**Published:** 2026-02-13

**Authors:** Felix Herkner, Martin Posch, Gregor Bond, Franz König

**Affiliations:** 1 Center for Medical Data Science, Medical University of Vienna, Vienna, Austria; 2 Division of Nephrology and Dialysis, Department of Medicine III, Medical University of Vienna, Vienna, Austria

**Keywords:** Clinical trial simulation, Kidney transplantation, Multiple endpoints, Generalized pairwise comparisons, Composite endpoints, Computational biology and bioinformatics, Medical research

## Abstract

Relying on a single primary endpoint in randomized controlled trials (RCTs) is often infeasible, for example due to rare or heterogeneous events. Regulatory guidance therefore allows multiple endpoints, but different analytical strategies address different scientific questions and null hypotheses, even when applied to the same set of variables. We explored three approaches to consider multiple endpoints in the primary analysis of RCTs, as stated in the FDA and EMA guidelines on multiplicity: (i) a composite endpoint (CE), (ii) multiple testing and multiplicity correction (MTMC), and (iii) a hierarchical non-parametric procedure, called generalized pairwise comparisons (GPC). Using clinical trial simulations, we compared these strategies’ power in two-arm RCTs perform when testing strategy-specific hypotheses across a range of scenarios reflecting endpoint prioritization, correlation between endpoints, and opposing treatment effects. When testing time-to-event endpoints, global testing strategies (CE and GPC) generally achieved higher power than MTMC. However, we also demonstrate that global procedures may yield statistically significant results even when treatment effects are heterogeneous across endpoints, underscoring the importance of careful interpretation and component-wise assessment. As trials increasingly use multiple endpoints, understanding the trade-off between statistical efficiency and interpretability, and provide practical guidance for choosing endpoint definitions and primary analysis strategies in future trials.

## Introduction

Randomized controlled clinical trials (RCTs) are prospective studies randomly allocating participants to two or more groups to compare novel treatments to a control group. In RCTs it may be difficult to identify a single endpoint that fully reflects treatment effects while being accessible to efficient statistical analysis and clear interpretation. Reasons include event rates too low to provide sufficient power^1^ and the inability of a single outcome to capture complex treatment effects. A potential remedy is to include several endpoints of interest in the primary analysis^2^.

Several strategies have been proposed for handling of information from different endpoints in confirmatory trials^2,3^, where control of the type-I-error rate—the probability of making a false discovery—is a key requirement^4^. One strategy defines a primary endpoint and treats all the others as secondary endpoints^3^ but choosing a suitable hierarchy is difficult—the most important endpoints in clinical endpoints may require infeasible sample sizes, while others are only relevant if treatment is at least non-inferior for more critical outcomes. Combining the endpoints of interest into a composite endpoint is another strategy discussed in U.S. Food and Drug Administration (FDA) and European Medicines Agency (EMA) guidance documents on multiplicity issues^3,5^. Well established composites exist in cardiovascular (e.g., *major adverse cardiovascular events*, MACE) and oncology (e.g., progression-free survival) trials^1,6,7^. EMA guidelines for clinical studies of immunosuppression after solid organ transplantation^8^ state as primary efficacy endpoint a composite of overall survival, graft failure, rejection and graft function. In kidney transplant trials composite endpoints commonly used include death and graft loss combined with, e.g., rejection^9^, or graft function^10^ but the composition varies^11^. Composite endpoints allow a single hypothesis test and no further adjustments for multiplicity are needed^3^. A second approach is to test each endpoint separately while maintaining the family-wise-error rate (FWER) - the probability of rejecting at least one true null hypothesis in a series of tests, e.g., several tests conducted within one study - by adjusting the test-wise alpha-level or p-values. Lastly, treatment effect estimates based on non-parametric hierarchical comparisons, like the win ratio and net treatment benefit^12,13^, have become popular in cardiovascular trials in the recent past and were also proposed for kidney transplant studies^14^ as alternatives to composite endpoints.

Our focus is on trials investigating personalized immunosuppression after kidney transplantation. Immunosuppressants are essential to reduce the risk of organ rejection after kidney transplantation but have limiting side effects like an increased risk of infections, malignancies and cardiovascular as well as metabolic changes^15^. Thus, a balance between insufficient and excessive immunosuppression must be pursued and any one variable alone hardly reflects this balance. Furthermore, due to advances in post-transplant care, events like graft loss or death occur infrequently^16,17^ and although clinically important would result in underpowered analyses. For example, even a reduction of mortality from 5% in the standard of care arm to 2.5% in the treatment arm would require a sample size of 900 participants per group for a typical $\chi ^2$-test to have a power of 80%. These numbers are infeasible for most trials in kidney transplantation. More frequently occurring events like rejections or infections are of less clinical relevance and only reflect one kind of treatment failure (e.g., excessive immunosuppression). For such endpoints it is also not obvious how to include participants who died or lost their graft before experiencing other events of interest in the analysis due to unequal observation times.

As the calculation of sample size and power is not straightforward when including multiple endpoints and possibly correlations among them^18^, it remains unclear which approach is most powerful in different RCT settings. When analytical solutions for the computation of operating characteristics, like statistical power, are difficult to find, simulation studies provide a means of estimation^19–22^. Clinical trial simulations generate virtual patient under specified assumptions mimicking the outcomes of a proposed clinical trial. By creating tens of thousands of repetitions of such a study, a precise estimate of how different approaches perform in the investigated setting can be obtained. In this study, we use simulations to investigate the power of different endpoint definitions and associated statistical analysis. We explore a wide range of assumptions regarding treatment effects and event rates particularly relevant but not restricted to kidney transplant trials.

First, the motivating trial and clinical setting is set out in section Motivating example. This is followed by briefly introducing the mentioned strategies to include multiple endpoints together with statistical methods used for hypothesis testing (section Statistical methods). Section Setup of the simulation study describes the simulation structure including data generating mechanisms and operating characteristics. A detailed analysis of two simulated examples is conducted in section Motivating example revisited to explain how the motivating trial is considered in the simulation and highlight important results. Results of the simulation study are reported in section Results. The findings are discussed in section Discussion.

## Motivating example

To provide the context of this work, the motivating trial is briefly introduced. The *TTVguideIT*^23,24^ trial is a randomized, controlled, two-arm, multicentre, clinical phase II trial. It compares immunosuppression after kidney transplantation guided by a novel biomarker versus standard of care. Candidates for the primary endpoint are overall survival, graft loss, rejection and infections. All of them constitute important endpoints in immunosuppression showing failure or major side effects of the treatment. A treatment significantly improving overall survival constitutes an important clinical benefit but death as an endpoint bears the statistical problem of low event rates. Graft loss and rejection are clinically severe but also rather rare events^17^. Infections occur frequently but using a reduction in the number of infections alone for trial decision—while constituting a significant treatment benefit for patients—might favour treatment schemes resulting in inadequate immunosuppression even if this means higher rates of graft loss or death. For examples of how these endpoints can be defined in kidney transplant trials, as well as advantages and disadvantages in clinical and statistical terms see Table 1. For more examples of possible definitions of endpoints, we refer to systematic reviews of outcomes in kidney transplantation^11,25^.

Note that while the primary analysis of the TTVguideIT trial tests a non-inferiority hypothesis followed by testing for superiority in a hierarchical testing procedure^24^, here we focus on one-sided testing (testing superiority) and additionally consider two-sided testing, i.e. whether there is a difference between two treatment groups or not.

**Table 1:** Overview of potential endpoints of interest in transplant trials investigating immunosuppression, their advantages and shortcomings.

Endpoint	Endpoint definition^a^	Advantages	Disadvantages
Overall survival	Death	“Hard”, unambiguous endpoint of high clinical relevance. Improvement of overall survival provides convincing evidence of treatment benefit.	May lack power because of low event rate
Graft loss	Graft failure and restart of dialysis	”Hard” and rather unambiguous endpoint of high clinical relevance	May lack power because of low event rate. Clinically less significant than death. Reflects mostly insufficient immunosuppression
Rejection	Rejection found by biopsy (according to current version of the Banff classification^26^)	Important clinical endpoint. Widely accepted standards for event definition exist	Definition depends on the classification. Low event rate associated with lack of power. Reflects mostly insufficient immunosuppression
Infections	Diagnosis based on the Infectious Diseases Guidelines 2019^27^ requiring either inpatient treatment, use or escalation of antimicrobial treatment or reduction of immunosuppression	Higher event rate. Constitutes the main side effect of excessive immunosuppression	Endpoint definition is not unambiguous and no well-established standard exists. High risk of bias if assessor is unblinded. Infections may be considered clinically less severe than aforementioned endpoints

^a^ Based on definitions used in the primary endpoint of the TTVguideIT trial^23^.

## Methods

### Statistical methods

Our setting is a two-arm RCT with 1:1 allocation ratio. Throughout this section we use the terms treatment arm (or arm T) to refer to the group receiving the novel treatment and name the other arm control group (or arm C). It is assumed that all individuals start at time zero (i.e., time of randomization) and are all observed for the same fixed time *s*. Consider a number of *k* endpoints, $E_1,..., E_k$, which are candidates for the primary endpoint analysis.

**Fig. 1:**
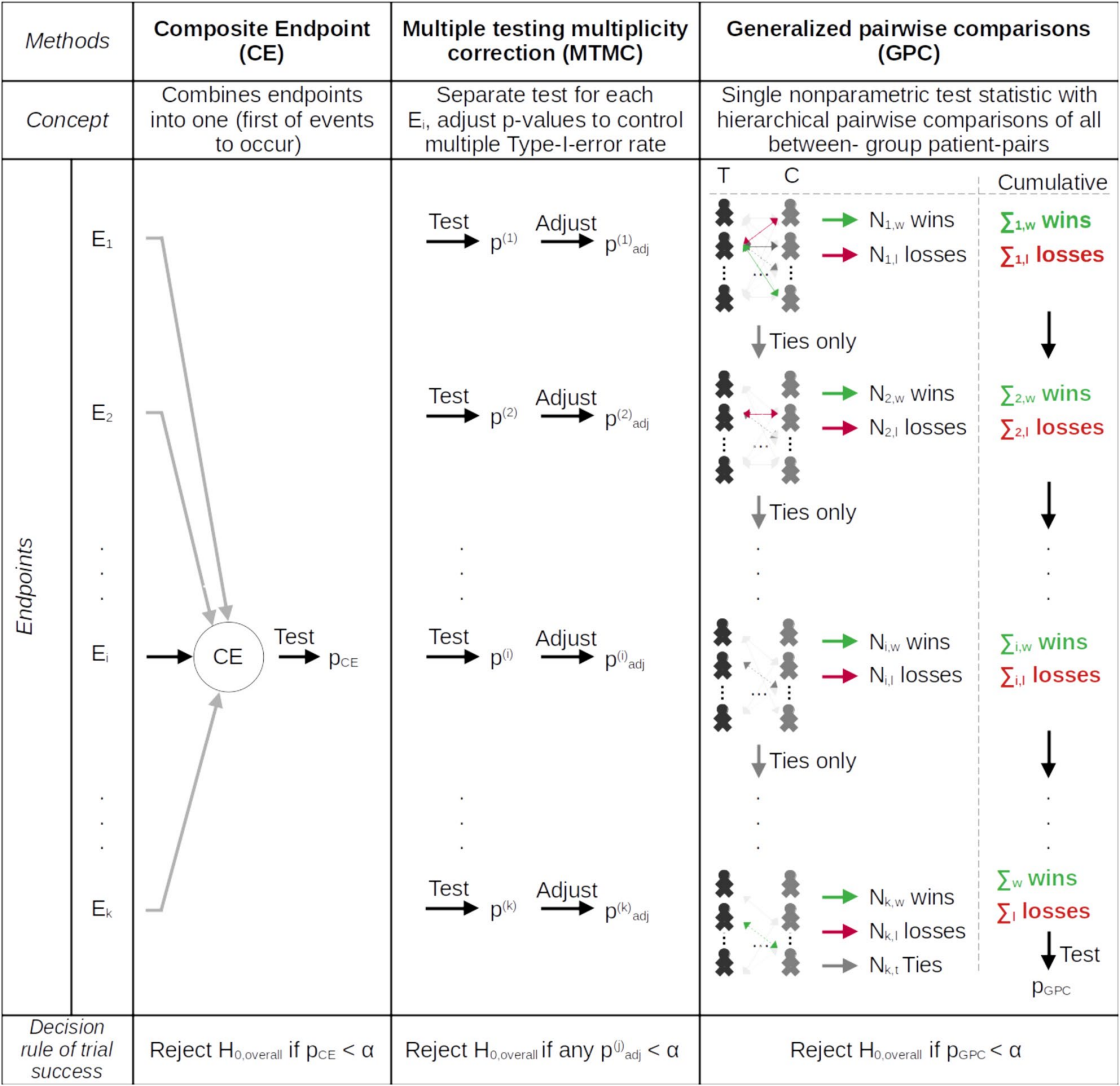
Overview of investigated options to include multiple endpoints in the primary analysis. $E_1, \dots , E_k$ are the *k* endpoints of interest. They are ordered by their assigned clinical importance ($E_1$ being the endpoint with highest priority). Let $E_i$ be the endpoint to first occur in an exemplary participant and therefore defining the composite endpoint (CE) for this individual. $p^{(i)}$ is the p-value of a test performed on the *i*th endpoint, or the one test of the composite endpoint ($p_{CE}$) or the GPC ($p_{GPC}$), respectively. $\alpha$ denotes the overall nominal significance level for the primary analysis of the trial. $p^{(i)}_{adj}$ is the p-value for a test on endpoint *i* adjusted by multiplicity correction. T and C in the last column mean treatment and control arm, respectively. Note that for each endpoint the pairwise comparisons of the second participant in the treatment group with every participant in the control group is depicted, the other comparisons being greyed out. The number of comparisons decreases as only tied or uninformative pairs are evaluated in lower prioritized endpoints. $N_{i,j}$, $j \in \{w, l, t\}$ is the number of wins (w, individual of the treatment arm has the better outcome) or losses (l, individual of the control arm has a better outcome) or ties (t, no winner can be declared) for comparison in terms of endpoint *i*. $\sum _{i,j}$ are the cumulative numbers of wins, losses and ties summing over comparisons on endpoints 1 to *i*, in the pre-defined hierarchical order. $\sum _{j}$ are the total numbers of wins, losses and ties, i.e., the cumulative sums from 1 to *k*. ”Test” means testing using any appropriate hypothesis test, e.g., log-rank tests for time-to-event endpoints. The test of GPC test statistics can be based on approximate results for U-statistics, see paragraph ”Generalized pairwise comparisons”.

Three main strategies that make use of multiple endpoints are options for analysis in line with regulatory guidance on multiplicity by FDA^5^ and EMA^3^. This includes (i) combining the individual endpoints into a single composite endpoint (CE), (ii) performing statistical tests on each individual endpoint itself and correcting for multiple testing (in the following multiple testing and multiplicity correction, MTMC), and (iii) a non-parametric hierarchical evaluation procedure, the Generalized Pairwise Comparisons (GPC). An overview of the strategies and the basic idea of the procedures is given in Figure 1.

#### Composite endpoint (CE)

The composite endpoint combines the endpoints $E_1,..., E_k$ into one single outcome. In the case of time-to-event data we define the composite endpoint as the time to the first of the events that a participant experiences. Differences in outcomes between treatment and control group can then be tested with standard methods of survival analysis. Here we consider the log-rank test. Let $h_T(t)$ and $h_C(t)$ be the hazards of experiencing any of the events at time *t* in arm T and C, respectively, and assume that they are proportional throughout the observation time, i.e. $\frac{h_T(t)}{h_C(t)} = c$ for all times *t*, and *c* being a constant. The null hypothesis for one-sided tests - i.e., testing for superiority of the treatment - is $H_0: h_T(t) \ge h_C(t)$ for all *t* versus $H_1: h_T(t) < h_C(t)$ for some *t*. Respective hypotheses for two-sided tests - i.e., testing for differences between groups - is no difference in hazards between groups, i.e, $H_0: h_T(t) = h_C(t)$ for all *t* versus $H_1: h_T(t) \ne h_C(t)$.

Another option is to define a binary composite endpoint commonly defined as an individual reaching the endpoint if at least one of the events occurs within follow-up^18^. This may be helpful for interpretation, as the timing of events is sometimes not equally relevant in clinical practice as the occurrence of any endpoint. If the follow-up time is the same for all individuals, differences between groups can be tested using a $\chi ^2$-test. Denote $p_T$ and $p_C$ the proportion of individuals reaching the composite binary endpoint by the end of their follow-up in arm T and C, respectively. The null hypothesis for one-sided tests is $H_0: p_T \ge p_C$ versus $H_1: p_T <p_C$. Accordingly, for two-sided tests the null hypothesis is $H_0: p_T = p_C$, i.e., proportions are equal across arms. The alternative hypothesis then is $H_1: p_T \ne p_C$.

To provide a meaningful interpretation of the composite endpoint, there are certain requirements. The treatment should affect all components of the composite endpoint alike but at least in the same direction (beneficial effect on all endpoints) and the components should all be ideally of equal clinical importance^3,5^.

#### Multiple testing and multiplicity correction (MTMC)

A second approach involves testing the endpoints $E_1,...,E_k$ separately and subsequently correcting either p-values or the test-wise significance level to control the FWER. Endpoints can be tested, e.g., by means of log-rank tests, as described above. Some endpoints might be subject to competing risks, such as death, i.e. death can prevent observation of these endpoints. Options include testing differences in cause-specific hazards $h_C$ and $h_T$, respectively, using log-rank tests, and targeting cumulative incidence functions (CIF), $CIF_{C}$ and $CIF_T$, using Gray’s test^28^. As corrections for multiplicity we consider the classical Bonferroni correction, a variant of it using pre-defined weights, and Hommel’s correction.

The *Bonferroni correction* modifies the decision criteria applied to each endpoint. The p-value of each single test is adjusted to be the individual test’s p-value multiplied by the number of tests performed (i.e., the number of endpoints, *k*): $p_{adj}^{(i)} = min(p^{(i)}*k, 1)$. We reject the overall null hypothesis of no treatment effect, if at least one of the tests rejects the null hypothesis after adjusting the p-value, i.e., any of the p-values satisfies $p_{adj}^{(i)} < \alpha$.

A *weighted Bonferroni procedure*^5^ can be applied by adjusting the p-value for each test separately according to pre-defined weights. If we have a sufficiently precise estimate of incidences and treatment effects of endpoints beforehand, pre-defining weights to tailor the test to certain scenarios might increase power. Let $\omega _i > 0$ be a weight for the endpoint *i*, $i = 1,..., k$ and $\sum _i \omega _i = 1$. Then the adjusted p-value $p^{(i)}_{adj}$ of endpoint *i* is $p^{(i)}_{adj} =min(\frac{p^{(i)}}{\omega _i}, 1)$.

Simes^29^ proposed a global test that is more powerful than the Bonferroni correction. In order to interpret the tests on individual endpoints, *Hommel’s correction*^30^ is used, a closed test extension of Simes’ method. FWER is controlled for independent tests, although it has been shown to hold under broader assumptions^2,31^.

We also provide the results using one single endpoint for decision. Testing an individual endpoint at the full nominal significance level $\alpha$ corresponds to the power of a hierarchical (fixed-sequence) testing procedure in which this endpoint is placed first in the testing hierarchy. In hierarchical testing, hypotheses are tested sequentially in a pre-specified order, and testing proceeds to subsequent endpoints only if all preceding hypotheses are rejected, thereby preserving the overall type I error rate without multiplicity adjustment.

#### Generalized pairwise comparisons (GPC)

GPC are a class of non-parametric tests that can accommodate hierarchical evaluation of several endpoints, possibly of different variable types such as binary, continuous, and time-to-event data^12,32^. Common estimators of treatment effect are, e.g., the *net treatment benefit (NTB)*^12^ or the *win ratio*^13^. Estimation of both metrics involve the same steps, as outlined in Figure 1 for each endpoint $E_i$. First, all possible pairs consisting of individuals from different groups are formed. Each pair is then compared on the basis of the observed outcomes of the involved individuals. Depending on the variable type, adequate decision rules must be formulated to either declare the pair favourable (or ”winner”) if the outcome in the treatment group is better. Pairs are marked unfavourable (”loser”) if the control group is better. Pairs can also be a ”tie” (neutral) or uninformative, if the outcomes do not allow for deciding on a more favourable outcome or because of missing information. NTB or win ratio are then calculated as the difference between favourable and unfavourable pairs divided by all pairs and number of wins divided by number of losses, respectively. While labelling of pairs is straightforward for some variable types like binary outcomes, time-to-event data in presence of censoring needs more consideration. A scoring rule proposed by Buyse^12^, also referred to as *Gehan’s scoring rule*, categorises pairs as uninformative if censoring does not permit a clear decision. Among other shortcomings, this reduces power^33,34^. Alternative scoring rules exist. Péron and colleagues^33^ make use of the Kaplan-Meier estimator of the survival function to resolve pairs with censored observations.

One sided tests test the hypothesis $H_0: \text {NTB} \le 0$ versus $H_1: \text {NTB} > 0$. The null hypothesis for a two-sided test is that NTB is zero and the alternative hypothesis states that NTB is different from zero. Classical GPC testing uses a randomization test by randomly shuffling the treatment allocation^12^. Alternatively, extensions of U-statistics’ asymptotic properties to right-censored data exist^35^. Applying this asymptotic inference in combination with Gehan’s scoring rule is extremely favourable in terms of computation time and thus these are the options used in our simulation.

In the GPC framework, several endpoints can be evaluated in a single analysis in a pre-defined hierarchical manner. The individual comparison of a patient in the treatment group with a patient in the control groups starts with the endpoint that is assigned the highest priority and pairs are classified as favourable or unfavourable based on the participants outcome of this endpoint. Only if no clear decision can be taken comparing the pair of observed values on the first endpoint, the evaluation moves on to the second endpoint in the hierarchy. Thus, ties or uninformative pairs are compared by the second endpoint in the hierarchy and so on (see also Figure 1). The total number of wins and losses is then summed and treatment estimates as well as hypothesis tests can be constructed just as described above for one endpoint. It is common practice to order endpoints according to clinical relevance^36^.

### Setup of the simulation study

Clinical trial simulations are used to evaluate performance (or *operating characteristics*) of a trial design^37^, analysis approach^38^ or choice of endpoint definitions^19,21,22^. We focus on evaluation of statistical power, defined as the probability of rejecting the global null hypothesis when there is a treatment effect on at least one of the endpoints, of different ways to incorporate several endpoints into the primary analysis of a clinical trial.

Assuming data-generating mechanisms, we can imitate trials by drawing patient-level (pseudo-)random outcomes. This enables evaluation under known conditions. A scenario refers to a unique combination of study parameters, like sample size, treatment effects, and event rates. Each scenario was simulated 10,000 times and the result (in our case whether the used approach leads us to declare trial success or failure) in each simulated study is recorded. This is akin to reproducing a trial with precisely the same study design sampling from the very same population. Performance measures (like the proportion of declared trial successes) over all repetitions can be used as an estimate of the operating characteristic (e.g. statistical power).

Important parameters of the simulation study are listed in the Supplementary Tables S2-S4, together with the parameter values studied. The simulation parameters were selected to reflect expected real-world conditions, as well as more extreme yet plausible scenarios inspired by kidney trials. Parameters are hazard ratios from 0.5 to 1.5, proportions of endpoints within follow-up of 5% and 15% of the first two endpoints - which are also considered the clinically more important ones - as well as 20% and 35% for the third clinically less important endpoint. Group sizes of 130, 250, 500 and 1000 were investigated. These scenarios were investigated in three different setups. One setup included a terminal event, death, and endpoints were uncorrelated. Another setup assumed no relation between endpoints; no terminal event is present and endpoints are uncorrelated. A third setup used correlated endpoints when none of the endpoints is terminal. Spearman rank correlations between the first and the second and the first and the third endpoint were set to values from 0 to 1.

#### Data generating mechanism

**Fig. 2:**
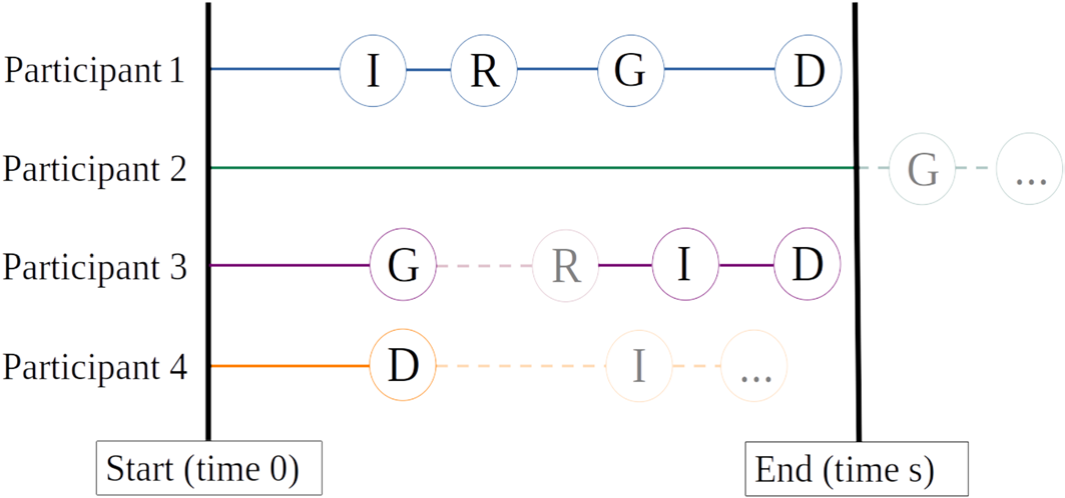
Exemplary individual participant courses. All participants are followed up for the same time *s*. Thus, the individual follow-up period starts at time 0 and ends at time *s*. Participant 1 has an infection (I) first, a rejection (R) is detected, then loses the kidney graft (G) and eventually dies (D) before end of follow-up. The time-to-event composite endpoint for this participant is the time to infection. The first event for participant 2 (G) occurs after the study is finished for this participant, i.e., participant 2 experiences no event within follow-up and is administratively censored for every endpoint. Participant 3 losses their graft whereupon rejections cannot occur any more. Infections and death can still be observed. Participant 4 dies prior to any other event which can therefore not be observed.

Three data generating mechanisms were considered: (a) uncorrelated endpoints but one of the endpoints (death) prohibits observation of other events occurring later, (b) endpoints where no such terminal endpoint exists but correlations among the endpoints are present, and (c) completely unrelated endpoints without terminal events nor correlations. In the first mechanism, event times of each endpoint for each participant are synthetic data drawn from three independent exponentially distributed variables (one for each endpoint) for each treatment arm separately. The composite time-to-event endpoint is the time to the first of the events to occur if it is observed within the follow-up time *s*, where in the simulation study the individual follow-up time is set to $s = 10$. Otherwise patients are censored at time *s* (administrative censoring, see Participant 2 in Fig. 2). A binary composite endpoint is defined as having any event within follow-up. Single endpoints are defined as time to the respective event or end of follow-up, whichever happens first. Note that death prevents us from observing an infection or graft loss event and no rejections can occur after loss of the graft (see Participants 3 and 4 in Fig. 2). Therefore, these endpoint times are the minimum of the event of interest, death or end of follow-up. The three approaches described in Section Statistical methods take these so-called *intercurrent event* into account in differing ways (see also Section Discussion and Table 3).

The second data generating mechanism (b) makes use of copulas to simulate endpoints with certain correlation structures^39^. Conceptually, the endpoints are generated jointly as a multivariate normal distribution where the correlation is set in the variance-covariance matrix directly. This distribution is transformed to a copula, a multidimensional distribution with uniform marginals. The marginal distributions can then again be transformed to an exponential distribution with suitable parameter by applying the exponential quantile function. The resulting exponentially distributed variables can be treated like the latent variables we get with the first data generating mechanism but will be correlated according to the variance-covariance structure we imposed. Again, administrative censoring is performed at the end of the interval for single and composite endpoints as described above. This attenuates the observed correlations (see Supplement Figure S9). There is no further modification of the latent endpoint variables, as opposed to the semi-competing risk structure of the first data generating mechanism. The last data generating mechanism (c) corresponds to leaving independent latent variables as they are, and only applying administrative censoring. In the remainder of the paper endpoints are named death, graft loss and infections if there is a terminal event, death, that makes observation of the other endpoints impossible. We name endpoints more generally $E_1$, $E_2$ and $E_3$ if there is no terminal event. See Supplementary Section A, online, for detailed definitions and on how simulation parameters are used to calculate parameters of the distributions.

Note that we include rejections only into the simulated case studies in Section Motivating example revisited and only consider three endpoints, death, graft loss and infections - or $E_1$, $E_2$ and $E_3$, respectively - in the simulation study for the sake of clear illustration. Highest clinical priority in the GPC hierarchy is therefore assigned to death (corresponding to endpoint $E_1$), followed by graft loss ($E_2$) and least priority to infections ($E_3$).

## Motivating example revisited: examples of two simulated studies

To convey the basic ideas of how simulation studies are of use for our purpose, we introduce two exemplary studies. In this way also important results found in the systematic simulations described in detail in Section Results can be demonstrated. In the following, two scenarios are analysed in detail, where the setting is an RCT including 260 participants allocated 1:1 (130 per group) to standard of care or a novel treatment. One-sided tests for superiority of the treatment are used. We deliberately choose one study (i.e, a single simulation repetition) per scenario that exhibits certain properties for demonstration. This is in contrast to gathering insights into the operating characteristics of the statistical approaches in a scenario, where performance measures over many thousands of repetitions must be used, as described in section Setup of the simulation study.

**Table 2:** Overview of the simulated outcomes and statistical analyses of two simulated exemplary studies.

	Simulated case study 1			Simulated case study 2		
	Incidence: D, G, R low; I high Treatment effect: G, R, I strong; D none			Incidence: G, R low; D, I high Treatment effect: I strong; G moderate; D, R none		
	Control	Treatment	p-value$^{c}$	Control	Treatment	p-value$^\textrm{c}$
Death *n (%)*	4 (3.1%)	3 (2.3%)	0.3445	22 (16.9%)	23 (17.7%)	0.6054
Graftloss *n (%)*	5 (3.8%)	0 (0%)	**0.0117**	5 (3.8%)	4 (3.1%)	0.3843
Rejection *n (%)*	5 (3.8%)	2 (1.5%)	0.1169	6 (4.6%)	6 (4.6%)	0.136
Infection *n (%)*	45 (34.6%)	32 (24.6%)	0.0352	48 (36.9%)	27 (20.8%)	**0.0014**
**Endpoints considered: D, G, R**						
Composite *n (%, RMST)*$^{a}$	13 (10%, 8.56)	5 (3.8%, 8.88)	**0.0244**	32 (24.6%, 8.07)	31 (23.8%, 7.83)	0.4982
MTMC	–	–	0.0351	–	–	1
GPC	e% / f% / u%$^\textrm{b}$		**0.0247**	e% / f% / u%$^\textrm{b}$		0.5376
Death	100% / 3.1% / 2.2%			100% / 14.9% / 16.7%		
Graft loss	94.7% / 3.8% / 0%			68.4% / 3.1% / 2.5%		
Rejection	90.9% / 3% / 1.4%			62.8% / 3% / 2.4%		
Ties left	86.54%			57.41%		
**Endpoints considered: D, G, I**						
Composite *n (%, RMST)*$^\textrm{a}$	52 (40%, 6.99)	35 (26.9%, 7.75)	**0.0108**	69 (53.1%, 6.24)	50 (38.5%, 7.21)	**0.0057**
MTMC	-	-	0.0351	-	-	**0.0042**
GPC	e% / f% / u%$^\textrm{b}$		**0.0083**	e% / f% / u%$^\textrm{b}$		0.0282
Death	100% / 3.1% / 2.2%			100% / 14.9% / 16.7%		
Graft loss	94.7% / 3.8% / 0%			68.4% / 3.1% / 2.5%		
Infection	90.9% / 28.4% / 18.6%			62.8% / 23.8% / 10.1%		
Ties left	43.85%			28.88%		
**Endpoints considered: D, G, R, I**						
Composite *n (%, RMST)*$^{a}$	55 (42.3%, 6.89)	37 (28.5%, 7.68)	**0.0081**	71 (54.6%, 6.14)	53 (40.8%, 7.02)	**0.0094**
MTMC	–	–	0.0468	–	–	**0.0056**
GPC$^{b}$	e% / f% / u% $^\textrm{b}$		**0.0056**	e% / f% / u%$^\textrm{b}$		0.0403
Death	100% / 3.1% / 2.2%			100% / 14.9% / 16.7%		
Graft loss	94.7% / 3.8% / 0%			68.4% / 3.1% / 2.5%		
Rejection	90.9% / 3% / 1.4%			62.8% / 3% / 2.4%		
Infection	86.5% / 27.3% / 18%			57.4% / 21.4% / 9.2%		
Ties left	41.27%			26.88%		

$^\textrm{a}$ RMST = restricted mean event-free survival time, restricted to 9 time units.

$^\textrm{b}$ GPC pairs of the respective endpoint: e%: pairs evaluated / f%: favourable pairs / u%: unfavourable pairs. Percentages are fractions of all pairs.

$^\textrm{c}$ The first four one-sided p-values are calculated from log-rank tests of the individual endpoints. The composite time-to-event endpoint is tested using a log-rank test (one-sided). For MTMC, the smallest Bonferroni-adjusted one-sided p-value is reported. The overall one-sided p-value of the GPC is reported. Significant p-values (< 0.025) are bold. *D* death, *G* graft loss, *R* rejections, *I* infections, *MTMC* multiple testing and multiplicity correction, *GPC* generalized pairwise comparisons.

### Simulated case study 1

(Low incidence of death, graft loss, and rejections, high incidence of infections. Strong effect on graft loss, rejections, and infections, no effect on death. Details on the parameters are given in Supplementary Section B) Using these parameter settings, one study is simulated and chosen for demonstration. In the left column of Table 2, the observed events of each individual endpoint and the composite endpoint definitions are summarized. Three combinations of the four endpoints introduced are investigated and all three of the strategies introduced in Section Statistical methods are applied for each combination. The cumulative incidence function (CIF) plots of single and composite endpoints are visualized in Supplementary Fig. S1 and S2. Endpoints commonly used together in kidney transplantation include death, graft loss and rejections. Other combinations also include infections either additionally to death and graft loss or incorporate all four endpoints.

First, the log-rank test is used to test differences between the hazards of reaching the respective composite endpoint in the two groups (”Composite” rows in Table 2), which rejects on a one-sided nominal significance level of 2.5% for all endpoint compositions in the study chosen in scenario 1.

Looking at the results of the GPC method (”GPC” rows in Table 2), for each endpoint the respective percentages of pairs evaluated and classified (as favourable or unfavourable) out of all pairs are given. For example, when considering D and G, all pairs are first compared by time until death where 3.1% are marked as favourable, 2.2% as unfavourable, leaving 94.7% uninformative pairs to be compared by time until graft loss. The total number of wins and losses of all comparisons is used to test the null hypothesis. The GPC also rejects the overall null hypothesis regardless of the endpoint combination used in the first example (”GPC” rows in Table 2).

Finally, the MTMC fails to reject the null hypothesis after correction for multiplicity regardless of which of the three combination of endpoints are used. If this situation is anticipated, weights could be assigned beforehand to unequally adjust the p-values. For example, if the treatment is expected to primarily affect graft survival, defining the weight for a test of the graft loss endpoint $\omega _G = 0.5$ leads to a test-specific p-value of $p_{adj}^G = 0.0117/0.5 = 0.0234$ and would allow us to just reject the overall null hypothesis in this case. Here, the need to pre-specify such weights becomes obvious. The main advantage of the MTMC is to be able to directly see based on which endpoint(s) the decision of trial success is made.

#### Simulated case study 2

(High incidence of death and infections, low incidence of graft loss and rejections. Strong treatment effects on infections, and graft loss, no effect on death and rejections. Details on the parameters are given in Supplementary Section B) While the composite endpoint strategy in this example also leads us to declare trial success when including infections in the analysis, it fails when only looking at D, G and R (see right column of Table 2). This is as expected as treatment is only affecting G and the event rate of graft losses is low. The same is true for the MTMC approach using Bonferroni correction. As in the first example, there is very little impact of considering R in the analysis in addition to D, G, I.

The GPC method fails to reject the overall null hypothesis in all endpoint compositions in the second example. Looking at the pairs solved in each stage, it can be seen why this is the case. In contrast to the 5.3% of pairs being decided by the first endpoint in the hierarchy, D, in the first example study, in the second it is over 31%. As there is no difference in true hazards of D, these pairs are ”lost” for comparison by endpoints that might indeed enable the GPC to detect a difference.

## Results

This section presents results from an extensive simulation study comparing composite endpoints, multiplicity-adjusted multiple testing, and generalized pairwise comparisons across clinically motivated scenarios. Overall, the results show consistent and interpretable patterns, with differences in power primarily driven by endpoint prioritization and testing strategy, correlation between endpoints, and treatment effect heterogeneity. Results are first shown for uniformly beneficial treatment effects and the presence of a terminal event, death, followed by analyses examining opposing treatment effects and correlation, with additional scenarios provided in the Supplementary Sections C-E.

For clarity of presentation, the simulation results shown focus on three endpoints: death, graft loss, and infections. Including an additional low-incidence endpoint such as rejection was found to alter the results only in subtle and predictable ways, as also illustrated in Section Motivating example revisited.

The graphs presented in this section constitute a small fraction of all scenarios investigated and are chosen to illustrate general patterns and notable exceptions. As only a limited number of dimensions can be displayed in a single graph, some parameters were held fixed in the background and are not explicitly shown; these are listed in the figure titles and captions and apply to all scenarios shown.

### Time-to-event endpoints

**Fig. 3:**
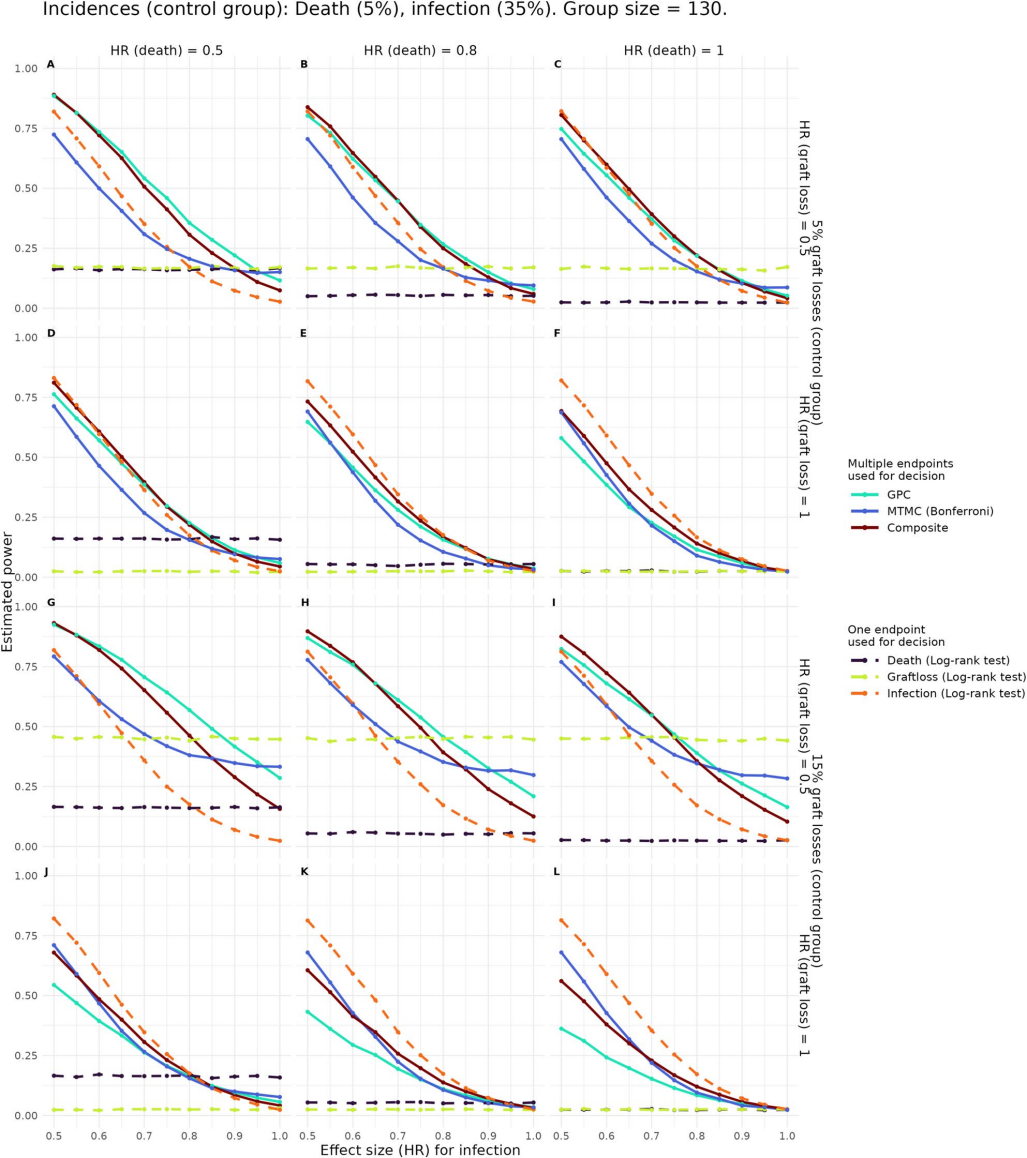
Estimated power of the approaches using time-to-event endpoint definitions one-sided tests in key scenarios. In the first row of plots, the expected proportion of graft losses in the control group amounts to 5% and a marked treatment effect on graft loss (hazard ratio (HR) = 0.5) is present. In the second row of plots, the expected proportion of graft losses is 15% and there is no treatment effect on graft loss (HR = 1). Parameters fixed in all of the shown scenarios are the expected proportions of deaths and infections in the control group (5% and 35%, respectively). The x-axis of plots depicts the HR of infections (0.5, large difference, to 1, i.e., equal hazards for infection in both groups). On the y-axis the estimated power is plotted. The HR of death increases within a row from left to right taking values 0.5, 0.8, and 1. Solid lines identify procedures taking all endpoints into account; Bonferroni correction (blue), Composite endpoint (darkred), GPC (turquoise). Dashed lines indicate that tests are performed on one single endpoint without multiplicity correction; one-sided log-rank tests of graft loss (green), infections (orange), and death (black).

First, we look at our base scenarios: Independent time-to-event endpoints were simulated and death prevents us from observing further events (Fig. 3). As discussed in the methods section, the composite endpoint is thus defined as time to the first of the events to occur. Secondly, for the alternative analysis strategy performing a statistical test for each single endpoint separately, log-rank tests are used for each individual time-to-event endpoint D, G, and I, and corrected for multiplicity. Finally, the GPC compares all pairs of patients between the two groups based on their survival time for endpoints in the hierarchy D, G and I.

In most scenarios, GPC and the composite endpoint outperform MTMC in terms of power (see top and third row in Fig. 3). The GPC method is even slightly more powerful than the composite in many scenarios where strong treatment effects on all endpoints exist (e.g. Fig. 3A and G as well as Figure 3B and H for HR (infection) > 0.75). However, the power of the GPC method depends heavily on the prioritized endpoints, especially death, even if the incidence of those endpoints is low (see decline in power from left to right within a row in Figure 3). This is more marked if one of those endpoints occurs frequently (as seen in the lower rows of plots in Figure 3). On the other hand, the composite endpoint approach relies on the endpoints most frequently occurring (here infections and in the lower two rows of Figure 3 also graft loss) and retains more of its power than GPC even if there are no treatment effects on one of the lower-incidence endpoints death or graft loss (e.g. Figure 3A versus C). The MTMC procedure’s power also changes mostly with the effect size of infections but is far less altered by one of the endpoints death or graft loss not having any treatment effect, even if this endpoint is more frequently reached. However, it only exceeds the power of testing infections alone if there is nearly no treatment effect on infections, but still some on graft loss or death (towards the right hand side of all plots in Figure 3). When looking at larger group sizes, the power of individual tests of graft loss and death increases even if the event rates are low. Therefore, also the number of scenarios in which the MTMC’s power exceeds all other approaches increases for higher sample sizes (see Supplementary Fig. S3, for group sizes of 500 and 1,000). The power of distinct multiplicity correction methods only differed slightly. Hommel’s correction resembles the Bonferroni power curves but outperforms the classical Bonferroni correction in terms of power in all scenarios (not shown in the plots, see Supplementary Fig. S13 and S24).

### Weighted Bonferroni correction and binary composite endpoints

**Fig. 4:**
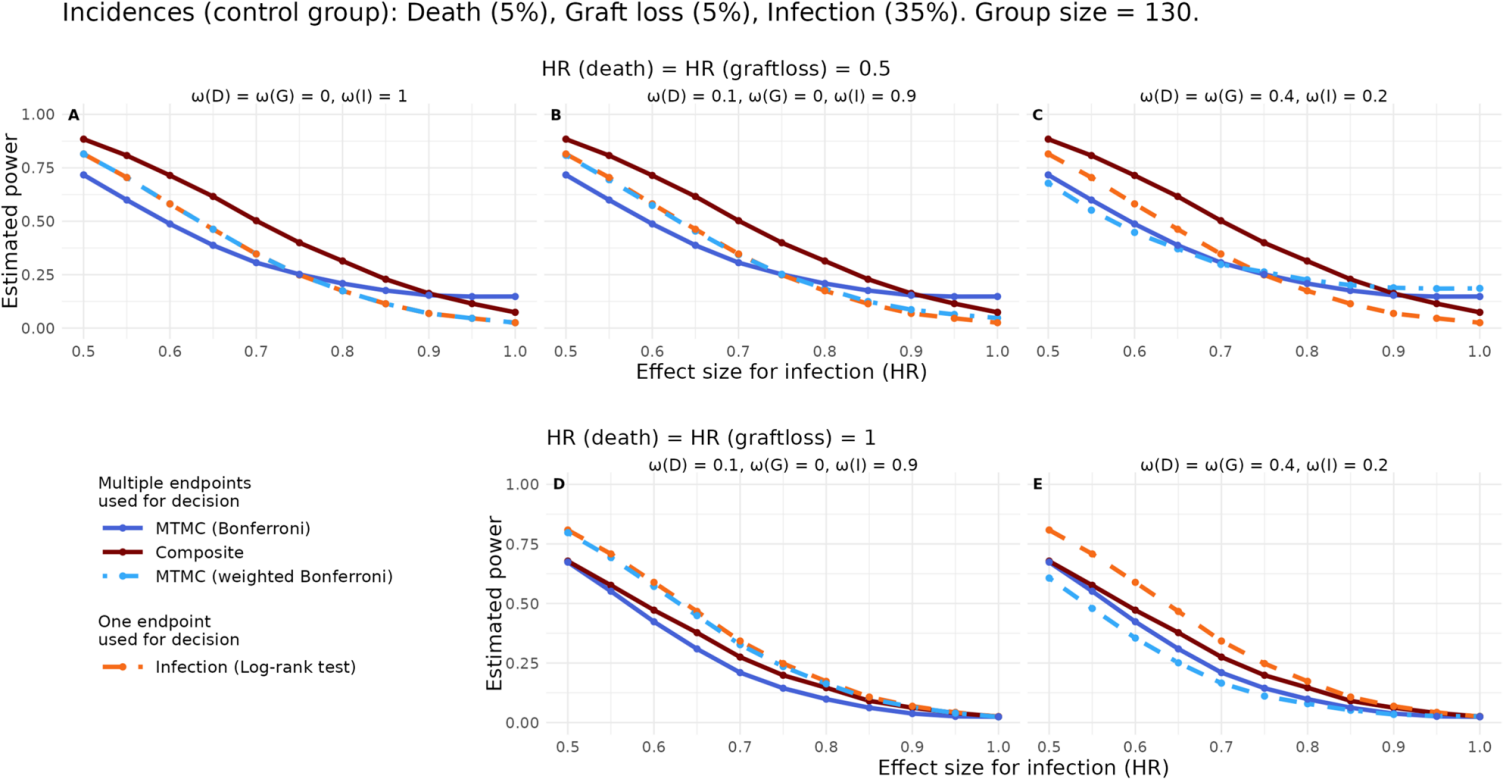
Comparison of estimated power of a weighted Bonferroni procedure and aforementioned approaches using one-sided tests in various scenarios. Time-to-event endpoint definitions are again used and differences between groups tested by log-rank tests. The expected proportion of deaths, graft losses and infections in the control group is fixed to 5%, 5% and 35%, respectively, in all shown plots. In the first row of plots, a marked treatment effect on graft loss and death (hazard ratio (HR) = 0.5) is present. In the lower plot, there is no treatment effect on graft loss nor death (HR = 1). The x-axis of plots depicts the HR of infections (0.5, a strong treatment effect, to 1, i.e., no effect). On the y-axis the estimated power is plotted. The weights, $\omega$ D (death), G (graft loss) and I (infection), applied to adjust the tests p-values varies and is given above each plot. Solid lines identify procedures taking all endpoints into account; Multiple testing and multiplicity correction (MTMC) using log-rank tests and Bonferroni correction (blue), Composite endpoint (darkred). Dashed lines indicate log-rank tests of infections (orange). Dashed-dotted lines are the weighted version of the Bonferroni correction (light blue).

Figure 4 shows the comparison of a weighted version of the Bonferroni correction to the composite endpoint and the classical (equally weighted) Bonferroni correction. In the first row of plots, different weighting of tests is depicted in a single scenario only varying the HR of infections on the x-axis. If all the weight is placed on one endpoint, power is trivially the same as that of a test of only this endpoint (here infections, Fig. 4A). Applying more weight to the other endpoints can enhance power in certain scenarios compared to the equally weighted Bonferroni correction. Apart from scenarios with low power overall (e.g. right hand side of plots in Fig. 4A–C), the power of the weighted correction does not exceed the power of testing the composite endpoint in our investigated scenarios. Other exceptions are scenarios as seen in Fig. 4D, E, where the power of the composite approach is inferior to testing one of the endpoints alone without correction (as could be done e.g. in a hierarchical testing procedure). This situation arises for example if treatment affects only one of the endpoints (here infections).

Additionally to defining a time-to-event composite endpoint (TTE CE), we define a binary composite endpoint (BIN CE) as whether a participant reaches any of the endpoints of interest within the observation time. The power of testing a TTE CE is very similar to that of testing the BIN CE, as exemplarily shown for some scenarios in Supplementary Fig. S4. The difference in power varies without obvious pattern between scenarios but never exceeds a few percentage points in our setting. Overall, there is a slight tendency towards superior power of the log-rank test.

### Opposing treatment effects

Another key question is how opposing treatment effects, i.e., situations in which the treatment affects one or more endpoints negatively, influence the overall trial decision. Overall, patterns similar to those observed in scenarios without opposing treatment effects emerge (see Supplementary Section C.3), but with important differences driven by the definition of the tested null hypotheses and prioritization of endpoints. Scenarios of particular interest are situations in which treatment reduces infections while deaths or graft losses occur more frequently.

Because the power of composite endpoints and even more so of GPC depends more strongly on higher-priority endpoints than does MTMC, these global strategies tend to yield fewer declarations of a positive treatment effect when opposing effects occur in more important endpoints. This reflects that adverse effects in higher-priority components directly reduce the probability of rejecting the corresponding global null hypothesis, even when beneficial effects are present in lower-priority endpoints. Exceptions arise in some scenarios with large treatment effects on infections combined with low event rates and small effect sizes for death.

In nearly all scenarios with a positive treatment effect on the driving endpoint (infections), the rejection probability of all three approaches remains well above the nominal significance level, indicating that the tested (global) null hypotheses remain under the alternative despite the presence of opposing effects in other components. For more details see Supplementary Section C.3.

### Endpoints without semi-competing risks and correlated endpoints

Results change only marginally if there is no terminal event like death. By design, the power of the composite endpoint is not affected by the different data generating mechanisms and also the GPC’s power does not change notably in any of the scenarios investigated. The MTMC’s power reduces slightly when the first endpoint is terminal. Simultaneously, power reductions of the tests of single endpoints two and three subject to the competing risk of the first endpoint are observed. For details see Supplement C.2 and Fig. S5.

**Fig. 5:**
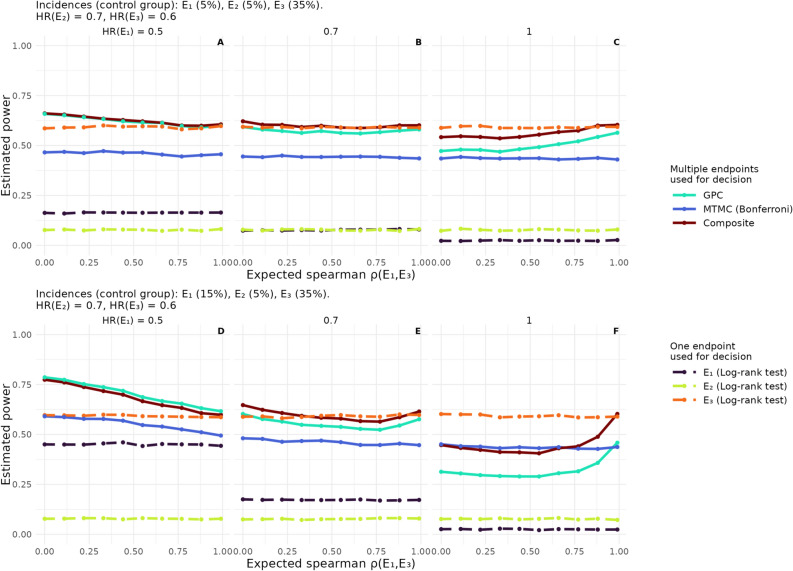
Influence of correlations among endpoints on the power of the compared methods. Time-to-event endpoint definitions are again used tested by one-sided log-rank tests. Note that there is no terminal event and thus the endpoints are generically labelled as $E_1$, $E_2$, and $E_3$. The expected proportion of the first endpoint, $E_1$, in the control group within follow-up is 5% in the first row of plots and 15% in the lower row. Endpoints $E_2$ and $E_3$ are fixed to 5% and 35%, respectively and their hazard ratios (HR) to 0.7 and 0.6. The treatment effect of the first endpoints decreases within each row from left to right from a HR of 0.5, a strong treatment effect, to one, i.e., no treatment effect. The x-axis of plots depicts the expected Spearman correlation of the first and last endpoint in the control group would no administrative censoring take place. On the y-axis the estimated power is plotted. Solid lines identify procedures taking all endpoints into account; Multiple testing and multiplicity correction (MTMC) using log-rank tests and Bonferroni correction (blue), Composite endpoint (darkred), GPC (turquoise). Dashed lines indicate log-rank tests of endpoints $E_1$ (black), $E_2$ (green), and $E_3$ (orange).

When endpoints are correlated, the way the correlations influence the methods’ power depends on the effect sizes of the involved endpoints (see Fig. 5). The relation is similar but more pronounced if there is less administrative censoring as can be seen by comparing the first and second row of plots in Fig. 5. If there are strong positive treatment effects in all endpoints, higher correlations between two of the endpoints lead to an decrease in power of all three approaches incorporating several endpoints while the power of tests of single endpoints is not altered (Fig. 5A, D). The results suggest a u-shaped dependence for moderate effect sizes of all endpoints (Fig. 5B, E). When there is no treatment effect in one of the correlated endpoints, the power increases with higher correlations (Fig. 5C, F) although Fig. 5F still suggests a u-shaped relation. The relation between correlations and power also depends on the incidences of endpoints. While increasing the incidence of one endpoint seems to amplify the patterns observed for lower incidences (compare first and second row of plots in Fig. 5), scenarios with high incidences of the first endpoint, $E_1$, for example yield a different relation (see Supplementary Section C.4 and also Fig. S16).

To aid interpretation, the Supplementary Material provides a structured exploration of additional simulation scenarios. Supplementary Section C examines in further figures the impact of larger group sizes, the semi-competing-risk structure, negative treatment effects on some endpoints and endpoint correlations in dedicated subsections. Supplementary Section D compares one-sided to two-sided testing. Throughout the main text, and in Supplementary Section C and D, we highlight a limited number of clinically relevant scenarios, while the Supplementary Section E allows readers to explore the full parameter space in detail.

## Discussion

The analysis and interpretation of multiple endpoints in clinical trials is of growing importance, particularly in studies investigating personalized immunosuppression in kidney transplantation. While chronic rejection remains a significant clinical problem, there has been limited innovation in immunosuppressive therapies, leading to increased interest in optimizing the use of existing treatments. This has stimulated the development of biomarker-guided strategies aimed at achieving an appropriate balance between efficacy and safety^15^. Regardless of the specific approach taken, the design of trials capable of validly demonstrating such a balance remains challenging, particularly under constraints of limited sample size and event numbers.

In this simulation study, we investigated the statistical power of three strategies for incorporating multiple endpoints into the primary analysis of kidney transplant trials. Although our focus was on settings relevant to immunosuppression studies, the approaches and our findings are not restricted to this context and generalize to other clinical trials involving endpoints with comparable properties and dependence structures, as well as to settings across diverse medical research areas.

We found the CE and GPC approaches to be favourable in terms of power. Notable exceptions can be found when the sample size is relatively large and the MTMC outperforms the other approaches while at the same time being reasonably powerful (i.e., more than 75%), see Supplementary section C.1. The semi-competing risk structure of our data—where death prevents us from observing other events afterwards—only notably affects the power of MTMC and should therefore be taken into account when comparing the approaches. The reductions in power are subtle in most situations.

Correlations between endpoints can seriously affect the power of all three strategies as described in the literature^6,18,40^. In this simulation study, the power of the GPC and CE was more heavily affected by positive correlations among endpoints than the MTMC’s power. Endpoint correlation is thus an important information when searching for the most powerful method. Furthermore, the relation of the GPC’s and CE’s power and correlations of endpoints depends on the effect sizes and incidences of the involved endpoints. This means that for certain scenarios power declines with increasing correlations, while in other scenarios we observed a u-shaped relation. A similar behaviour was observed by Verbeeck et al.^40^ for prioritized GPC and correlated components. For CE the observed proportions of binary composite endpoints decrease with increasing positive correlation between the components^18^. Our simulations suggest a similar mechanism for time-to-event composite endpoints: multiple independent endpoints provide more information than (moderately) correlated endpoints treatment effects are present across all components. More details investigating the impact of endpoint correlation, or opposing treatment effects are provided in the corresponding Supplementary section C.

CE and GPC are global strategies that combine information across multiple endpoints, but both become challenging when components differ in clinical importance, incidence, or direction of treatment effect^3,8,13,14^. In particular, when effects are heterogeneous or opposing across endpoints, both CE and GPC may yield statistically significant results even if a clinically important component is adversely affected. This follows from the tested null hypothesis (an overall treatment difference rather than consistent benefit across all components) and does not represent a failure of type I error control. Therefore, any positive global result should be accompanied by a careful examination of the individual component endpoints to support clinical interpretation and identify potential trade-offs, as recommended by regulatory guidance^3,5^. More complex objectives (e.g., superiority in at least one endpoint combined with non-inferiority in others) may address such concerns, but require additional design and analysis complexity^41–43^.

For CE, components are ideally of comparable clinical importance and affected by the intervention in a broadly similar direction^3,8,13,14^. In kidney transplantation, however, infections (or kidney function measures) may dominate the composite because death, graft loss, and rejection often have comparatively low incidence. Consistent with this, we found that the power of CE depends strongly on the treatment effect in this driving component, even though the components are not of equal clinical importance. This can complicate interpretation when a frequent but less severe component drives statistical significance while rarer, more severe components show smaller or even adverse effects.

GPC is often proposed to address unequal importance by prioritizing clinically more severe outcomes^13,14^. However, defining the hierarchical order can be ambiguous and substantially influences results^44,45^. As also observed in our simulations and noted previously, GPC depends particularly strongly on the highest-priority endpoints: if these are frequently occurring, but not positively affected, most pairwise comparisons terminate early, reducing the ability to detect potential benefits in lower-ranked components (e.g., improved infections when efficacy endpoints are similar)^8,36,46^. Thus, GPC may mitigate but does not eliminate concerns related to low-incidence clinically critical outcomes or opposing effects across endpoints^45,46^. At the same time, GPC remains attractive because it can combine endpoints measured on different scales, including continuous variables and patient-reported outcomes^12,44^. Finally, extensions incorporating recurrent events (e.g., infection counts) may further improve efficiency, but were beyond the scope of the present study^47–49^.

Both of the above-mentioned approaches have shortcomings regarding interpretation. CE and GPC produce a single global test-statistic summarizing multiple outcomes; a significant result indicates a difference in the CE or GPC endpoint, but it does not by itself identify which components contribute most to that difference. While we found that using MTMC reduced power in most of the scenarios, it conducts component-wise tests (with multiplicity adjustment), which can make it easier to attribute evidence of benefit (or harm) to specific endpoints. However, in the scenarios we investigated, tests of the most important endpoints with low event rates lack power to find potential harm of the treatment. Furthermore, the semi-competing risk relationships between the outcomes, the need for selection of a multiplicity correction method, and the ambiguous choices associated with weighting may limit the benefits of MTMC.

Surrogate endpoints are commonly used in oncology and kidney transplantation trials to shorten observation times when event rates are low, and endpoints such as eGFR have been proposed as surrogate endpoints in kidney transplantation^16^. In this work, however, the aim is not to identify or validate surrogate endpoints for overall survival. While some components may act as surrogates for hard outcomes such as survival, all three approaches also legitimately include outcomes that are clinically important in their own right, even if they are not surrogates^5^. In the motivating example, the considered components reflect distinct and clinically meaningful aspects of treatment benefit, such that improvement in any single component constitutes a relevant success.

We assumed a fixed follow-up time for all participants, reflecting trial designs in which outcomes are assessed at a pre-specified landmark time. Such fixed follow-up designs are common in many clinical study areas, including transplantation, cardiovascular disease, and infectious diseases, where, e.g., one-year graft survival or rejection, MACE at 12 or 24 months are standard primary endpoints. In these settings, fixed follow-up simplifies interpretation and aligns with regulatory expectations^8,50^. By contrast, in other clinical areas – most notably oncology – trial designs typically involve staggered patient entry and follow-up until a common target date, resulting in variable follow-up times across participants. The choice of fixed versus variable follow-up reflects fundamentally different clinical and regulatory questions rather than a methodological limitation. While here we focus on settings where fixed follow-up is clinically meaningful, extending the present simulation framework to incorporate recruitment patterns and variable follow-up would be a valuable direction for future research.

An important limitation of our results is that if the hazards change over time, e.g., hazard for death depends on the number of infections observed, then the proportional hazards assumption is violated^51,52^. How the strategies’ power is influenced by a violation of the proportional hazard assumption should thus be characterized in future research.

**Table 3:** Summary of investigated multiple endpoint approaches and their respective strengths and limitations.

Method	Main principle	Strengths	Limitations
Composite endpoint	Combines several endpoints into one (e.g., first of events to occur)	Increases statistical power Handles intercurrent events in endpoint definition	Components might not be of equal importance Power depends on effect size of the endpoint most frequently occurring Information about further events is discarded
Multiple testing with correction for multiplicity	Separate tests for each endpoint, adjust $\alpha$-level or p-values to control Type-I-error	Simplifies interpretation; Allows conclusion on individual endpoints	Reduces power Sensitive to how intercurrent events are addressed in single endpoints Sensitive to weighting
Generalized pairwise comparisons	Single nonparametric test statistic with pairwise comparisons of individual participants based on clinical importance	Naturally incorporates decision making on hierarchy of endpoints	Power highly dependent on effect sizes and occurrence of prioritized endpoints Interpretation

## Supplementary Information

Supplementary Information.

## Data Availability

The simulated datasets used and analysed during the current study are available from the corresponding author on reasonable request.

## Code availability

The R-code for all simulations conducted is available from the authors upon request.
