# Supplementary material for: Comparison of primary analysis strategies of randomized controlled trials with multiple endpoints with application to kidney transplantation
Source: Sci Rep. 2026 Feb 13;16:8769. doi: 10.1038/s41598-026-38979-6 (PMC12982691; doi:10.1038/s41598-026-38979-6)
Supplement: Supplementary file 1 — Supplementary Information. [file 41598_2026_38979_MOESM1_ESM.pdf]

Supplementary Material for Comparison of Primary Analysis Strategies of Randomized Controlled Trials with Multiple Endpoints with Application to Kidney Transplantation

Felix Herkner[1,2], Martin Posch[1], Gregor Bond[2], Franz König[1,*]

[1] Center for Medical Data Science, Medical University of Vienna, Vienna, Austria

[2] Division of Nephrology and Dialysis, Department of Medicine III, Medical University of Vienna, Vienna, Austria

[*] franz.koenig@meduniwien.ac.at

2026-01-16

Contents

A Methods - additional information **3**
A.1 Details on the data generating mechanisms . . . 3
A.1.1 Uncorrelated endpoints, semi-competing risk of death . . . 3
A.1.2 Correlations between components, no terminal event . . . 3
A.1.3 Uncorrelated endpoints, no terminal event . . . 4
A.2 Deriving simulation parameters . . . 4
A.3 Statistical methods - additional information . . . 4
A.3.1 Definition of one- and two-sided hypotheses . . . 4
A.3.2 Definition of one- and two-sided p-values and implementation of tests . . . 4
A.4 Details of Clinical Trials Simulation Setup (Parameter specification and Scenarios) . . . 5
A.5 Performance measures for clinical trial simulations . . . 9

B Additional results and information on the simulated case studies **10**
B.1 Visualisation of simulated case studies . . . 10
B.2 Additional results using one-sided tests (superiority) . . . 13
B.3 Additional results using two-sided tests (differences) . . . 15

C Additional results investigating the impact of different data generating mechanisms including sample sizes, effect sizes, correlations and type of statistical tests **17**
C.1 Larger group sizes and binary composite endpoint . . . 17
C.2 No terminal event . . . 20
C.3 Opposing treatment effects . . . 22
C.4 Additional information concerning correlated endpoints . . . 26

D One-sided (superiority) versus two-sided (difference) testing **28**

E Overview of results of all investigated scenarios **31**
E.1 Plots of all scenarios evaluated (one-sided tests) . . . 31
E.2 Plots of all scenarios evaluated (two-sided tests) . . . 43

F List of abbreviations used **49**

Overview

This Supplementary Material provides additional methodological details and extended simulation results supporting the main manuscript. It is organized as follows:

A **Methods - additional information:** Details for statistical methods, data generating mechanisms, how simulation parameters are specified and the simulation setup are provided. Section A.4 also gives an overview over the scenarios that were evaluated.

B **Additional results and information on the simulated case studies:** Visualisation of the simulated study results are shown. Additional results for the simulated case studies of Section "Motivating example revisited: Examples of two simulated studies" of the main paper are provided. Extension include two-sided tests and results for testing binary composite endpoints.

C **Additional results investigating the impact of different data generating mechanisms including sample sizes, effect sizes, correlation between components and type of statistical tests:** Supporting information and results for different data generating mechanisms and setting are provided. The basis for the shown graphs are the scenarios shown in Figure 3, 4 and 5 in the main paper.

D **One-sided (superiority) versus two-sided (difference) testing:** A direct comparison of power when testing superiority (one-sided) versus differences (two-sided) in settings of the main paper Figure 3 is shown. Power estimations for two-sided tests are provided for Figure 4 of the main paper.

E **Overview of results of all investigated scenarios:** Results of all investigated scenarios are provided for both one- and two-sided tests.

F **List of abbreviations used:** A table is provided including all abbreviations used both in the main manuscript and supplement in alphabetical order.

IMPORTANT NOTES

- To show the impact of varying a single parameter (like the effect size of one endpoint) we used animated figures. Animated slide shows when there are several Figures with the same structure. These are recognised by arrows beneath the picture (see the following screenshot). Use the arrows to navigate the slide show.

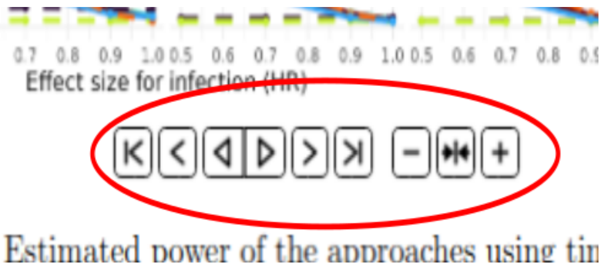

- If the slide shows do not work, please try to switch to another PDF reader (tested for Adobe Acrobat).
- In all plots in the supplement we use the notation D, G, and I, for endpoints death, graft loss and infection, respectively, if there is a semi-competing risk structure, i.e., death prevents us from observing the other endpoints. Results of data generating mechanisms without terminal event use the notation E_1, E_2, and E_3 for the endpoints.

A Methods - additional information

A.1 Details on the data generating mechanisms

As introduced in the methods section of the main manuscript, three data generating mechanisms were considered: (a) uncorrelated endpoints but one of the endpoints (death) prohibits observation of other events occurring later, (b) endpoints where no such terminal endpoint exists but correlations among the endpoints are present, and (c) completely unrelated endpoints without terminal events nor correlations. The details of each data generating mechanism are presented in the next three subsections.

A.1.1 Uncorrelated endpoints, semi-competing risk of death

Three independent exponentially distributed variables are generated using base R's "rexp" function [9], representing latent time to death (D_{orig}), time to graft loss (G_{orig}) and time to first infection ($I_{1,orig}$). The distributions use parameters λ_d, λ_g and λ_i, respectively, i.e. $D_{orig} \sim Exp(\lambda_d)$, $G_{orig} \sim Exp(\lambda_g)$ and $I_{1,orig} \sim Exp(\lambda_i)$.

Participants are administratively censored at the fixed follow-up time, s. The time-to-event (TTE) composite endpoint is then defined as time to the first of the events to be experienced by a participant, if it is observed within follow-up: $TTE_{composite} = min[D_{orig}, G_{orig}, I_{1,orig}, s]$. If participants experience the event within their follow-up (i.e. $TTE_{composite} < s$) they are marked as event and censored otherwise. A composite binary endpoint is defined by

$$Bin_{composite} = \begin{cases} 1 & \text{if } TTE_{composite} < s \\ 0 & \text{otherwise} \end{cases} \tag{S1.1}$$

where s is the administrative censoring time that is equal for all participants. Note that no dropouts are simulated.

The time-to-event for the endpoint death is leaving D_{orig} unchanged (administrative censoring still applies; $D = min[D_{orig}, s]$). As people cannot loose their graft or be infected after dying, the two other endpoints are defined $G = min[G_{orig}, D_{orig}, s]$ and $I = min[I_{1,orig}, D_{orig}, s]$. The status variable indicating events for G is then defined

$$Status_G = \begin{cases} 1 & \text{if } G = G_{orig} \\ 2 & \text{if } G = D_{orig} \\ 0 & \text{otherwise} \end{cases} \tag{S1.2}$$

and likewise for $Status_I$. The distinction of events allows for flexible handling of deaths occurring before one of the other events (e.g. censoring at time of death, or applying other competing risk methodology).

Times-to-rejection in Section "Motivating example revisited: Examples of two simulated studies" are generated in the same way as the other endpoints ($R_{orig} \sim Exp(\lambda_r)$ and accordingly included in the composite endpoint definitions. Observable time-to-event data for rejections take into account that rejections are both censored by death and graft loss: $R = min[R_{orig}, G_{orig}, D_{orig}, s]$ and the status variable extends Equation (S1.2) to "1" marking rejections, "2" graft losses and "3" deaths, "0" otherwise.

A.1.2 Correlations between components, no terminal event

To generate correlated endpoints E_1, E_2, and E_3, the copula package [6] was used. The naming of endpoints deviates from the rest of the simulations to emphasise that there is no terminal event like death among the endpoints. The desired Spearman's $\rho_S = [\rho_S(E_1, E_2), \rho_S(E_1, E_3), \rho_S(E_2, E_3)]$ for each pair of endpoints was set and transformed to Pearson's $\rho_P = [\rho_P(E_1, E_2), \rho_P(E_1, E_3), \rho_P(E_2, E_3)]$ via the relation

$$\rho_P = 2 * sin(\frac{\pi}{6 * \rho_S}) \tag{S1.3}$$

where the ρ_P values are then passed to the normalCopula function. Exponential marginal distributions for each endpoint and each group are set within the mvdc function and the event times for each participant and simulation repetition then drawn from the rMvdc function. Administrative censoring is again performed at the end of the fixed follow up, see above.

A.1.3 Uncorrelated endpoints, no terminal event

When there is neither a semi-competing risk structure nor correlations, independent latent exponential variables were generated and only administrative censoring at the fixed follow-up time s applied. The composite endpoint is again the time to the first event and is thus the same as in the first data generating mechanism. The single endpoints, on the other hand, do change.

A.2 Deriving simulation parameters

Expected proportions of events in the control group can be found using properties of the underlying distributions as follows. As death in the simplest setting is unaffected by the other endpoints, the expected proportion of death in the control group is

$$\mathbb{P}(D_{orig} < s) = 1 - e^{-\lambda_d s} \tag{S1.4}$$

It is a well-known result that the minimum of n exponentially distributed variables with parameters $\lambda_1, ..., \lambda_n$ is again exponential with parameter $\lambda = \sum_{i=1}^{n} \lambda_i$. The expected proportion of graft loss in the control group within follow-up is then

$$\begin{aligned} \mathbb{P}(min(G_{orig}, D_{orig}, s) = G_{orig}) &= \mathbb{P}(min(G_{orig}, D_{orig}) \leq s, min(G_{orig}, D_{orig}) = G_{orig}) \\ &= \int_0^s f_G(x)\mathbb{P}(D_{orig} > x)dx \\ &= \lambda_g \int_0^s e^{-(\lambda_g+\lambda_d)x}dx \\ &= \frac{\lambda_g}{\lambda_g + \lambda_d}(1 - e^{-s(\lambda_g+\lambda_d)}) \end{aligned} \tag{S1.5}$$

where λ_g is determined numerically (e.g., by bisection) to match the desired incidence. In this way one can set λ_g so as to achieve a desired expected proportion of graft losses in the control group taking into account censoring by death.

In the same way, the parameters of the exponential distributions of infections and rejections can be calculated by setting the desired expected proportions in the control group.

A.3 Statistical methods - additional information

A.3.1 Definition of one- and two-sided hypotheses

Supplementary Table S1 gives an overview over hypotheses used for testing differences between arms (two-sided tests) and superiority of the treatment (one-sided tests).

Type of test	Composite endpoint	GPC	MTMC
Difference	$H_0 : h^{(T)}_{Comp}(t) = h^{(C)}_{Comp}(t)$ $\forall \quad 0 < t < s$	$H_0 : NB = 0$	$H_0 : h^{(T)}_i(t) = h^{(C)}_i(t)$ $\forall \quad 0 < t < s$ and all i = {1,2,3}
Superiority	$H_0 : h^{(T)}_{Comp}(t) \leq h^{(C)}_{Comp}(t)$ $\forall \quad 0 < t < s$	$H_0 : NB \leq 0$	$H_0 : h^{(T)}_i(t) \leq h^{(C)}_i(t)$ $\forall \quad 0 < t < s$ and all i = {1,2,3}
Terminology	h^j_{Comp} is the hazard at time t of reaching the composite endpoint in arm $j = \{T, C\}$. s is the fixed follow-up time.	NB is the Net Treatment Benefit	$h^j_i(t)$ is the hazard at time t of reaching the endpoint i in arm $j = \{T, C\}$. s is the fixed follow-up time.

Supplementary Table S1: Null hypotheses of testing strategies for difference between groups (two-sided tests) and superiority of the treatment group (one-sided test) for time-to-event endpoints.

A.3.2 Definition of one- and two-sided p-values and implementation of tests

One sided p-values p_S (shown in the simulated case studies, Section "Motivating example revisited: Examples of two simulated studies" of the main paper and Supplement Section "Details on the simulated

case studies") were derived from two-sided p-values, p_D, of log-rank, χ^2 and GPC tests as follows:

$$p_S = \begin{cases} \frac{p_D}{2} & \text{if the effect estimate is favourable for the treatment} \\ 1 - \frac{p_D}{2} & \text{otherwise} \end{cases}$$

Note, if the effect estimate would be exactly neutral (e.g., observed HR = 1 or observed NTB = 0), we could set the one-sided p-values to $p_S = 0.5$ (or equivalently, treat it as not favourable). The one-sided p-values p_S are then compared to a one-sided 2.5% significance level. This is equivalent to declare a trial success if the two-sided p-value was $p_D < 0.05$ and the treatment effect estimate favourable for the treatment group, as was done in the simulations. Treatment effect estimates were the hazard ratios in case of log rank tests, difference in proportion in case of χ^2-tests, and the net treatment benefit in case of the GPC (see also the hypotheses associated with each test in Supplement Table S1).

The hypotheses tested by a two-sided Gray's test for differences in CIFs are $H_0 : CIF_T(t) = CIF_C(t) \quad \forall t$ versus $H_1 : CIF_T(t) \neq CIF_C(t)$. We did not consider a one-sided version of Gray's test.

A.4 Details of Clinical Trials Simulation Setup (Parameter specification and Scenarios)

An overview of parameters and their values that were investigated are given in Supplementary Table S2-S4 for each data generating mechanism. Following the terminology introduced by Benda et al. [1] and Friede et al. [3], parameters of clinical trial simulations may be classified as disease specific features (DSF) or design choices (DC). DSF may be estimable from preliminary data (DSF (e), like incidences of endpoints) or must be assumed entirely (DSF (a), like underlying patterns of missing data). DC might be constrained (DC (c)) due to, e.g., external factors.

The weighted Bonferroni correction is evaluated in selected scenarios with uncorrelated endpoints and terminal event of death and every combination of weights applied to the endpoints. Selected scenarios are expected proportions of death and graft loss of 5% and infections 35% in the control group (which is the setting expected to closest reflect real study conditions in kidney transplant immunosuppression studies following up patients in the first year after transplantation) and every combination HRs 0.5, 0.7 and 1 for each of the endpoints (resulting in 27 scenarios). An additional 16 scenarios were again run to provide smoother plots. For each of these scenarios, every possible combination of weights is applied as specified in Supplementary Table S3, see the Plot grids in Supplementary Fig. S14. As the weights must sum to one, 66 possible combinations of weights in every single scenario are calculated. In the additional scenarios, not all weights were applied.

In total, 771 scenarios were investigated for group sizes of 130 both treating death as terminal event and without such relations among endpoints (see Supplementary Fig. S13 and S15). Additional 1113 scenarios accounting for informative censoring for group sizes 250, 500 and 1,000 were simulated (see Supplementary Fig. S20-S23). For 66 of these combinations of hazard ratios and incidences of endpoints, different combinations of correlations among endpoints were investigated, yielding a total of 4620 scenarios (see Supplementary Fig. S16-S18 and S10). To investigate opposing treatment effects, another 9678 scenarios were investigated where at least one of the treatment effects were in favour of the control group (see Supplementary Fig. S19). Weighting of tests was investigated in 43 scenarios (Supplementary Fig. S14).

Supplementary Table S2: General important parameters used in all simulations and their specification. DSF (e) = estimable Disease Specific Features, DSF (a) assumed DSF. DC = Design choices, and DC (c) = constrained DC. MCSE = Monte Carlo standard error, CE = Composite endpoint, MTMC = Multiple testing and multiplicity correction, GPC = Generalized pairwise comparisons.

Parameter	Type	Investigated values	Description
Individual follow-up time	DC	10 months	Fixed follow-up per participant
Simulation repetitions	DC	10,000	Number of simulation repetitions. Calculated to meet a reasonable MCSE for power estimates of $<$ 0.005 (see Supplementary Section A.5)
Statistical significance level α	DC	5% (two-sided), 2.5% (one-sided)	The significance level used for two-sided tests and one-sided tests, respectively
Type of endpoints	DC	Time-to-event, binary	Binary endpoints only reported for the composite endpoint
Testing strategies and endpoint definitions	DC	(i) CE	Time-to-event (log rank test) and binary (χ^2 test)
		(ii) MTMC	Time-to-event (log rank tests, Gray's test)
		(iii) GPC	Time-to-event
Hypothesis tests	DC	Log rank test	Implemented in the *survival* R package [11]
		Gray's test	Implemented in the *cmprsk* R package [5]. Only two-sided tests for differences between groups were investigated.
		GPC	Implemented in the *BuyseTest* R package [8]
		χ^2 test	Implemented in the [9]

Supplementary Table S3: Important parameters and their specification used in scenarios with and without semi-competing risk of death and without correlations. DSF (e) = estimable Disease Specific Features, DSF (a) assumed DSF. DC = Design choices, and DC (c) = constrained DC. MCSE = Monte Carlo standard error, CE = Composite endpoint, MTMC = Multiple testing and multiplicity correction, GPC = Generalized pairwise comparisons.

Parameter	Type	Investigated values	Description
**Uncorrelated endpoints, semi-competing risk of death**			
Expected proportion of death within follow-up	DSF (e)	5%, 15%, (35%) (control group)	Expected proportion of deaths in the control group within observation period. Incidences of 35% only investigated for certain effect sizes and death being terminal
Expected proportion of graft loss within follow-up	DSF (e)	5%, 15%, (35%) (control group)	Expected proportion of graft losses in the control group within observation period taking into account censoring by death. Incidences of 35% only investigated for certain effect sizes and death being terminal
Expected proportion of infections within follow-up	DSF (e)	20%, 35% (control group)	Expected proportion of infections in the control group within observation period taking into account censoring by death
Hazard ratios (HR)	DSF (e)	0.5, ..., 1, ..., 1.5	Ranging from treatment being considered strongly favourable (HR = 0.5) to no effect (HR = 1) to opposing treatment effects (HR > 1) for each endpoint separately
Bonferroni weights	DC	0, 0.1, 0.2, ..., 0.9, 1	Weights to be used for each endpoint, if weights are used to split the alpha level
Group size	DC (c)	130, 250, 500, 1000	Sample size per group

Supplementary Table S4: Important parameters and their specification used in scenarios with correlated endpoints without semi-competing-risk structure. DSF (e) = estimable Disease Specific Features, DSF (a) assumed DSF. DC = Design choices, and DC (c) = constrained DC. MCSE = Monte Carlo standard error, CE = Composite endpoint, MTMC = Multiple testing and multiplicity correction, GPC = Generalized pairwise comparisons.

Parameter	Type	Investigated values	Description
**Correlated endpoints, no semi-competing risk of death**			
Combinations of expected proportions of endpoints	DSF (e)	E_1 / E_2 / E_3:<br>5% / 5% / 35%<br>15% / 5% / 35%<br>40% / 40% / 98%<br>98% / 50% / 35%	Expected proportion of single endpoints in the control group within the observation period
Hazard ratios (HR)	DSF (e)	0.5, ..., 1	Ranging from treatment being considered strongly favourable (HR = 0.5) to no effect (HR = 1) for each endpoint separately. Only a fraction of possible effect size combinations was used
Spearman rank correlations	DSF (e)	0, ..., 1	Set Spearman correlations for each pair of endpoints. Observed correlations differ because of administrative censoring. The correlations between E_2 and E_3 are a result of the other correlations and not explicitly set
Group size	DC (c)	130	Sample size per group

A.5 Performance measures for clinical trial simulations

The main goal of the study is to estimate the probability of rightfully rejecting the global null hypothesis if there is a treatment effect on at least one of the endpoints. If treatment does not affect any of the endpoints, the rejection probability corresponds to the type-1-error rate. This quantity for each of the strategies in each scenario is estimated by the proportion, $\hat{p}$, of simulation repetitions in which the overall null hypothesis is rejected and therefore the decision is made to declare a statistical significant treatment effect between the study arms. Estimates from simulation studies are subject to uncertainty despite the great number of repetitions, and suitable measures of uncertainty should thus be reported [7]. This is commonly done by computing the Monte Carlo Standard Error (MCSE), that is in this case the standard error of the estimate of statistical power over all simulation repetitions in one scenario. Using a normal approximation of a binomial distribution, the MCSE of the power estimates are calculated as $MCSE = \sqrt{\frac{\hat{p}(1-\hat{p})}{n}}$, where n is the number of simulation repetitions. 10000 simulation repetitions result in standard errors of 0.005 and 0.004 for $\hat{p} = 0.5$ (where the standard error is maximal) and $\hat{p} = 0.8$, respectively.

B Additional results and information on the simulated case studies

Additional figures, results and details of the scenario parameters used in the Section "Motivating example revisited: Examples of two simulated studies" of the main manuscript are provided for the two simulated case studies.

B.1 Visualisation of simulated case studies

In the first simulated case study example, the expected proportions of death (D), graft loss (G) and rejections (R) in the control group are set to 5% and infections (I) 35%, the (time-constant) hazard ratios (HR) between the treatment groups are chosen to be 1 (no difference between groups) for D, and 0.5 for G, R and I (corresponding to a fairly strong treatment effect between groups).

For the second example, the expected proportion of D in the control group is set to be 15%, while the respective proportions of G, R and I are the same as previously (5%, 5% and 35%, respectively). The HRs are now set to 1 (D), 0.7 (G), 1 (R) and 0.5 (I).

Note that while the follow-up time for each participant is set to $s = 10$ time units in the simulation study, a follow-up time of $s = 9$ is used in the examples. This only influences the choice of the parameters of exponential distributions because these are find to reach the desired incidences (see A).

The resulting cumulative incidence functions (CIF) in the two examples of different composite endpoint definitions can be seen in Fig. S1.

The CIF plots of the individual endpoints in the two simulated case studies in Section "Motivating example revisited: Examples of two simulated studies" are given in Supplementary Fig. S2.

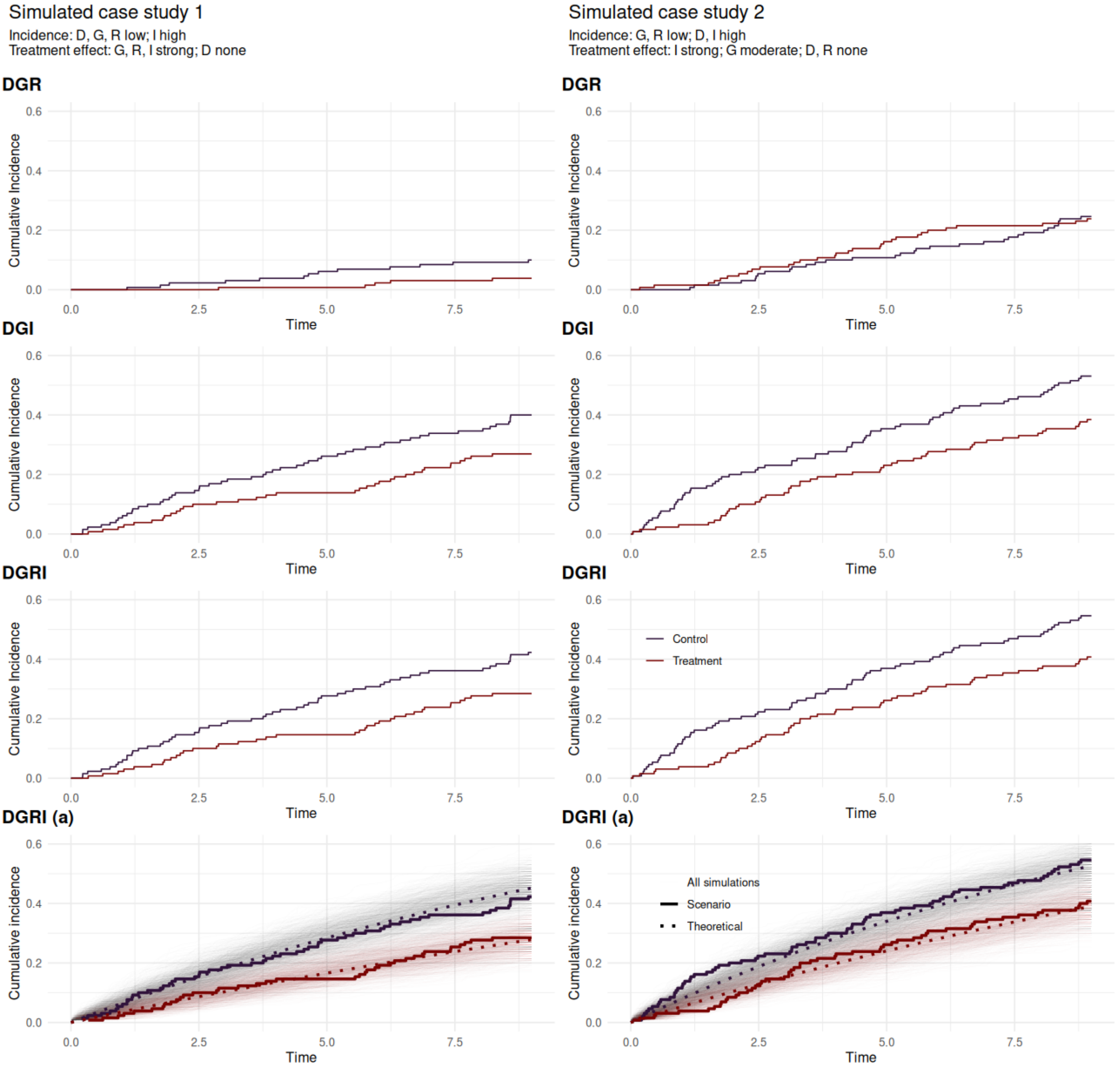

Supplementary Figure S1: Cumulative incidence function (CIF) curves of different composite endpoint combinations in simulated case study 1 (left column) and 2 (right column). The plot labels are the combination of endpoints considered in the respective composite: death (D), graft loss (G), rejections (R), or infections (I). E.g. "DGR" means that D, G, and R were combined in a composite endpoint. The group sizes are 130 in both scenarios, the follow-up time is 9 time units. Scenario 1: underlying expected proportions in the control group for D, G and R are set to 5%, I 35%, and hazard ratios (HR) between groups set to 1 (D) and 0.5 (G, R and I). Scenario 2: expected proportions in the control group are set to 15%, 5%, 5% and 35%, and HRs set to 1, 0.7, 1, 0.5 for D, G, R and I, respectively. The marked lines in the last row (plots DGRI (a)) give the CIF curves of the chosen study out of all simulation repetitions for the endpoint consisting of D, G, R, and I, the transparent lines in the background display all CIF curves of 1,000 simulation repetitions. The dotted lines are the theoretical CIF curves (calculated using properties of exponential distributions, see Supplementary Section A). Red lines depict estimates of the treatment arm, blue lines show the respective quantities in the control group.

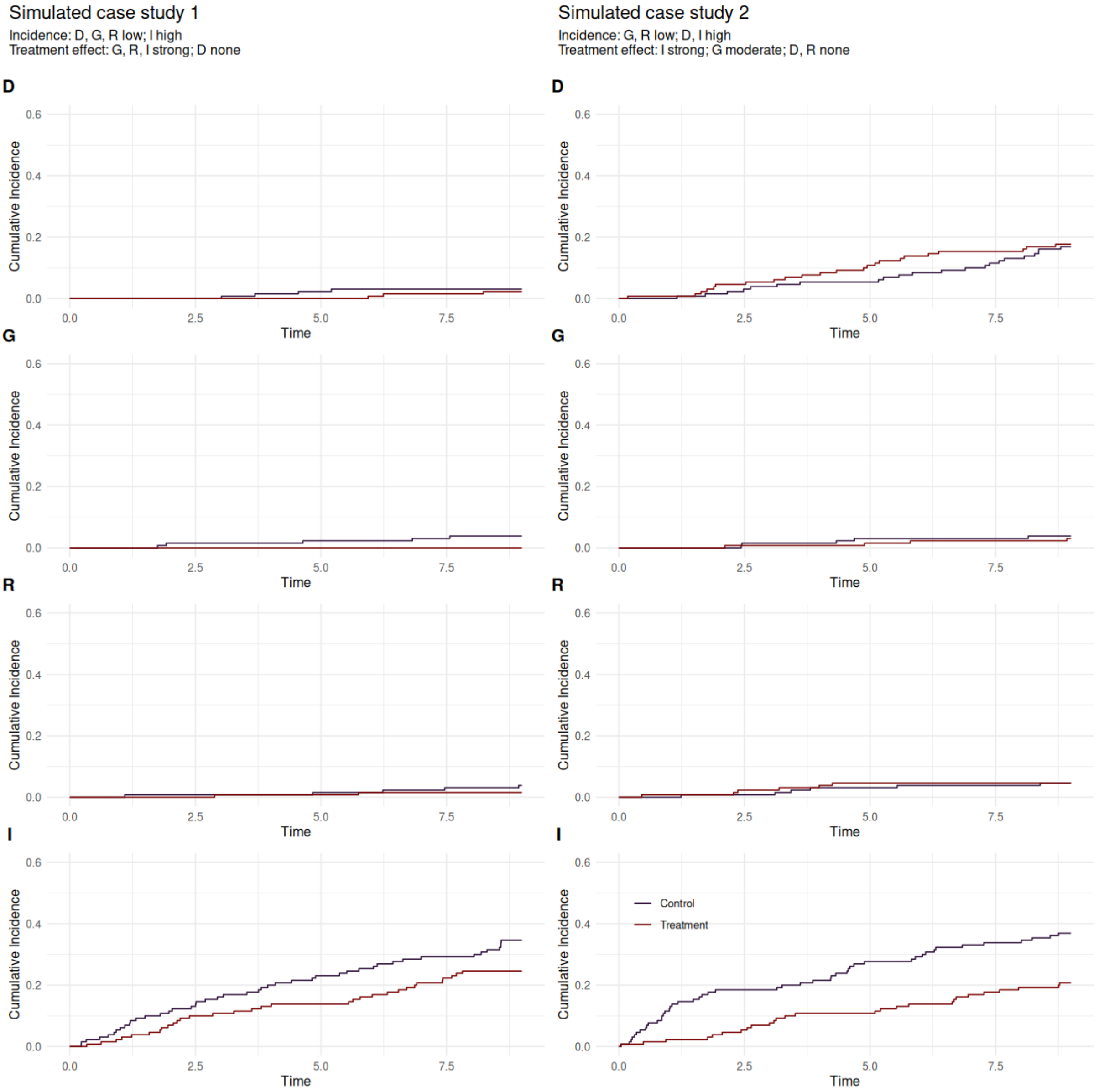

Supplementary Figure S2: Cumulative incidence functions (CIF) of individual endpoints in simulated case study 1 (left column) and 2 (right column). The x-axis depicts the time on study, the y-axis the cumulative incidence. Note that the y-axis is limited to 0.6. The endpoints are death (D, first row), graft loss (G, second row), rejections (R) and infections (I). In blue, the CIF of the respective endpoint in the control group is given, in red the CIF of the treatment arm. The group sizes are 130 in both scenarios, the follow-up time is 9 time units. Scenario 1: underlying expected proportions in the control group for D, G and R are set to 5%, I 35%, and hazard ratios (HR) between groups set to 1 (D) and 0.5 (G, R and I). Scenario 2: expected proportions in the control group are set to 15%, 5%, 5% and 35%, and HRs set to 1, 0.7, 1, 0.5 for D, G, R and I, respectively.

B.2 Additional results using one-sided tests (superiority)

Extending Table 2 in the main paper, in Supplementary Table S6 also the p-values of testing a binary composite endpoint via χ^2 tests are reported. The rest of the table is the same as Table 2 in the main paper.

Supplementary Table S5: Overview of the analysis using one-sided tests of simulated studies chosen from scenarios 1 and 2, respectively, including χ^2 tests of binary composite endpoints.

	**Simulated case study 1**<br>*Incidence: D, G, R low; I high*<br>*Treatment effect: G, R, I strong; D none*			**Simulated case study 2**<br>*Incidence: G, R low; D, I high*<br>*Treatment effect: I strong; G moderate; D, R none*		
	Control	Treatment	p-value[3]	Control	Treatment	p-value[3]
Death *n (%)*	4 (3.1%)	3 (2.3%)	0.3445	22 (16.9%)	23 (17.7%)	0.6054
Graftloss *n (%)*	5 (3.8%)	0 (0%)	**0.0117**	5 (3.8%)	4 (3.1%)	0.3843
Rejection *n (%)*	5 (3.8%)	2 (1.5%)	0.1169	6 (4.6%)	6 (4.6%)	0.5136
Infection *n (%)*	45 (34.6%)	32 (24.6%)	0.0352	48 (36.9%)	27 (20.8%)	**0.0014**
**Endpoints considered: D, G, R**						
Composite *RMST*[1]	8.56	8.88	**0.0244**	8.07	7.83	0.4982
BIN CE *n (%)*	13 (10%)	5 (3.8%)	0.0253	32 (24.6%)	31 (23.8%)	0.44245
MTMC	-	-	0.0351	-	-	1
GPC	e% / f% / u%[2]		**0.0247**	e% / f% / u%[2]		0.5376
Death	100% / 3.1% / 2.2%			100% / 14.9% / 16.7%		
Graft loss	94.7% / 3.8% / 0%			68.4% / 3.1% / 2.5%		
Rejection	90.9% / 3% / 1.4%			62.8% / 3% / 2.4%		
Ties left	86.54%			57.41%		
**Endpoints considered: D, G, I**						
Composite *RMST*[1]	6.99	7.75	**0.0108**	6.24	7.21	**0.0057**
BIN CE *n (%)*	52 (40%)	35 (26.9%)	**0.0128**	69 (53.1%)	50 (38.5%)	**0.0090**
MTMC	-	-	0.0351	-	-	**0.0042**
GPC	e% / f% / u%[2]		**0.0083**	e% / f% / u%[2]		0.0282
Death	100% / 3.1% / 2.2%			100% / 14.9% / 16.7%		
Graft loss	94.7% / 3.8% / 0%			68.4% / 3.1% / 2.5%		
Infection	90.9% / 28.4% / 18.6%			62.8% / 23.8% / 10.1%		
Ties left	43.85%			28.88%		
**Endpoints considered: D, G, R, I**						
Composite *RMST*[1]	6.89	7.68	**0.0081**	6.14	7.02	**0.0094**
BIN CE *n (%)*	55 (42.3%)	37 (28.5%)	**0.0098**	71 (54.6%)	53 (40.8%)	**0.0127**
MTMC	-	-	0.0468	-	-	**0.0056**
GPC[2]	e% / f% / u% [2]		**0.0056**	e% / f% / u%[2]		0.0403
Death	100% / 3.1% / 2.2%			100% / 14.9% / 16.7%		
Graft loss	94.7% / 3.8% / 0%			68.4% / 3.1% / 2.5%		
Rejection	90.9% / 3% / 1.4%			62.8% / 3% / 2.4%		
Infection	86.5% / 27.3% / 18%			57.4% / 21.4% / 9.2%		
Ties left	41.27%			26.88%		

[1] RMST = Restricted mean event-free survival time, restricted to 9 time units

[2] GPC pairs of the respective endpoint: e%: pairs evaluated / f%: favourable pairs / u%: unfavourable pairs. Percentages are fractions of ALL pairs.

[3] The first four one-sided p-values are calculated from log-rank tests of the individual endpoints. The composite time-to-event endpoint is tested using a log-rank test (one-sided). For MTMC, the smallest Bonferroni-adjusted one-sided p-value is reported. The overall one-sided p-value of the GPC is reported. Significant p-values (< 0.025) are bold.

D = Death, G = Graft loss, R = Rejections, I = Infections, MTMC = Multiple testing and multiplicity correction, GPC = Generalized pairwise comparisons

B.3 Additional results using two-sided tests (differences)

Supplement Table S6 gives the exact same calculations shown in Supplement Table S5 repeated for two-sided tests for differences between groups. The calculation of one- and two-sided p-values is discussed in Supplement section A.3.

Supplementary Table S6: Overview of the analysis using two-sided tests of simulated studies chosen from scenarios 1 and 2, respectively, including χ^2 tests of binary composite endpoints.

	**Simulated case study 1**<br>*Incidence: D, G, R low; I high*<br>*Treatment effect: G, R, I strong; D none*			**Simulated case study 2**<br>*Incidence: G, R low; D, I high*<br>*Treatment effect: I strong; G moderate; D, R none*		
	Control	Treatment	p-value[3]	Control	Treatment	p-value[3]
Death *n (%)*	4 (3.1%)	3 (2.3%)	0.6891	22 (16.9%)	23 (17.7%)	0.7891
Graftloss *n (%)*	5 (3.8%)	0 (0%)	**0.0234**	5 (3.8%)	4 (3.1%)	0.7687
Rejection *n (%)*	5 (3.8%)	2 (1.5%)	0.2338	6 (4.6%)	6 (4.6%)	0.9729
Infection *n (%)*	45 (34.6%)	32 (24.6%)	0.0704	48 (36.9%)	27 (20.8%)	**0.0028**
**Endpoints considered: D, G, R**						
Composite *RMST*[1]	8.56	8.88	**0.0488**	8.07	7.83	0.9963
BIN CE *n (%)*	13 (10%)	5 (3.8%)	0.0506	32 (24.6%)	31 (23.8%)	0.8849
MTMC	-	-	0.0703	-	-	1
GPC	e% / f% / u%[2]		**0.0495**	e% / f% / u%[2]		0.9248
Death	100% / 3.1% / 2.2%			100% / 14.9% / 16.7%		
Graft loss	94.7% / 3.8% / 0%			68.4% / 3.1% / 2.5%		
Rejection	90.9% / 3% / 1.4%			62.8% / 3% / 2.4%		
Ties left	86.54%			57.41%		
**Endpoints considered: D, G, I**						
Composite *RMST*[1]	6.99	7.75	**0.0216**	6.24	7.21	**0.0114**
BIN CE *n (%)*	52 (40%)	35 (26.9%)	**0.0255**	69 (53.1%)	50 (38.5%)	**0.018**
MTMC	-	-	0.0703	-	-	**0.0085**
GPC	e% / f% / u%[2]		**0.0165**	e% / f% / u%[2]		0.0563
Death	100% / 3.1% / 2.2%			100% / 14.9% / 16.7%		
Graft loss	94.7% / 3.8% / 0%			68.4% / 3.1% / 2.5%		
Infection	90.9% / 28.4% / 18.6%			62.8% / 23.8% / 10.1%		
Ties left	43.85%			28.88%		
**Endpoints considered: D, G, R, I**						
Composite *RMST*[1]	6.89	7.68	**0.0163**	6.14	7.02	**0.0189**
BIN CE *n (%)*	55 (42.3%)	37 (28.5%)	**0.0196**	71 (54.6%)	53 (40.8%)	**0.0254**
MTMC	-	-	0.0937	-	-	**0.0113**
GPC[2]	e% / f% / u% [2]		**0.0111**	e% / f% / u%[2]		0.0806
Death	100% / 3.1% / 2.2%			100% / 14.9% / 16.7%		
Graft loss	94.7% / 3.8% / 0%			68.4% / 3.1% / 2.5%		
Rejection	90.9% / 3% / 1.4%			62.8% / 3% / 2.4%		
Infection	86.5% / 27.3% / 18%			57.4% / 21.4% / 9.2%		
Ties left	41.27%			26.88%		

[1] RMST = Restricted mean event-free survival time, restricted to 9 time units

[2] GPC pairs of the respective endpoint: e%: pairs evaluated / f%: favourable pairs / u%: unfavourable pairs. Percentages are fractions of ALL pairs.

[3] The first four two-sided p-values are calculated from log-rank tests of the individual endpoints. The composite time-to-event endpoint and the BIN CE are tested using a log-rank test and a χ^2 test, respectively, all of them two-sided. For MTMC, the smallest Bonferroni-adjusted two-sided p-value is reported. The overall two-sided p-value of the GPC is reported. Significant p-values (< 0.05) are bold.

BIN CE = Binary composite endpoint, D = Death, G = Graft loss, R = Rejections, I = Infections, MTMC = Multiple testing and multiplicity correction, GPC = Generalized pairwise comparisons

C Additional results investigating the impact of different data generating mechanisms including sample sizes, effect sizes, correlations and type of statistical tests

This section provides figures that extend the plots shown in the main paper. This means in this section we refer to the figures in the main paper and show for example the same figures using different sample sizes. Furthermore, Supplement section X provides the results of all simulations conducted, ordered by simulation setup, data generating mechanism or research question.

C.1 Larger group sizes and binary composite endpoint

An extension of Fig. 4 to larger group sizes is given in Supplementary Fig. S3. Additional results showing the comparison of power of a χ^2-test of a binary composite endpoint to the log-rank test of a time-to-event composite is shown in Supplementary Fig. S4. The power was very similar using a binary or time-to-event composite endpoint (using χ^2 or log-rank tests, respectively). As is known from previous research, the efficiency of tests of proportions and the log-rank test are very similar in case of relatively short and fixed follow-up times with a high number of censored observations [2, 4].

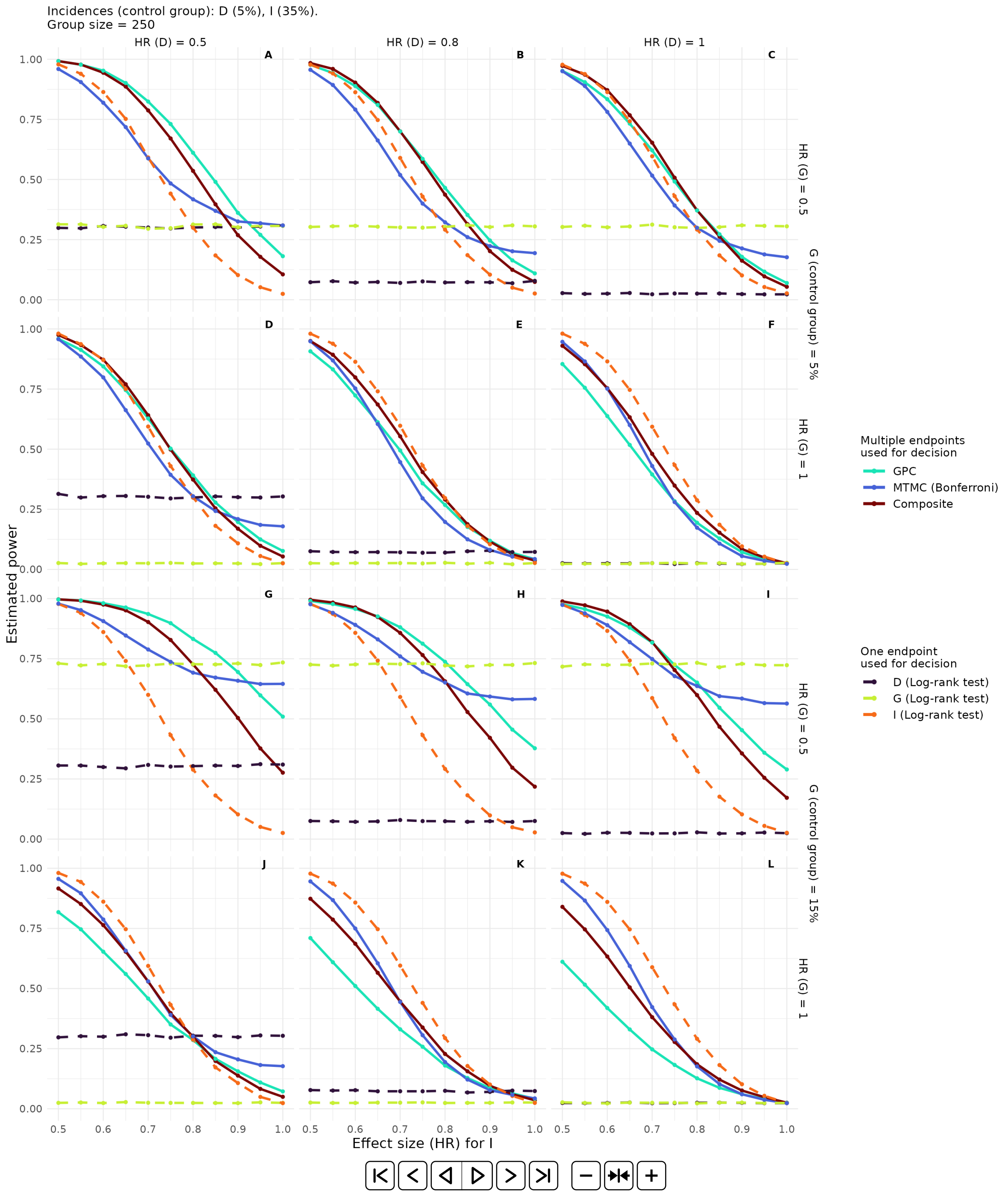

Supplementary Figure S3: ANIMATION VARYING GROUP SIZES (values shown in the figure header): Estimated power of the approaches using time-to-event endpoint definitions and one-sided tests in various scenarios including different groups sizes. The plot grids under each headline are similar to Fig. 4, only the group sizes are as indicated in the respective headline. The grids of plots show scenarios with a group size of 250, 500 and 1000 in each study arm. In the first row of each plot grid, the expected proportion of graft losses (G) in the control group amounts to 5% and a marked treatment effect on graft loss (hazard ratio (HR) = 0.5) is present. In the second row of each plot grid, the expected proportion of G is 15% and there is no treatment effect on G (HR = 1). Parameters fixed in all of the shown scenarios are the expected proportions of deaths (D) and infections (I) in the control group (5% and 35%, respectively). The x-axis of plots depicts the HR of I (0.5, large difference, to 1, i.e., equal hazards for infection in both groups). On the y-axis the estimated power is plotted. The HR of D increases within a row from left to right taking values 0.5, 0.8, and 1. Solid lines identify procedures taking all endpoints into account; Bonferroni correction (blue), Composite endpoint (darkred), GPC (turquoise). Dashed lines indicate that tests are performed on one single endpoint without multiplicity correction; one-sided log-rank of graft loss (light green), infections (orange), and death (black).

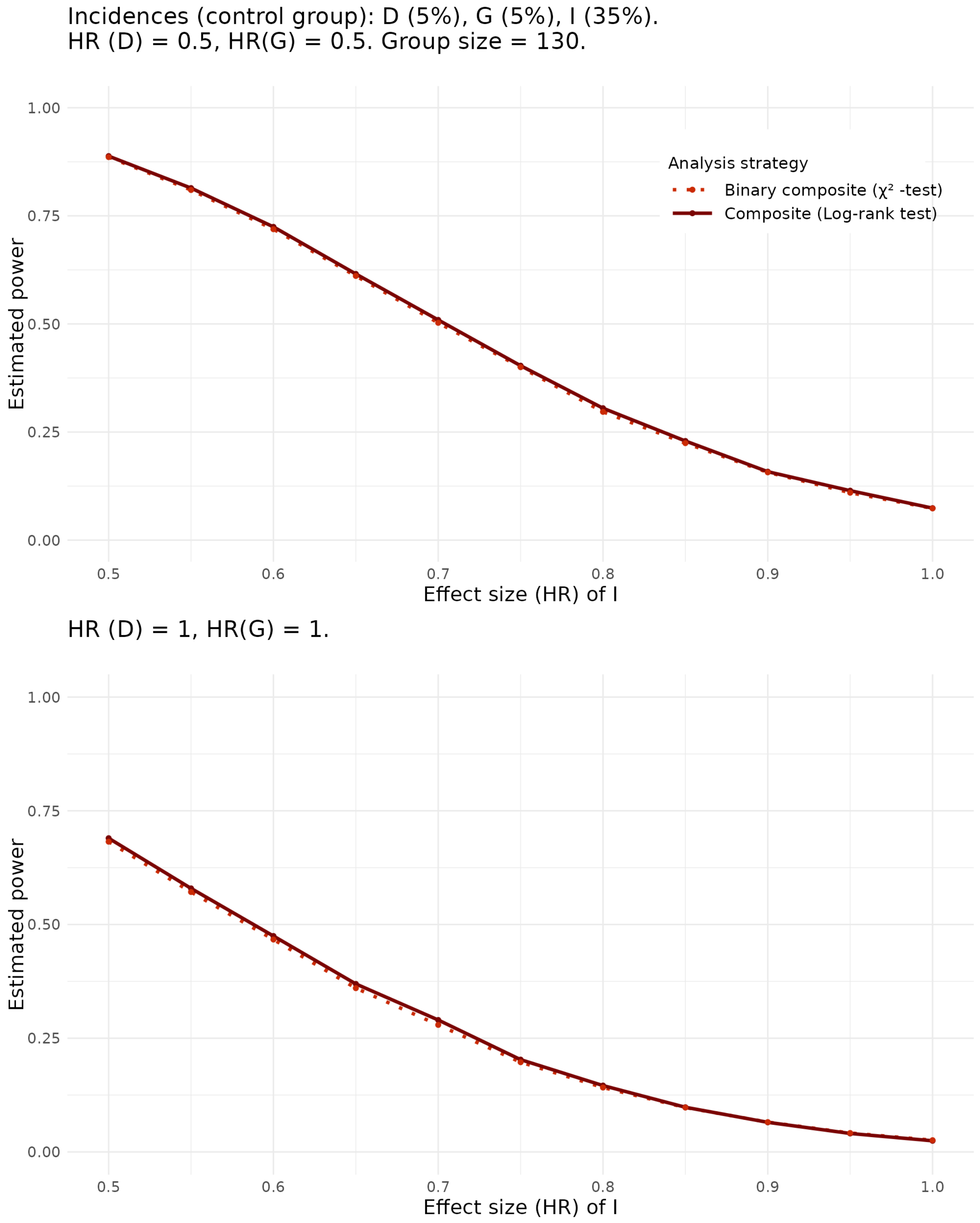

Supplementary Figure S4: Estimated power of two composite endpoint definitions both using one-sided tests in a scenario only varying effect sizes. The expected proportion of deaths (D), graft losses (G) and infections (I) in the control group is fixed to 5%, 5% and 35%, respectively, the group size to 130 participants. A marked treatment effect on G and D (hazard ratio (HR) = 0.5) is present in the upper plot. In the lower plot, treatment does not affect D and G. The x-axis of plots depicts the HR of I (0.5, a strong treatment effect, to 1, i.e., no effect, left to right). On the y-axis the estimated power is plotted. The solid line identifies the Composite endpoint defined as time-to-first-event (tested via log-rank-tests, dark red), the dotted line the binary composite endpoint (tested via χ^2-tests, red).

C.2 No terminal event

Supplementary Fig. S5 provides the power of the testing strategies with and without a terminal event like death and resulting semi-competing risk structure. Differences in power between the data generating models are subtle in most scenarios, i.e., the transparent (semi-competing risks) and non-transparent (no semi-competing risks) version of each line are almost on top of each other (one would have to zoom in to see the transparent lines). The power - of composite endpoint and GPC more than MTMC - increases in scenarios where the incidence of the first endpoint, death or A, is high (see Supplementary Fig. S15 plot grid 5).

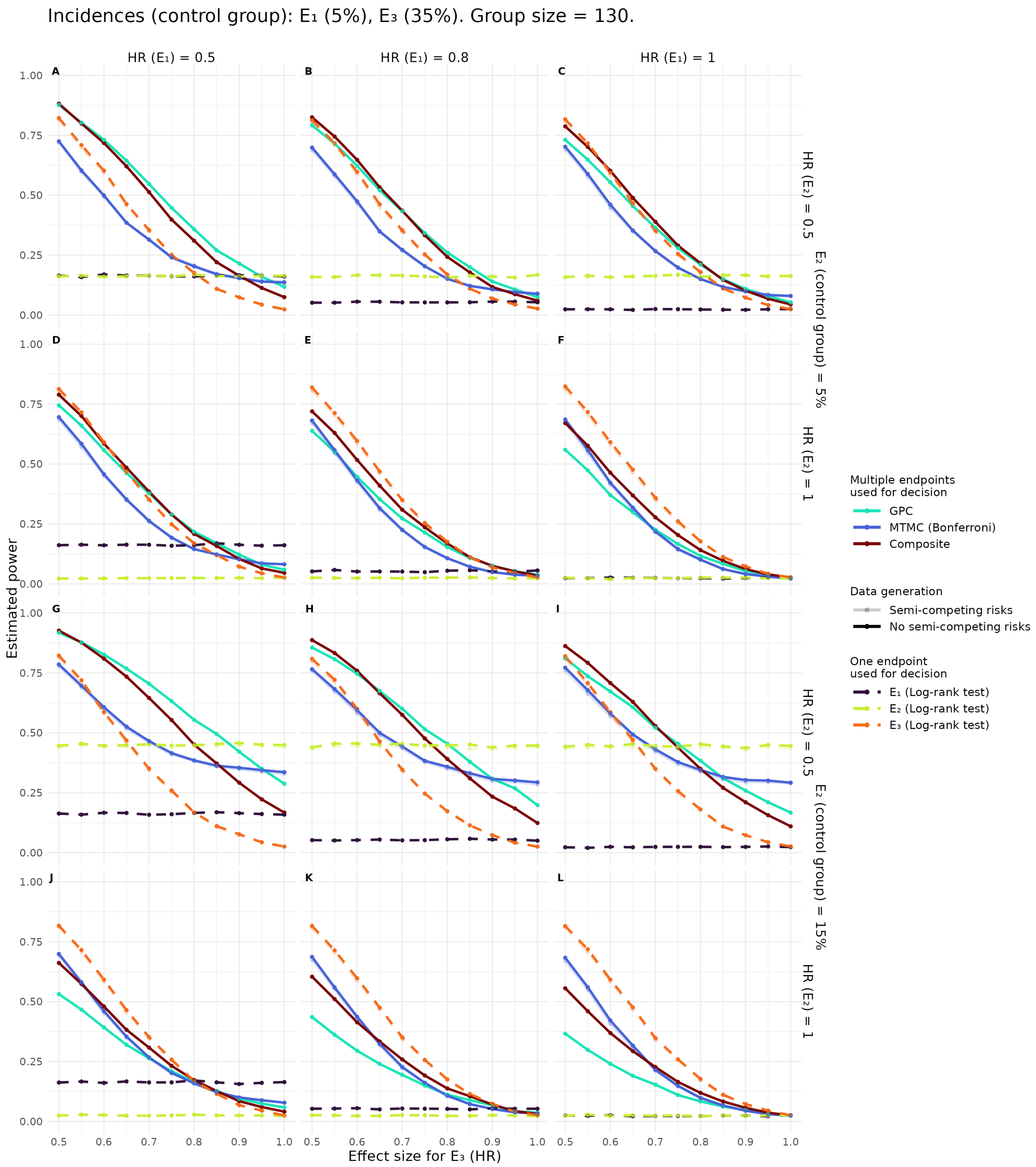

Supplementary Figure S5: Estimated power of the approaches using time-to-event endpoint definitions and one-sided tests in the scenarios from Figure 3 in the main paper. The power of tests for when there is a terminal event present ("Semi-competing risks") is depicted as transparent lines and dots in the same colour as the non-transparent power estimates for unrelated endpoints ("No semi-competing risks"). In the upper two rows of plots, the expected proportion of E_2 in the control group amounts to 5% and a marked treatment effect on E_2 (hazard ratio (HR) = 0.5, first row) and no treatment effect (HR = 1, second row) is present. In lower two rows of plots, the expected proportion of E_2 is 15% and again there is a marked and no treatment effect in the third and fourth row, respectively. Parameters fixed in all of the shown scenarios are the expected proportions of E_1 and E_3 in the control group (5% and 35%, respectively). The x-axis of plots depicts the HR of E_3. On the y-axis the estimated power is plotted. The HR of E_1 increases within a row from left to right taking values 0.5, 0.8, and 1. Solid lines identify procedures taking all endpoints into account; Bonferroni correction (blue), Composite endpoint (darkred), GPC (turquoise). Dashed lines indicate that tests are performed on one single endpoint without multiplicity correction; log-rank tests for superiority of the treatment of E_2 (dark green), E_3 (orange), and E_1 (black).

C.3 Opposing treatment effects

In scenarios with opposing treatment effects across some of the endpoints, the probability of rejecting the null hypothesis is not necessarily bounded by the nominal significance level depending on the testing strategy. This is because, depending on the testing strategy, the null hypothesis under consideration may still be false even if the treatment has a detrimental effect on one endpoint. For example, global testing procedures such as composite endpoints or generalized pairwise comparisons test the null hypothesis of no treatment effect across all components, and this null remains under the alternative as long as a beneficial effect exists for at least one clinically relevant endpoint.

Consequently, rejection of the global null may occur despite the presence of harm in another component, reflecting a fundamental limitation of hypothesis definitions that aggregate information across endpoints. This behaviour does not represent a failure of type I error control, but rather follows directly from the scientific question encoded in the null hypothesis.

Power estimates for strategies incorporating multiple endpoints in selected scenarios with opposing treatment effects are shown in Supplementary Fig. S6. As in scenarios with uniformly positive treatment effects, the power of composite endpoints—and even more so of GPC—depends more strongly on higher-ranked endpoints than does MTMC. Consequently, when opposing effects occur in more important endpoints, the power of these global strategies decreases more rapidly (see Supplementary Fig. S6, plots from left to right within each row). This pattern is particularly pronounced when a negative (opposing) treatment effect is also present for graft loss (second row of both plot grids, Supplementary Fig. S6D–F and J–L) or when the incidence of higher-ranked endpoints is increased (lower plot grid, Supplementary Fig. S6G–L).

As expected, the power curve for testing the single endpoint I remains almost identical across all panels, as it is largely unaffected by the other endpoints, with only a minor influence from the semi-competing risk of death. In contrast, GPC is most strongly affected by opposing effects in higher-ranked endpoints; for example, rejection probabilities for GPC decrease much more steeply with increasing hazard ratios in these endpoints compared with the composite strategy. In the presented scenarios, MTMC is hardly affected, as rejections are mainly driven by infections. When a component exhibits an opposing effect, its rejection probability cannot exceed the Bonferroni-adjusted significance level and therefore can only marginally contribute to the rejection probabilities.

However, if two-sided tests were used (see Supplementary Figure S7 and S8), rejection probabilities could also increase in scenarios with opposing effects. In particular, a negative treatment effect (e.g., a hazard ratio greater than 1) in one component would itself contribute evidence against the null hypothesis, because two-sided testing treats deviations in either direction as departures from the null. As a result, opposing treatment effects across endpoints may increase rejection probabilities relative to one-sided testing, even when such effects are clinically undesirable. This further highlights that rejection behaviour is driven by the definition and directionality of the tested hypothesis rather than by clinical benefit alone.

These findings underscore that any rejection of a global test in the presence of heterogeneous treatment effects must be accompanied by a careful examination of the individual component endpoints. Such post hoc or supportive analyses are essential to assess the clinical relevance of observed benefits, to identify which components drive statistical significance, and to ensure that potential safety concerns are not obscured by aggregate testing strategies. This is also suggested by regulatory guidance documents[12, 10]. Accordingly, global testing strategies should be viewed as tools for detecting overall evidence of treatment differences, not as substitutes for component-wise evaluation when treatment effects are non-uniform.

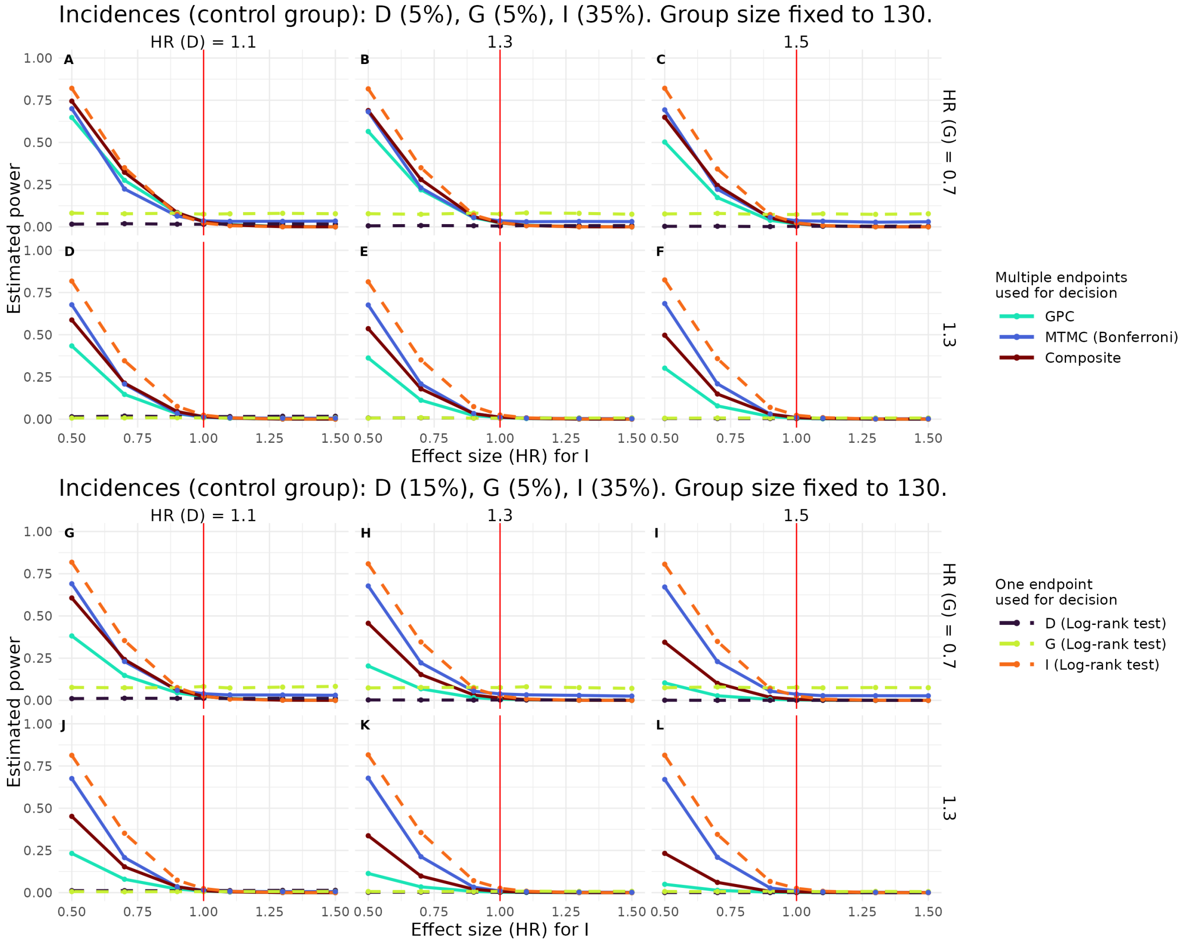

Supplementary Figure S6: Estimated power of methods using one-sided tests for superiority of the treatment in scenarios with opposing treatment effects on at least one of the endpoints. In the upper plot grid, expected incidences of the endpoints within follow-up are set to 5%, 5% and 35% for death (D), graft loss (G), and infections (I), respectively. These numbers are 15%, 5% and 35% in the lower grid of plots. The group size is fixed to 130 participants. Different hazard ratios (HRs) of death and graft loss are indicated by the facet labels in columns and rows, respectively. The HR of infections in each scenario is depicted on the x-axis. The red vertical line indicates a HR of infections of 1; left of this line, the treatment effect is positive (in favour of treatment), right of the line the opposite is the case. The y-axis gives the estimate of power of each strategy and scenario. One-sided tests for superiority of the treatment are used. Solid lines identify procedures taking all endpoints into account; Bonferroni correction (blue), Composite endpoint (darkred), GPC (turquoise). Dashed lines indicate that tests are performed on one single endpoint without multiplicity correction; log-rank tests of graft loss (dark green), infections (orange), and death (black).

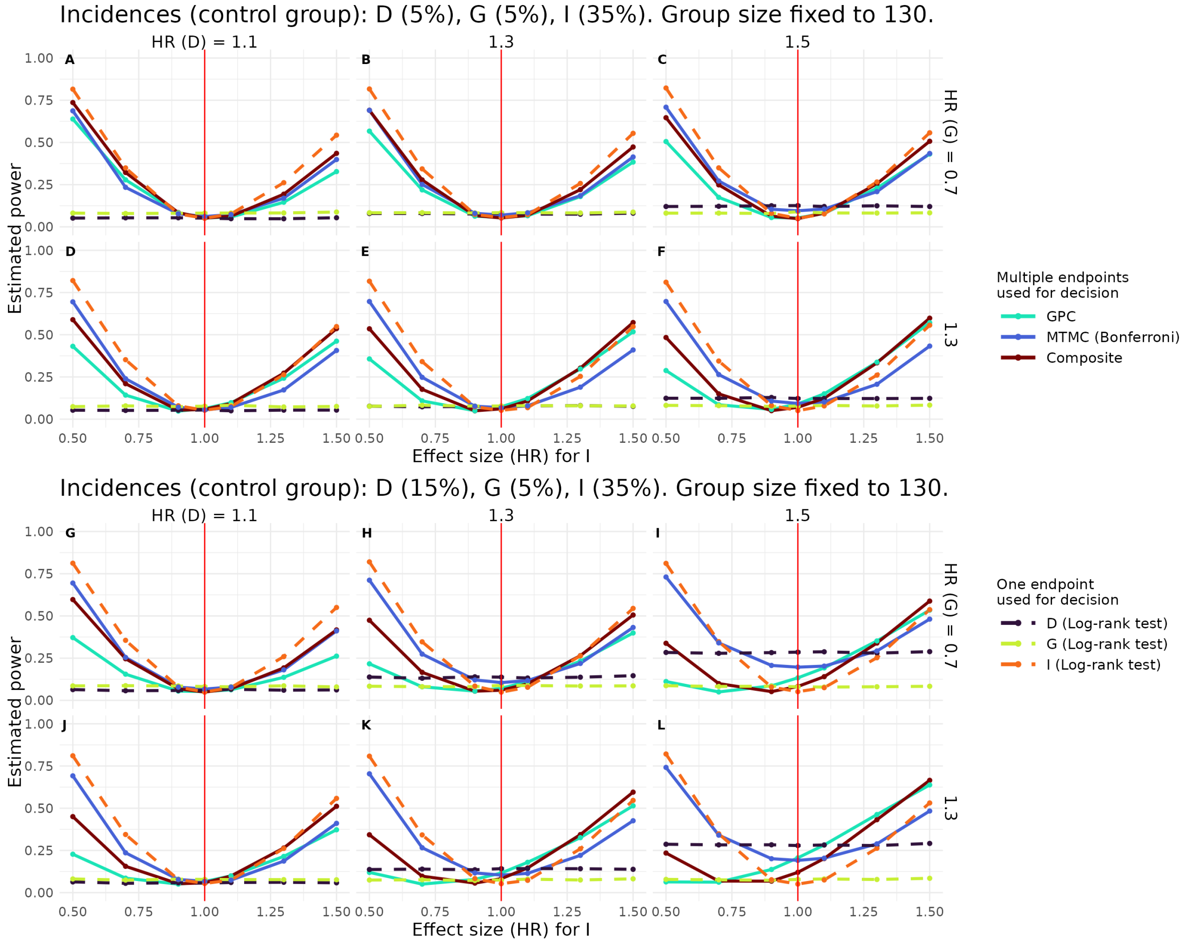

Supplementary Figure S7: Estimated power of methods using two-sided tests in scenarios with opposing treatment effects on at least one of the endpoints. In the upper plot grid, expected incidences of the endpoints within follow-up are set to 5%, 5% and 35% for death (D), graft loss (G), and infections (I), respectively. These numbers are 15%, 5% and 35% in the lower grid of plots. The group size is fixed to 130 participants. Different hazard ratios (HRs) of death and graft loss are indicated by the facet labels in columns and rows, respectively. The HR of infections in each scenario is depicted on the x-axis. The red vertical line indicates a HR of infections of 1; left of this line, the treatment effect is positive (in favour of treatment), right of the line the opposite is the case. The y-axis gives the estimate of power of each strategy and scenario. Two-sided tests for differences between treatment groups are used. Solid lines identify procedures taking all endpoints into account; Bonferroni correction (blue), Composite endpoint (darkred), GPC (turquoise). Dashed lines indicate that tests are performed on one single endpoint without multiplicity correction; log-rank tests for differences of hazards of graft loss (dark green), infections (orange), and death (black).

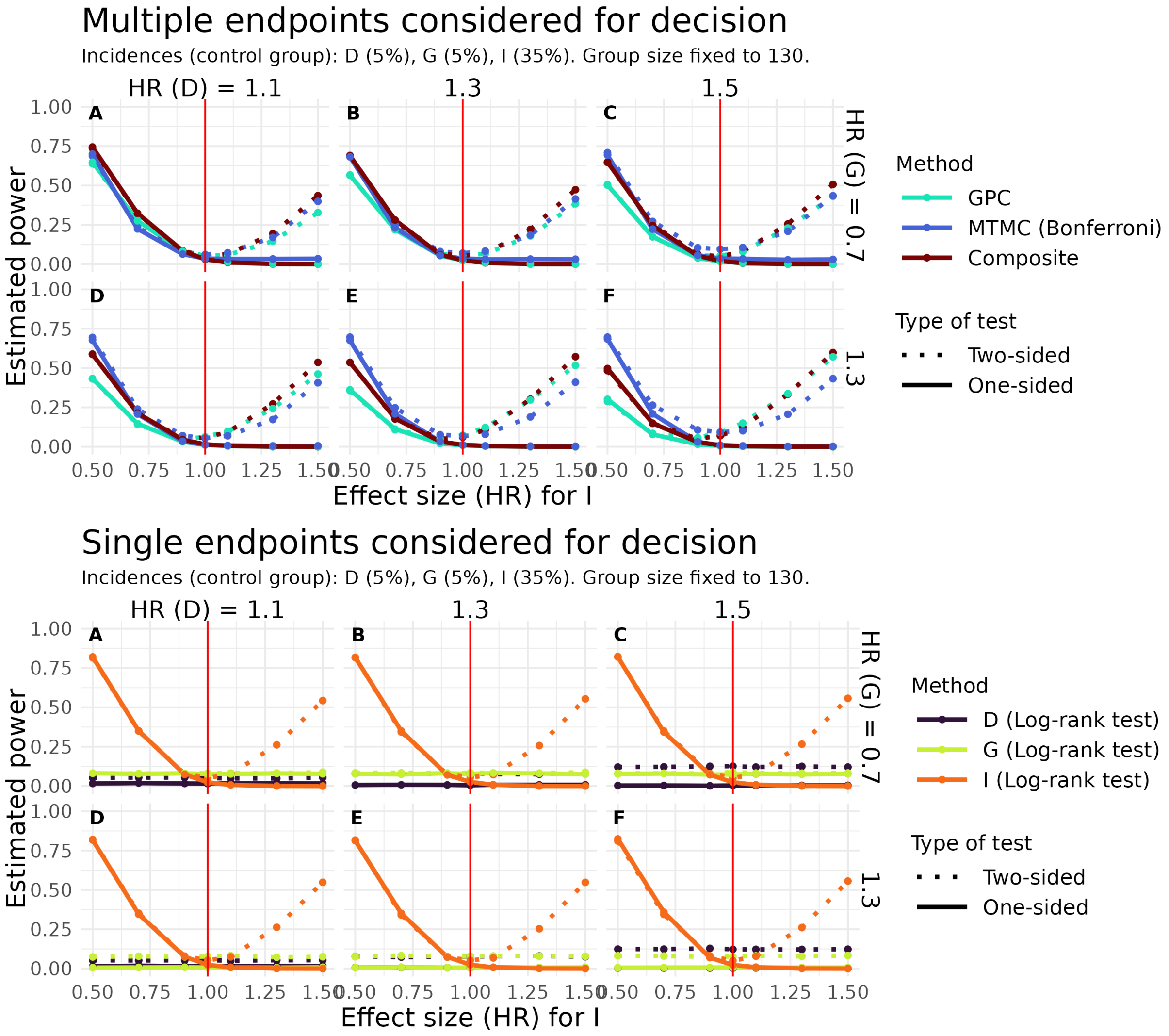

Supplementary Figure S8: Comparing the estimated power of using one- and two-sided tests in scenarios with opposing treatment effects on at least one of the endpoints. For clear display, methods that incorporate several endpoints into the analysis are depicted in the upper plot grid, and tests of single endpoints in the lower plot grids. In all plots shown, expected incidences of the endpoints within follow-up are set to 5%, 5% and 35% for death (D), graft loss (G), and infections (I), respectively, corresponding to upper plot grids of Supplementary Figures S6 and S7. The group size is fixed to 130 participants. Different hazard ratios (HRs) of death and graft loss are indicated by the facet labels in columns and rows, respectively. The HR of infections in each scenario is depicted on the x-axis. The red vertical line indicates a HR of infections of 1; left of this line, the treatment effect is positive (in favour of treatment), right of the line the opposing is the case. The y-axis gives the estimate of power of each strategy and scenario. Solid lines identify one-sided tests and dotted lines are the respective power estimates for one-sided tests. Colors mark the testing approaches; Bonferroni correction (blue), Composite endpoint (darkred), GPC (turquoise), log-rank tests of single endpoints G (dark green), I (orange), and D (black).

C.4 Additional information concerning correlated endpoints

As mentioned in the main paper, administrative censoring leads to weaker correlations between components/endpoints than expected. The extent of this discrepancy is depicted in Supplementary Fig. S9 for two different proportions of administrative censoring.

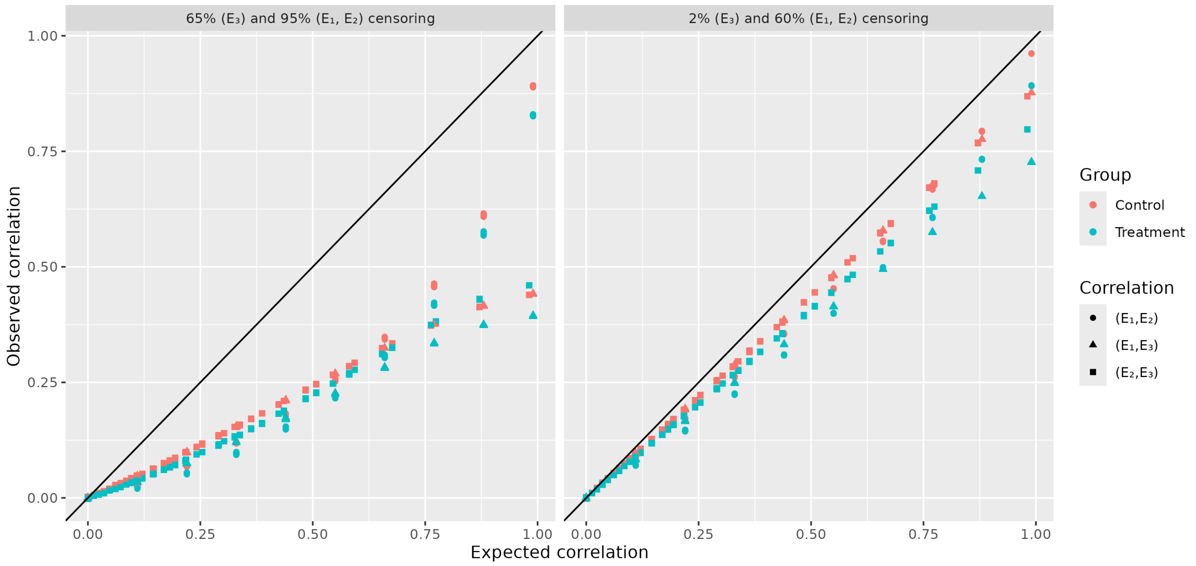

Supplementary Figure S9: Observed versus expected correlations between endpoints for two scenarios of different extent of administrative censoring. The x-axis gives the expected Spearman correlation set for the endpoint pairs. The y-axis gives the Spearman correlations actually observed. In the left plot, the proportion of patients censored are 65% regarding the third endpoint and 95% regarding first and second. In the right plot the proportion of censoring is much lower: 60%, 60% and 2% are administratively censored for the first, second and third endpoint, respectively. The dot shape indicates the pair of endpoints for which the correlation was set and observed.

In the following Supplementary Fig. S10, combinations are shown of correlations where Spearman's ρ between E_1 and E_2 is not fixed to be zero in our base scenario (i.e., incidences of E_1 and E_2 are 5% within follow-up, the third endpoint's incidence is 35%. HRs of E_2 and E_3 are fixed to 0.7 and 0.6, respectively. As can be seen in the first row of plots in Supplementary Fig. S10, the correlation between E_1 and E_2 affects the power of the GPC more than MTMC or composite and is noticeable for high correlations. For higher rates of administrative censoring, the effect is very small. Note that as the correlation between the latter two endpoints, $\rho(E_2, E_3)$, is restricted by $\rho(E_1, E_2)$ and $\rho(E_1, E_3)$, $\rho(E_2, E_3)$ also increases within a plot from left to right (indicated by labels in each scenario in Supplementary Fig. S10). The isolated effect of $\rho(E_1, E_2)$ is thus indistinguishable from the effect of varying both, $\rho(E_1, E_2)$ and $\rho(E_2, E_3)$.

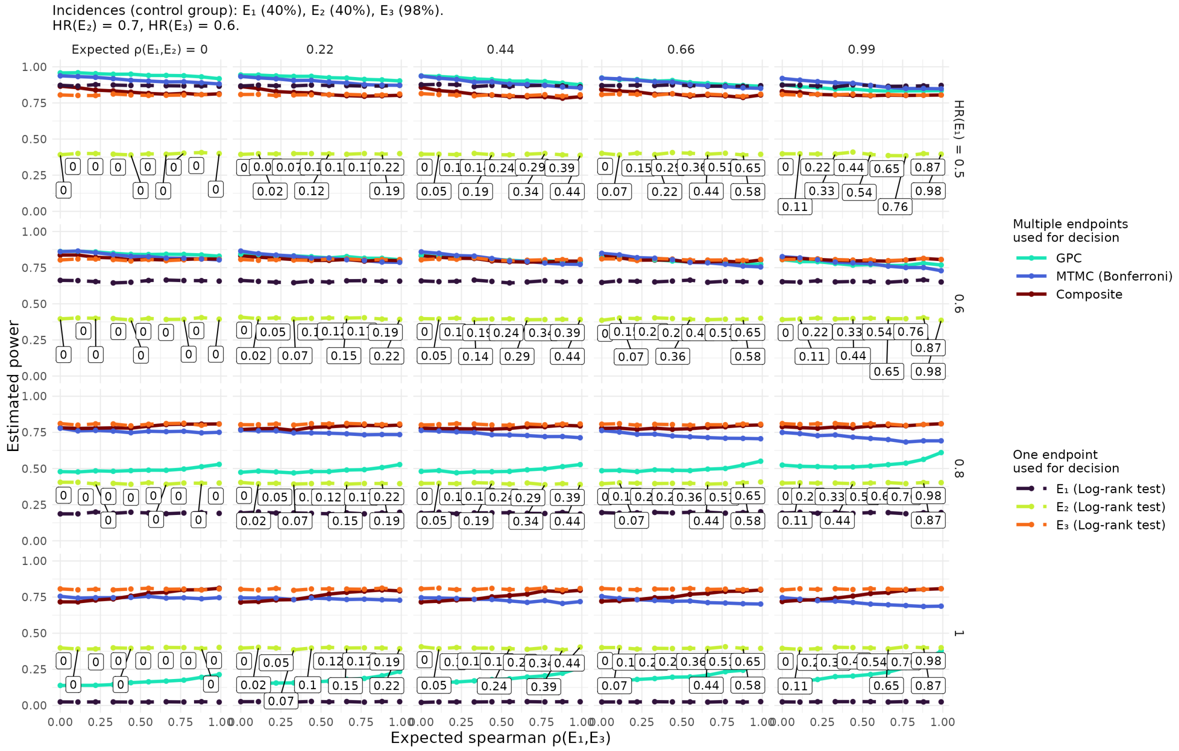

Supplementary Figure S10: Estimated power of the analysis strategies in scenarios with correlations between endpoints. The Spearman correlation between endpoints E_1 and E_2, $\rho(E_1, E_2)$ is set to zero. The group size is fixed to 130 participants. In the upper row of plots, the expected proportion of participants in the control group reaching the endpoint E_1 and E_2 is 5%, and 35% for endpoint E_3. In the lower row of plots, the expected incidence is 15% for E_1. Hazard ratios of E_2 and E_3 are 0.7 and 0.6, respectively, in all plots. The effect size for E_1 decreases within a row from left to right and is given in the facet labels. The x-axis gives the set Spearman correlations between endpoints E_1 and E_3, $\rho(E_1, E_3)$. The y-axis gives the estimate of power of each strategy and scenario. One-sided tests for superiority of the treatment are used. Solid lines identify procedures taking all endpoints into account; Bonferroni correction (blue), Composite endpoint (darkred), GPC (turquoise). Dashed lines indicate that tests are performed on one single endpoint without multiplicity correction; one-sided log-rank tests of E_1 (black), E_2 (light green), and E_3 (orange).

D One-sided (superiority) versus two-sided (difference) testing

Both tests for differences between groups (two-sided) and testing superiority of treatment (one-sided) can be used in clinical trials. Usually, though, the question of interest is superiority of treatment only and superiority of the control group would not be declared a success of the trial. We therefore use tests for superiority of the treatment group. Supplementary Table S1 gives an overview over hypotheses used for testing differences between arms (two-sided tests) and superiority of the treatment (one-sided tests).

Supplementary Fig. S11 compares the power of the testing strategies as tests for differences between treatment groups in the same scenarios shown in the main paper Figure 3. The power is very similar and only differs notably if the power approaches the type-I-error rate (towards the right hand side of plots in Supplementary Fig. S11 that is 2.5% for tests of superiority as opposed to 5% for tests of differences.

Similarly, Supplementary Fig. S12 is the equivalent of Figure 4 in the main paper but using two-sided tests for differences. We did not investigate two-sided tests in scenarios including correlated endpoints.

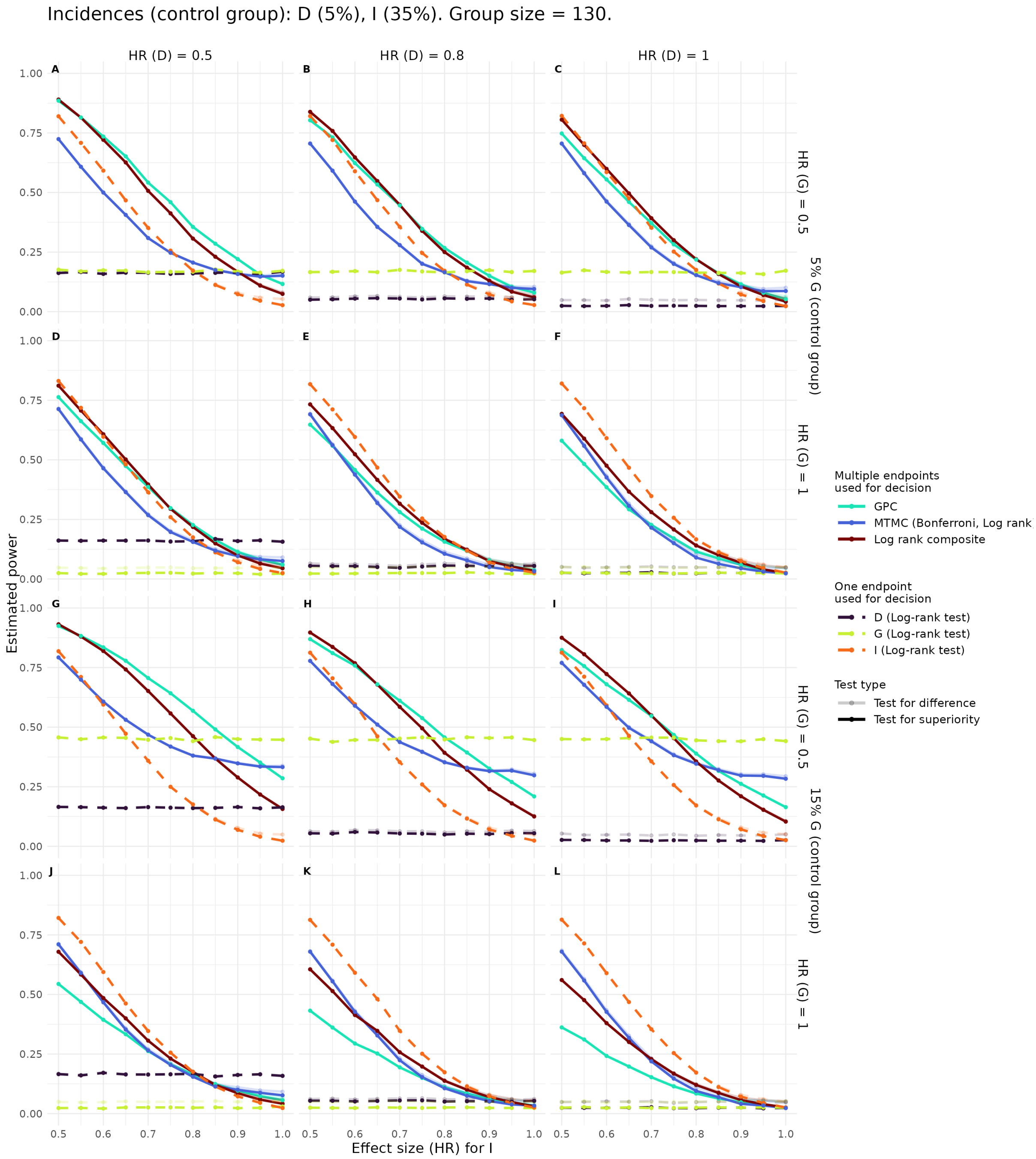

Supplementary Figure S11: Estimated power of the approaches using time-to-event endpoint definitions in the scenarios from Figure 3 in the main paper. The power of tests for differences between treatment groups are given as transparent lines and dots in the same colours as the tests for superiority of the treatment. In the first row of plots, the expected proportion of graft losses in the control group amounts to 5% and a marked treatment effect on graft loss (hazard ratio (HR) = 0.5) is present. In the second row of plots, the expected proportion of graft losses is 15% and there is no treatment effect on graft loss (HR = 1). Parameters fixed in all of the shown scenarios are the expected proportions of deaths and infections in the control group (5% and 35%, respectively). The x-axis of plots depicts the HR of infections. On the y-axis the estimated power is plotted. The HR of death increases within a row from left to right taking values 0.5, 0.8, and 1. Solid lines identify procedures taking all endpoints into account; Bonferroni correction (blue), Composite endpoint (darkred), GPC (turquoise). Dashed lines indicate that tests are performed on one single endpoint without multiplicity correction; log-rank tests of graft loss (dark green), infections (orange), and death (black).

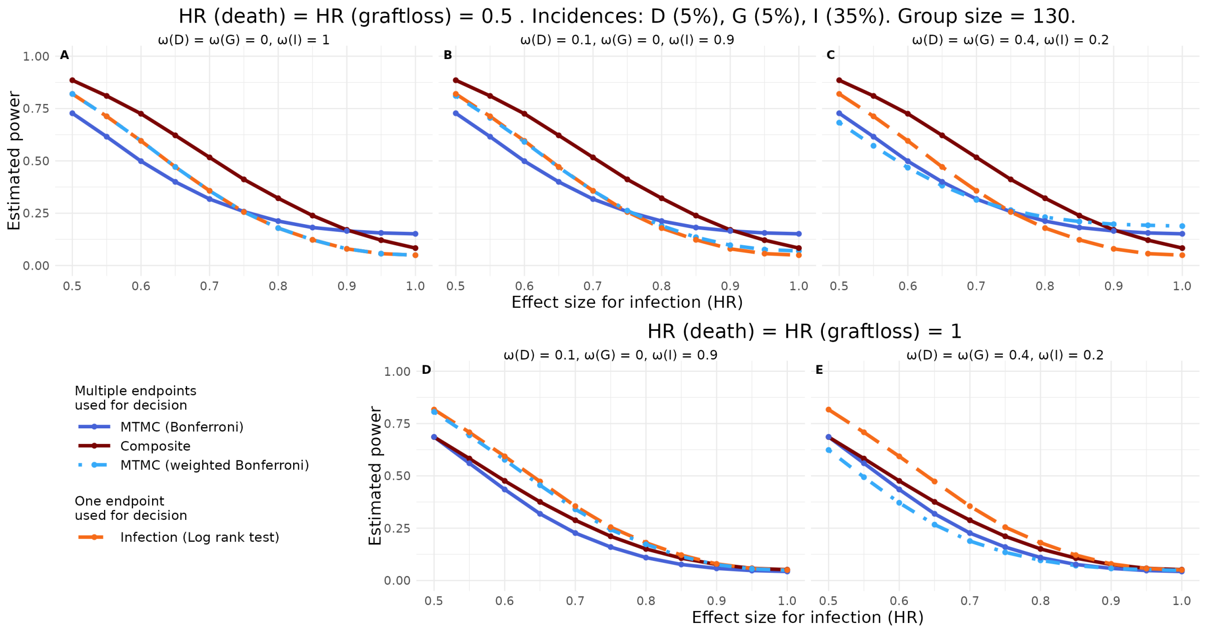

Supplementary Figure S12: Comparison of estimated power of a weighted Bonferroni procedure and aforementioned approaches using two-sided tests in various scenarios. Time-to-event endpoint definitions are again used and differences between groups tested by log-rank tests. The expected proportion of deaths, graft losses and infections in the control group is fixed to 5%, 5% and 35%, respectively, in all shown plots. In the first row of plots, a marked treatment effect on graft loss and death (hazard ratio (HR) = 0.5) is present. In the lower plot, there is no treatment effect on graft loss nor death (HR = 1). The x-axis of plots depicts the HR of infections (0.5, a strong treatment effect, to 1, i.e., no effect). On the y-axis the estimated power is plotted. The weights, ω, applied to adjust the tests p-values varies and is given above each plot. Solid lines identify procedures taking all endpoints into account; Multiple testing and multiplicity correction (MTMC) using log-rank tests and Bonferroni correction (blue), Composite endpoint (darkred). Dashed lines indicate log-rank tests of infections (orange). Dashed-dotted lines are the weighted version of the Bonferroni correction (light blue).

E Overview of results of all investigated scenarios

E.1 Plots of all scenarios evaluated (one-sided tests)

The following slide shows show plots for all scenarios evaluated using tests for superiority of the treatment. For some of the plots, additional HRs of infections were simulated to provide smoother plots. These are not included for all plot grids but would be seen as more granular measurements in the plots shown. The comparisons of strategies on time-to-event endpoint definitions is shown in Supplementary Fig. S13. Contrasting weighted and unweighted Bonferroni multiplicity correction in all investigated scenarios is shown in Supplementary Fig. S14. All scenarios that were investigated to compare the power when there is a semi-competing risk like death to the power when looking at unrelated endpoints is given in Supplementary Fig. S15. For simulations with correlated endpoints, all the results of scenarios keeping Spearman correlations between the first and second component (e.g. E_1 and E_2), fixed at zero - i.e., $\rho(E_1, E_2) = 0$ - are shown in Supplementary Fig. S16-S18. This is only an excerpt and simulations for $\rho(E_1, E_2) \neq 0$ were performed as well. An example of these results in one scenario can be seen in Supplementary Fig. S10. Results of scenarios including opposing treatment effects are depicted Supplementary Fig. S19. In all of the above scenarios, the group size is always fixed to 130 participants per group. The results of simulations with larger group sizes are shown in Supplementary Fig. S20-S23.

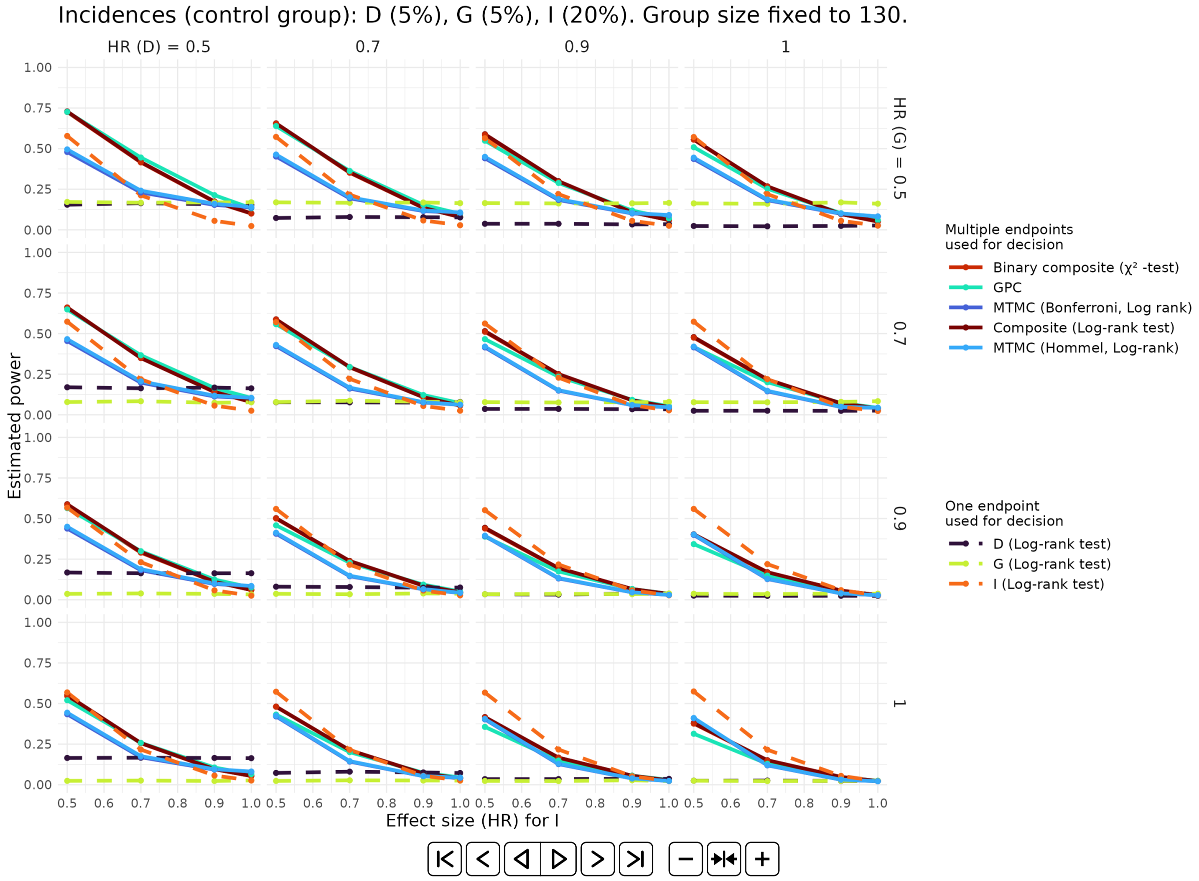

Supplementary Figure S13: ANIMATION VARYING INCIDENCES (values shown in the header of the figure): Estimated power of the approaches using time-to-event endpoint definitions and one-sided tests in various scenarios. The titles indicate the parameters not explicitly shown in the plots. These are the expected proportions of deaths (D), graft loss (G) and infections (I) in the control group. The group size is fixed to 130 participants. The x-axis of plots depicts the HR of infections (0.5, large difference, to 1, i.e., equal hazards for infection in both groups). On the y-axis the estimated power is plotted. The facet labels of each column give the hazard ratio (HR) of death, the rows the HR of graft loss. The HR of death increases within a row from left to right taking values 0.5, 0.7, 0.9, and 1 and likewise for graft loss in columns. Solid lines identify procedures taking all endpoints into account; Bonferroni correction (blue), Composite endpoint (darkred), GPC (turquoise). Dashed lines indicate that tests are performed on one single endpoint without multiplicity correction; one-sided log-rank tests of graft loss (light green), infections (orange), and death (black).

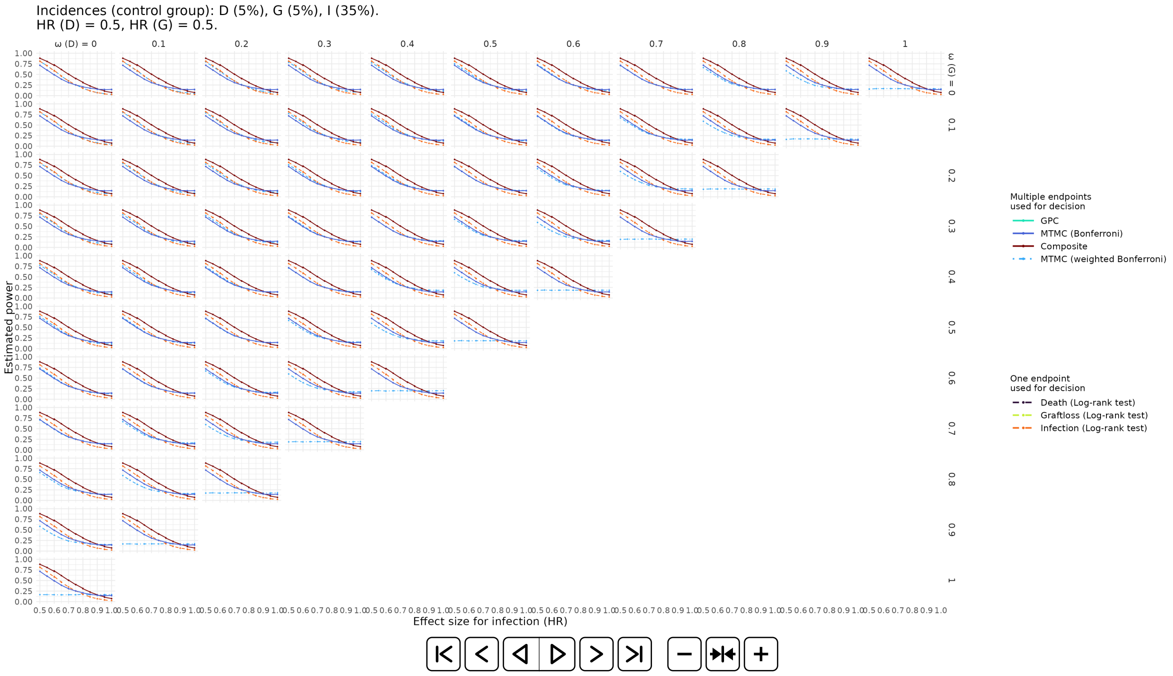

Supplementary Figure S14: ANIMATION VARYING HAZARD RATIOS OF DEATH AND GRAFT LOSS (values shown in the figure header)Comparison of estimated power of a weighted Bonferroni procedure and aforementioned approaches in various scenarios. Time-to-event endpoint definitions are again used and superiority of the treatment is tested by one-sided log-rank tests. The expected proportion of deaths (D), graft losses (G) and infections (I) in the control group is fixed to 5%, 5% and 35%, respectively, in all shown plots. Hazard ratios (HR) of D and G vary in each plot and are given in the title of each figure. The x-axis of plots depicts the HR of infections (0.5, a strong treatment effect, to 1, i.e., no effect). On the y-axis the estimated power is plotted. The weights, ω, applied to adjust the tests varies and is given in the column (weight of the test of D) and row (weight of the test of G) facet labels. Solid lines identify procedures taking all endpoints into account; Multiple testing and multiplicity correction (MTMC) using log-rank tests and Bonferroni correction (blue), Composite endpoint (darkred). Dashed lines indicate log-rank tests of infections (orange). Dashed-dotted lines are the weighted version of the Bonferroni correction (blue). Note that as the weights must sum to one, the weight of the tests of I follow from the other two weights. For the same reason, combinations of weights in the right-lower triangle do not exist and the plots remain empty.

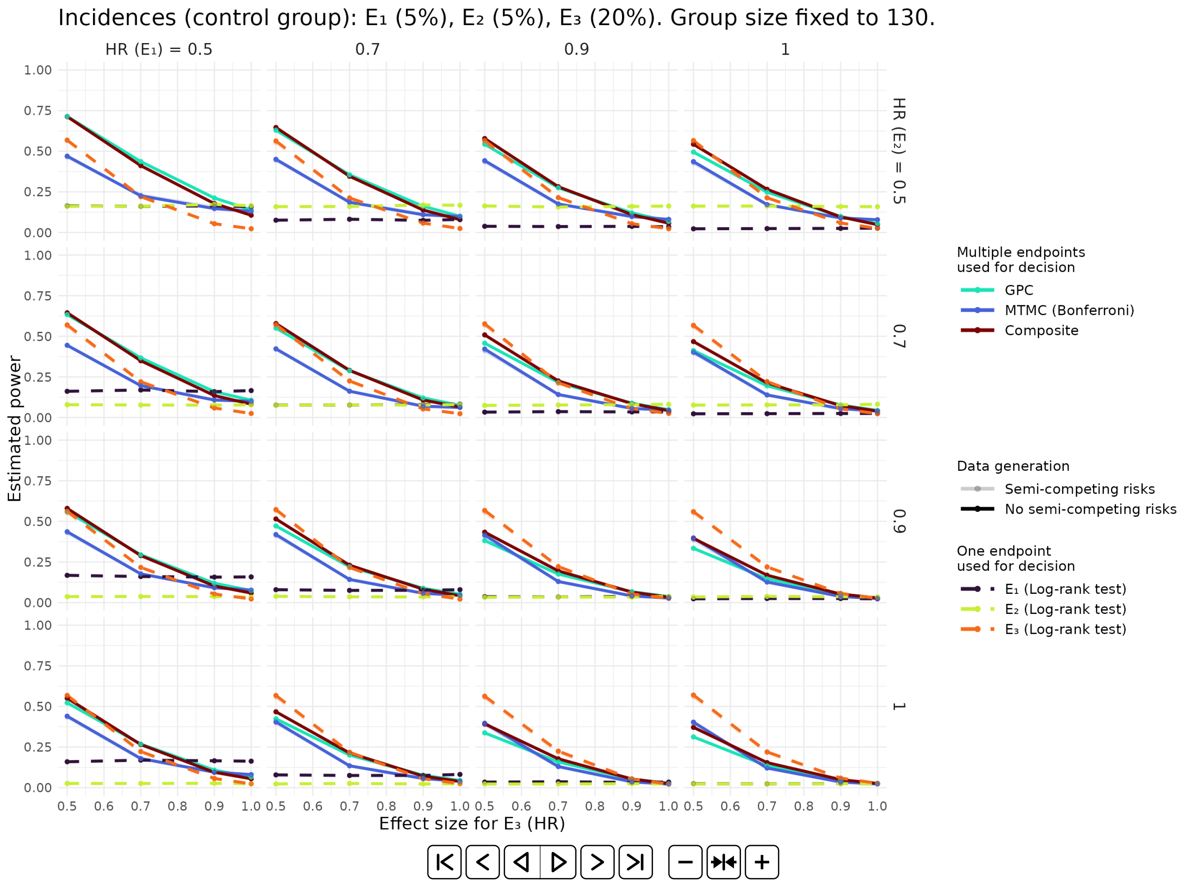

Supplementary Figure S15: ANIMATION VARYING INCIDENCES (values shown in the figure header)Estimated power of the approaches using time-to-event endpoint definitions and one-sided tests in various scenarios. Transparent lines are power estimates when there is a semi-competing risk structure, non-transparent lines are power estimates for unrelated endpoints. The titles indicate the parameters not explicitly shown in the plots. These are the expected proportions of three endpoints death (D), graft loss (G), and infections (I) in the control group. The group size is fixed to 130 participants. The x-axis of plots depicts the HR of infections. On the y-axis the estimated power is plotted. The facet labels of each column give the hazard ratio (HR) of death, increasing within a row from left to right taking values 0.5, 0.7, 0.9, and 1 and likewise for graft loss in columns. Solid lines identify procedures taking all endpoints into account; Bonferroni correction (blue), Composite endpoint (darkred), GPC (turquoise). Dashed lines indicate that tests are performed on one single endpoint without multiplicity correction; one-sided log-rank tests of graft loss (light green), infections (orange), and death (black). Note that for comparison with the original data generating mechanism, the names of endpoints are kept although the observations of events after the first endpoint would not allow for such labels.

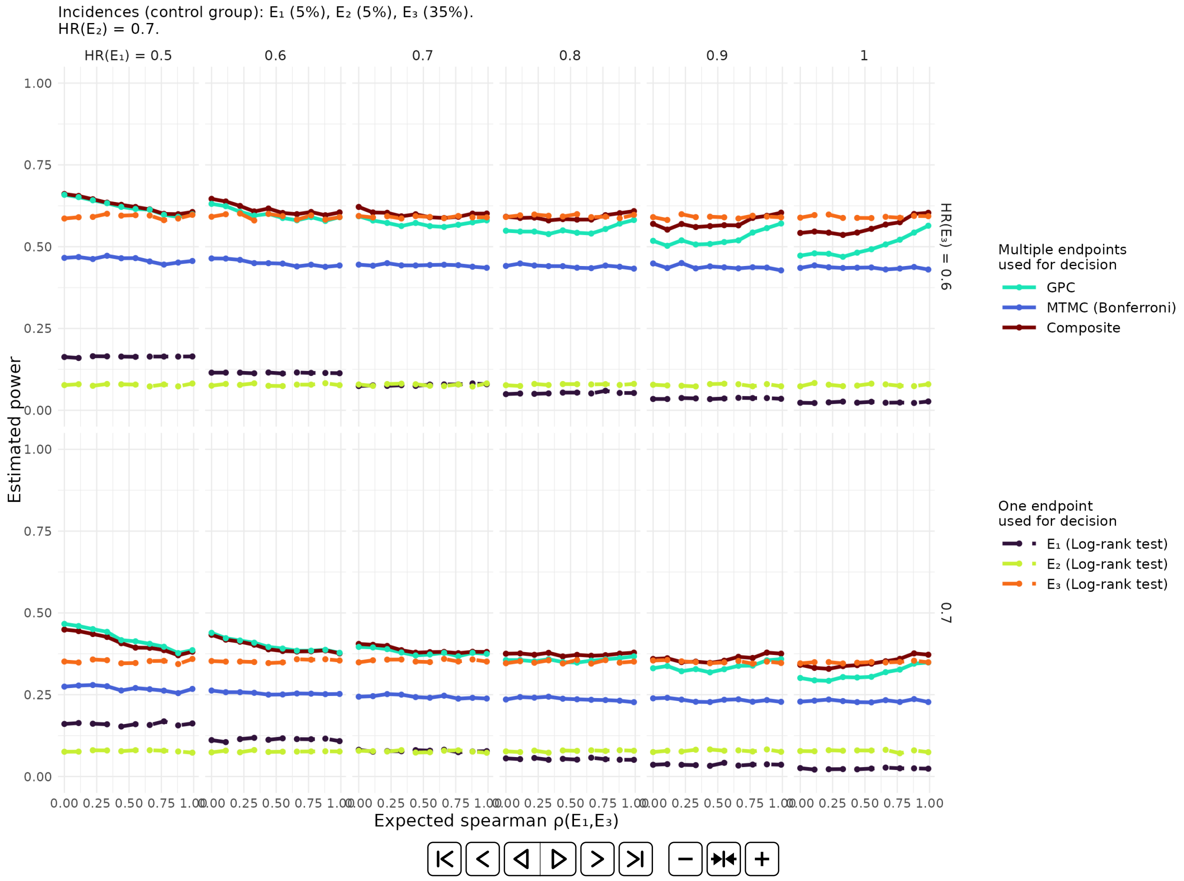

Supplementary Figure S16: ANIMATION VARYING THE INCIDENCE OF E_1 (values shown in the figure header). Estimated power of the approaches using time-to-event endpoint definitions and one-sided tests in scenarios with correlated endpoints. The titles indicate the parameters not explicitly shown in the plots. These are the expected incidences within follow-up of three endpoints E_1, E_2, and E_3 in the control group. The group size is fixed to 130 participants. The x-axis of plots depicts the expected Spearman correlation between endpoint E_1 and E_3. On the y-axis the estimated power is plotted. The facet labels of each column give the hazard ratio (HR) of E_1, increasing within a row from left to right and likewise for E_3 labels in rows. One-sided tests are used to test for superiority of the treatment. Solid lines identify procedures taking all endpoints into account; Bonferroni correction (blue), Composite endpoint (darkred), GPC (turquoise). Dashed lines indicate that tests are performed on one single endpoint without multiplicity correction; one-sided log-rank tests of E_2 (light green), E_3 (orange), and E_1 (black).

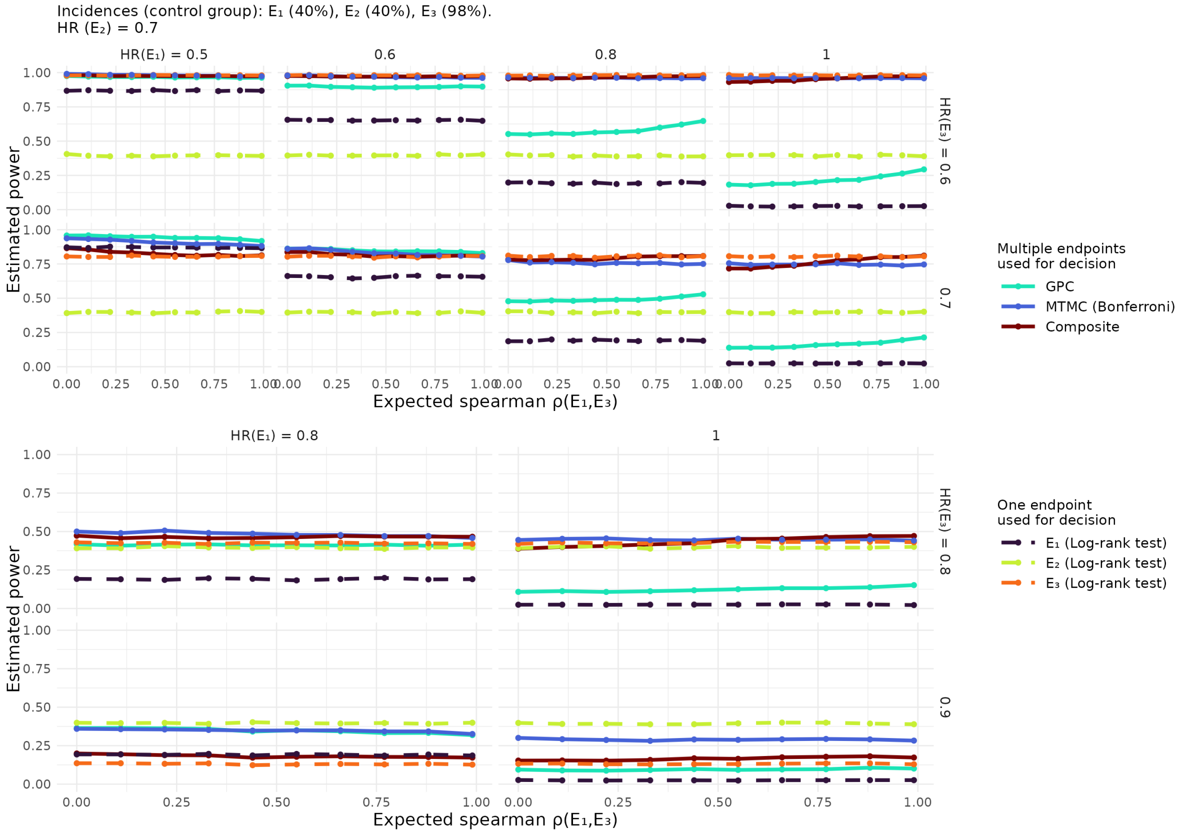

Supplementary Figure S17: Estimated power of the approaches using time-to-event endpoint definitions and one-sided tests in scenarios with correlated endpoints. The expected incidences within follow-up of three endpoints E_1, E_2, and E_3 in the control group are 40%, 40% and 98%, respectively. The group size is fixed to 130 participants. The x-axis of plots depicts the expected Spearman correlation between endpoint E_1 and E_3. On the y-axis the estimated power is plotted. The facet labels of each column give the hazard ratio (HR) of E_1, increasing within a row from left to right and likewise for E_3 labels in rows. The HR of E_2 is set to 0.7. One-sided tests are used to test for superiority of the treatment. Solid lines identify procedures taking all endpoints into account; Bonferroni correction (blue), Composite endpoint (darkred), GPC (turquoise). Dashed lines indicate that tests are performed on one single endpoint without multiplicity correction; one-sided log-rank tests of E_2 (light green), E_3 (orange), and E_1 (black). Not all combinations of HRs E_1 and E_2 were investigated and the respective plots remain empty.

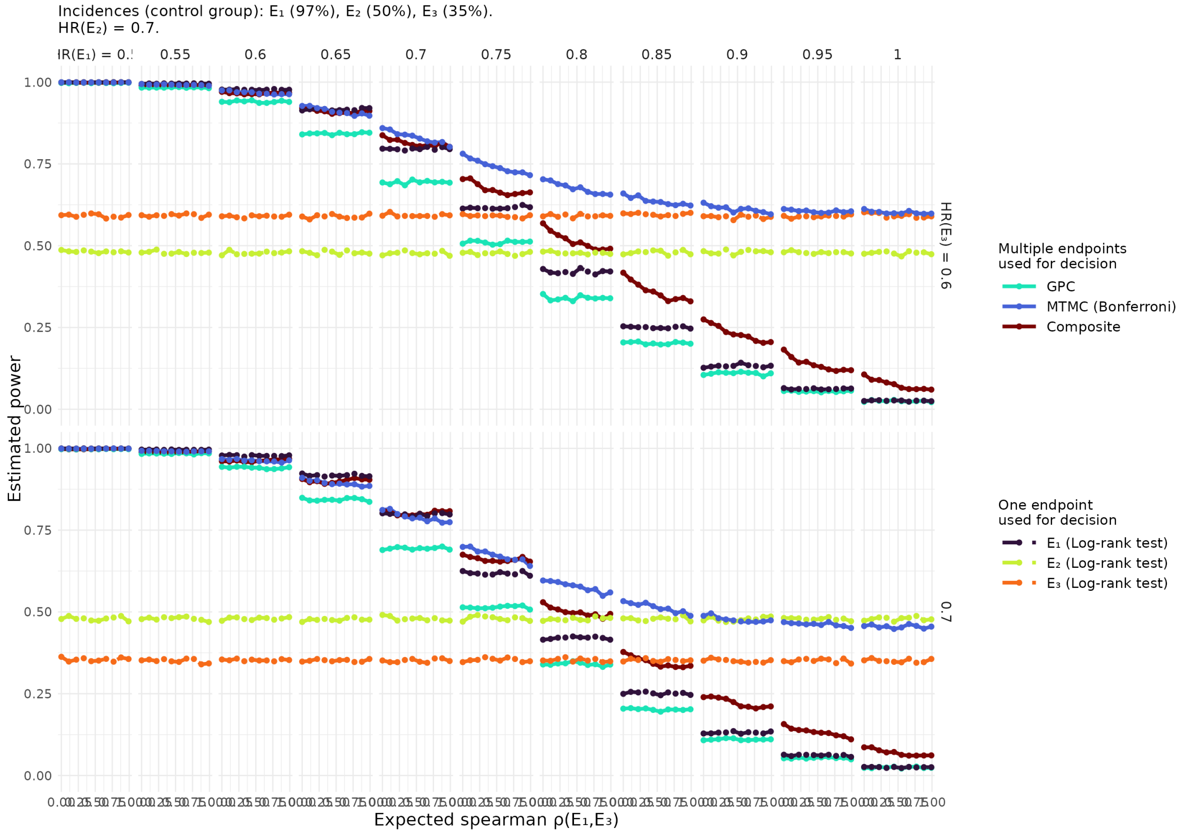

Supplementary Figure S18: Estimated power of the approaches using time-to-event endpoint definitions and one-sided tests in scenarios with correlated endpoints. The expected incidences within follow-up of three endpoints E_1, E_2, and E_3 in the control group are 97%, 50% and 35%, respectively. The group size is fixed to 130 participants. The x-axis of plots depicts the expected Spearman correlation between endpoint E_1 and E_3. On the y-axis the estimated power is plotted. The facet labels of each column give the hazard ratio (HR) of E_1, increasing within a row from left to right and likewise for E_3 in columns. The HR of E_2 is set to 0.7. One-sided tests are used to test for superiority of the treatment. Solid lines identify procedures taking all endpoints into account; Bonferroni correction (blue), Composite endpoint (darkred), GPC (turquoise). Dashed lines indicate that tests are performed on one single endpoint without multiplicity correction; one-sided log-rank tests of E_2 (light green), E_3 (orange), and E_1 (black).

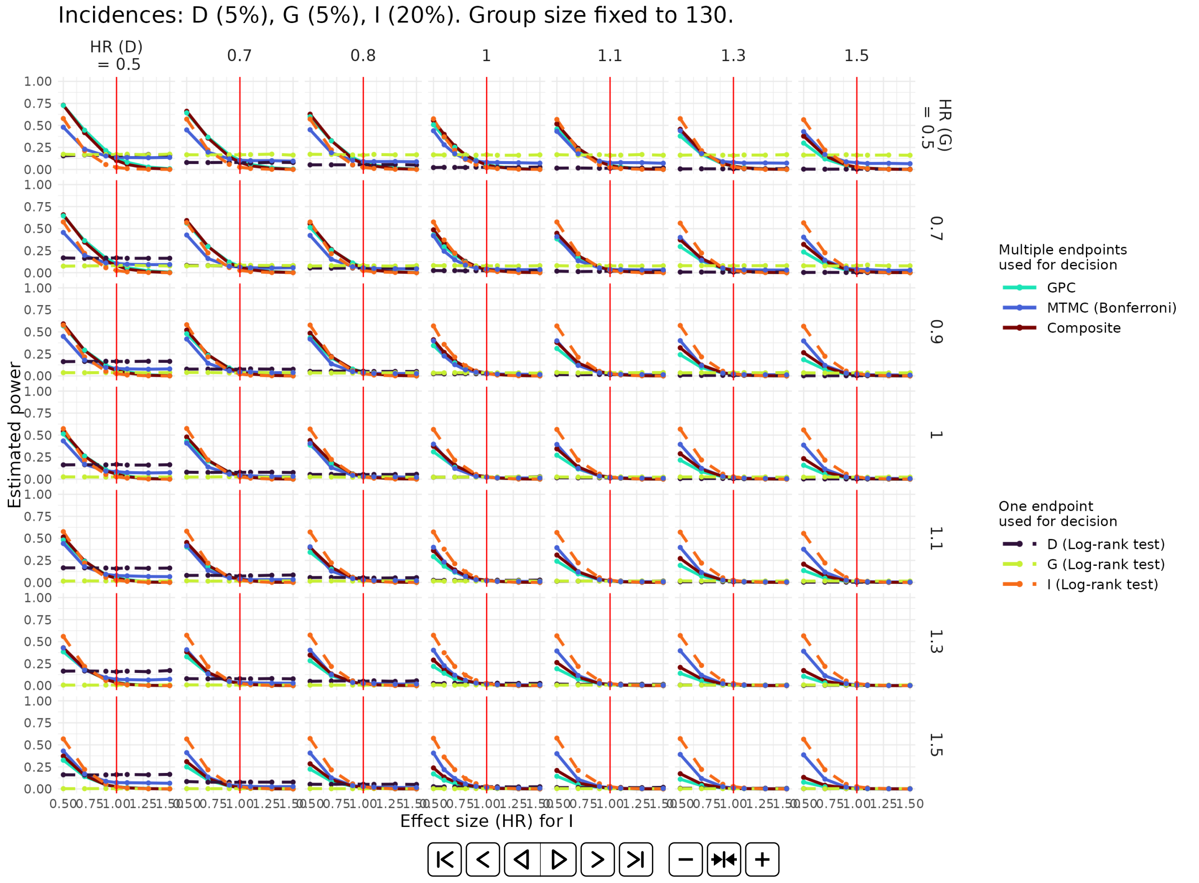

Supplementary Figure S19: ANIMATION VARYING INCIDENCES (values shown in the figure header)Estimated power of the approaches using time-to-event endpoint definitions and one-sided tests in scenarios with opposing treatment effects. The titles indicate the parameters not explicitly shown in the plots. These are the expected proportions of three endpoints death (D), graft loss (G), and infections (I) in the control group. The group size is fixed to 130 participants. The x-axis of plots depicts the HR of infections from 0.5 to 1.5. The vertical red line marks a HR of infections of 1. On the y-axis the estimated power is plotted. The facet labels of each column give the hazard ratio (HR) of death, increasing within a row from left to right taking values from 0.5, to 1.5 and likewise for HRs of graft loss in each column. Solid lines identify procedures taking all endpoints into account; Bonferroni correction (blue), Composite endpoint (darkred), GPC (turquoise). Dashed lines indicate that tests are performed on one single endpoint without multiplicity correction; one-sided log-rank tests of graft loss (light green), infections (orange), and death (black).

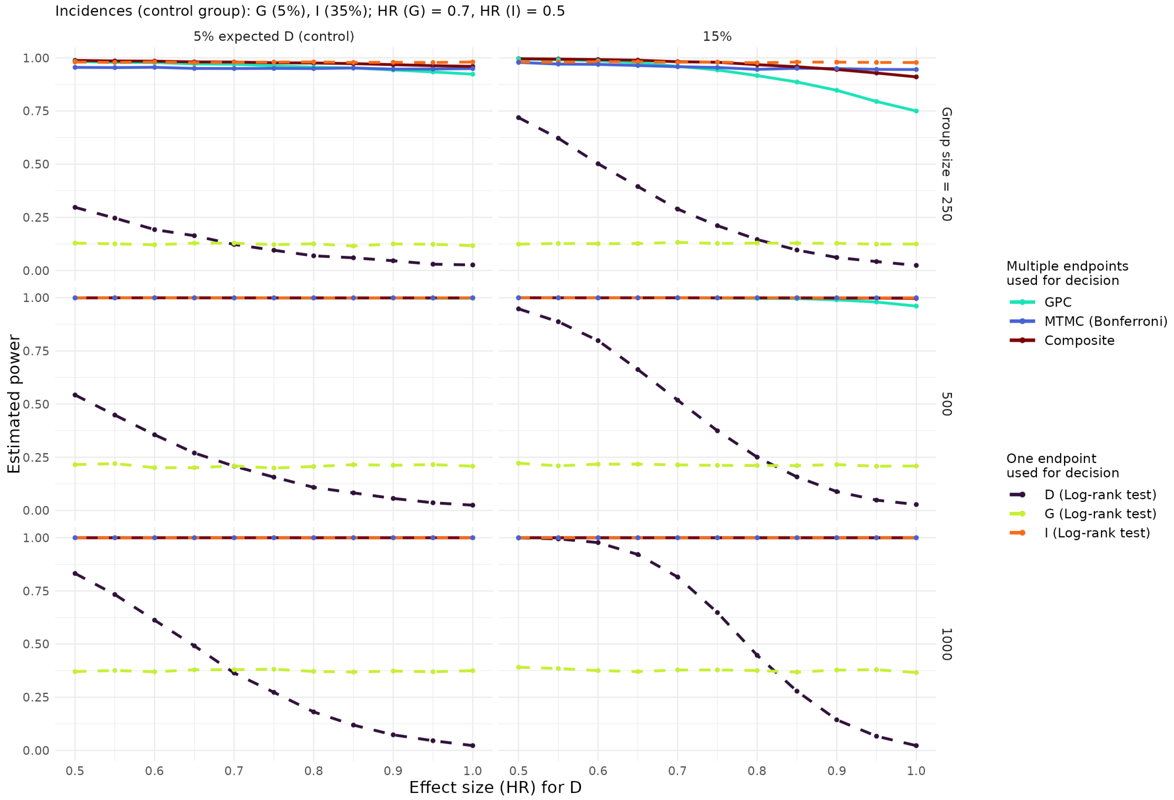

Supplementary Figure S20: Estimated power of the approaches using time-to-event endpoint definitions and one-sided tests in various scenarios including different group sizes. The titles list the parameters not explicitly shown in the plots. Incidences in the title mean the expected proportion of observed deaths (D), graft loss (G) and infections (I) in the control group. The x-axis depicts the hazard ratio (HR) of death (0.5, large difference, to 1, i.e., equal hazards for infection in both groups). On the y-axis the estimated power is plotted. The left column of plots are scenarios with incidences of death of 5%, in the right column the incidence of death is 15%. The group sizes increase by rows from 250 to 500 and 1000 participants per group. Solid lines identify procedures taking all endpoints into account; Bonferroni correction (blue), Composite endpoint (darkred), GPC (turquoise). Dashed lines indicate that tests are performed on one single endpoint without multiplicity correction; one-sided log-rank tests of graft loss (light green), infections (orange), and death (black).

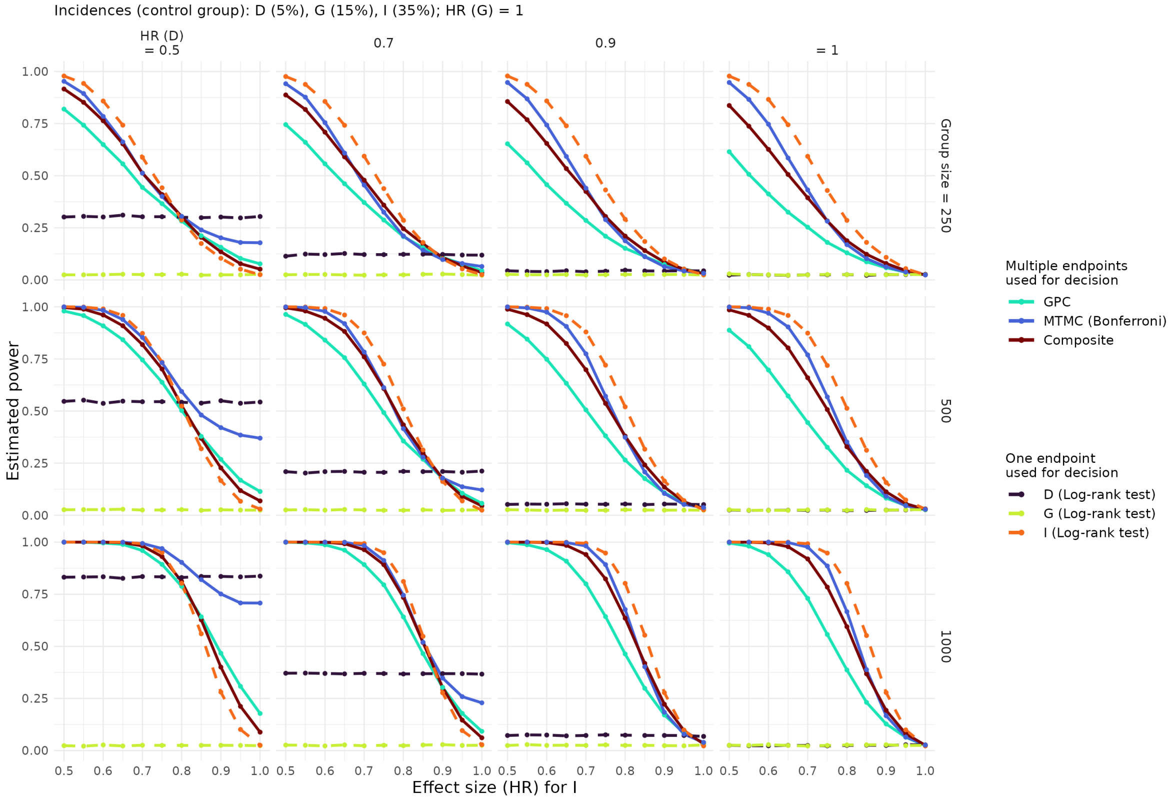

Supplementary Figure S21: Estimated power of the approaches using time-to-event endpoint definitions and one-sided tests in various scenarios including different group sizes. The titles list the parameters not explicitly shown in the plots. Incidences in the title mean the expected proportion of observed deaths (D), graft loss (G) and infections (I) in the control group. The x-axis depicts the hazard ratio (HR) of infections (0.5, large difference, to 1, i.e., equal hazards for infection in both groups). On the y-axis the estimated power is plotted. Hazard ratios of death vary by column and group sizes by row. Solid lines identify procedures taking all endpoints into account; Bonferroni correction (blue), Composite endpoint (darkred), GPC (turquoise). Dashed lines indicate that tests are performed on one single endpoint without multiplicity correction; one-sided log-rank tests of graft loss (light green), infections (orange), and death (black).

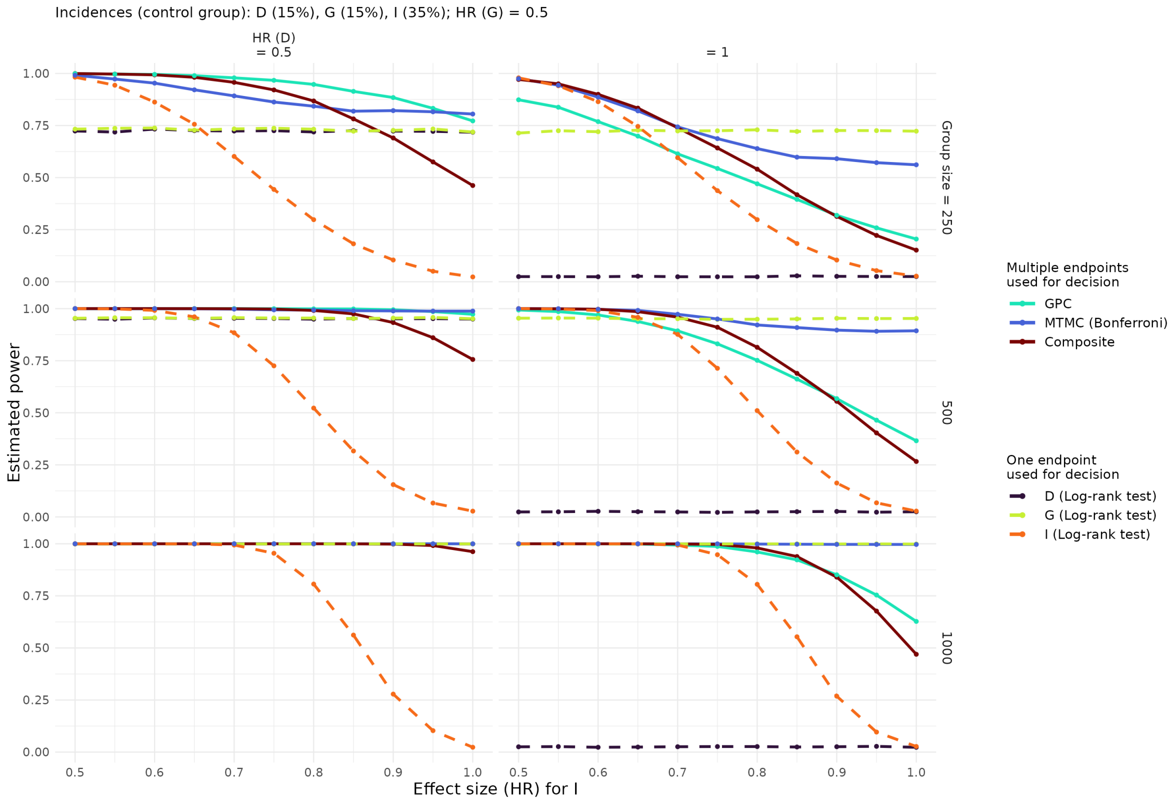

Supplementary Figure S22: Estimated power of the approaches using time-to-event endpoint definitions and one-sided tests in various scenarios including different group sizes. The titles list the parameters not explicitly shown in the plots. Incidences in the title mean the expected proportion of observed deaths (D), graft loss (G) and infections (I) in the control group. The x-axis depicts the hazard ratio (HR) of infections (0.5, large difference, to 1, i.e., equal hazards for infection in both groups). On the y-axis the estimated power is plotted. Hazard ratios of death vary by column (left column 0.5, right column 1) and group sizes by row (250, 500 and 1000 participants per group). Solid lines identify procedures taking all endpoints into account; Bonferroni correction (blue), Composite endpoint (darkred), GPC (turquoise). Dashed lines indicate that tests are performed on one single endpoint without multiplicity correction; one-sided log-rank tests of graft loss (light green), infections (orange), and death (black).

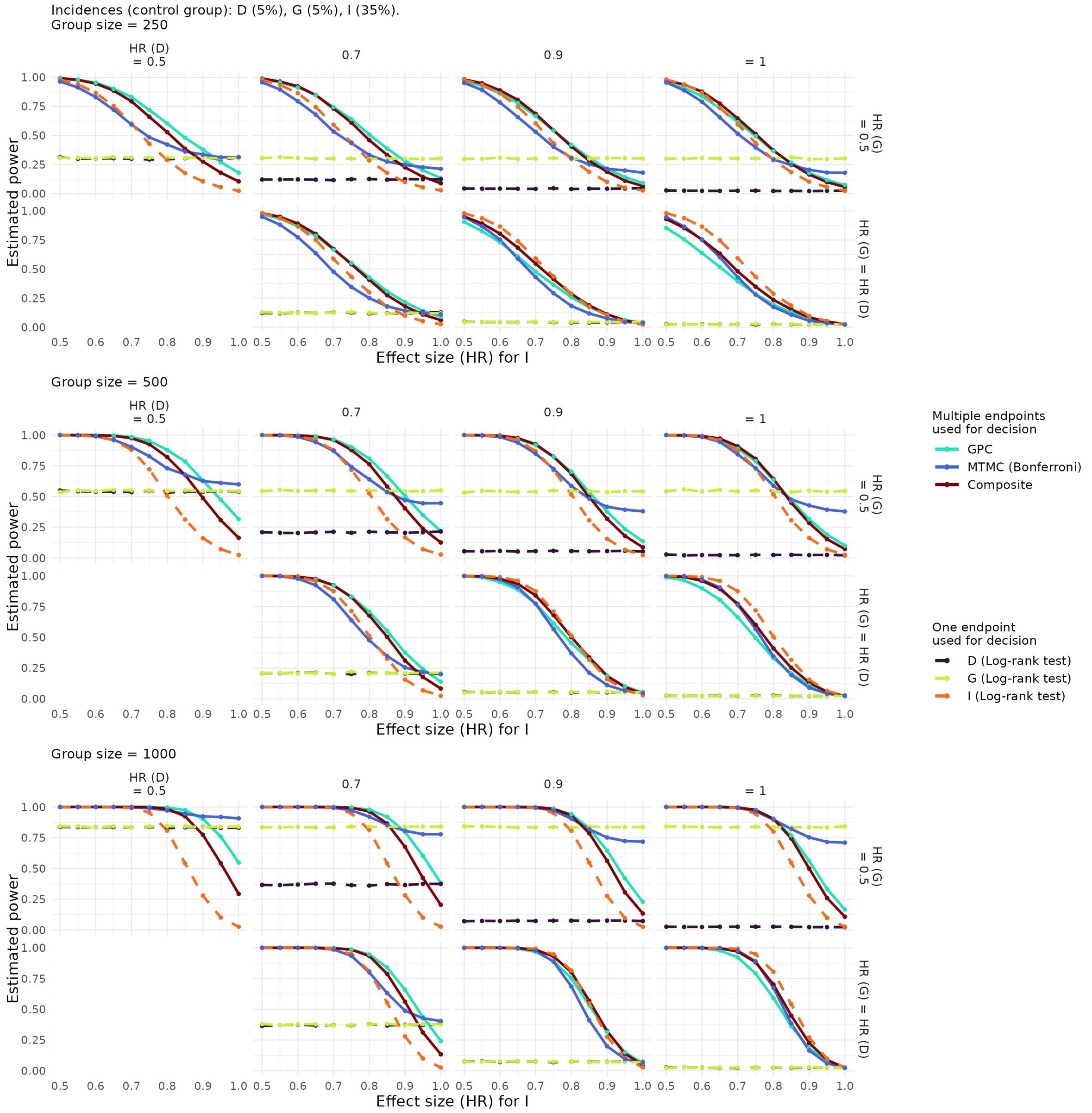

Supplementary Figure S23: Estimated power of the approaches using time-to-event endpoint definitions and one-sided tests in various scenarios including different group sizes. The first title list the parameters not explicitly shown in the plots but constant in all shown scenarios. Incidences in the title mean the expected proportion of observed deaths (D), graft loss (G) and infections (I) in the control group. Three plot grids are shown for Group sizes of 250 (top grid), 500 (middle grid) and 1000 (bottom grid) participants per group. The x-axis depicts the hazard ratio (HR) of infections (0.5, large difference, to 1, i.e., equal hazards for infection in both groups). On the y-axis the estimated power is plotted. Hazard ratios of death vary by column (again from 0.5, a strong treatment effect on deaths, to 1, treatment does not affect the event rate of death). The HR of graft loss varies by row; in the first row of plot grids, the HR of graft loss is 0.5. In the second row of plots, the HR of graft loss is equal to the HR of death in each plot (the first plot is empty, as the HRs of 0.5 are shwon in the first plot of the first row). Solid lines identify procedures taking all endpoints into account; Bonferroni correction (blue), Composite endpoint (darkred), GPC (turquoise). Dashed lines indicate that tests are performed on one single endpoint without multiplicity correction; one-sided log-rank tests of graft loss (light green), infections (orange), and death (black). Note: lower left panels are blank because it is identical to the respective plots above.

E.2 Plots of all scenarios evaluated (two-sided tests)

The following section provides results of all scenarios that were evaluated using tests for differences between treatment groups, i.e. using two-sided tests. Results for two-sided tests are only shown for the data generating mechanism including semi-competing risk of death and uncorrelated latent endpoints. The comparisons of strategies on time-to-event endpoint definitions is shown in Supplementary Fig. S24. The same scenarios are found in S13 where no results for Gray's test are shown. A comparison of one- and two-sided tests is provided in Supplementary Fig. S11. Contrasting weighted and unweighted Bonferroni multiplicity correction in all investigated scenarios using two-sided tests is shown in Supplementary Fig. S25. In these scenarios, the group size is always fixed to 130 participants per group. The results of simulations with larger group sizes using two-sided tests are shown in Supplementary Fig. S26-S29. More facets for HRs of death in between 0.5 and 1 would be available in Supplementary Fig. S27, i.e., more columns in the plot grids would be available. To provide an overview, only a selection of those is displayed.

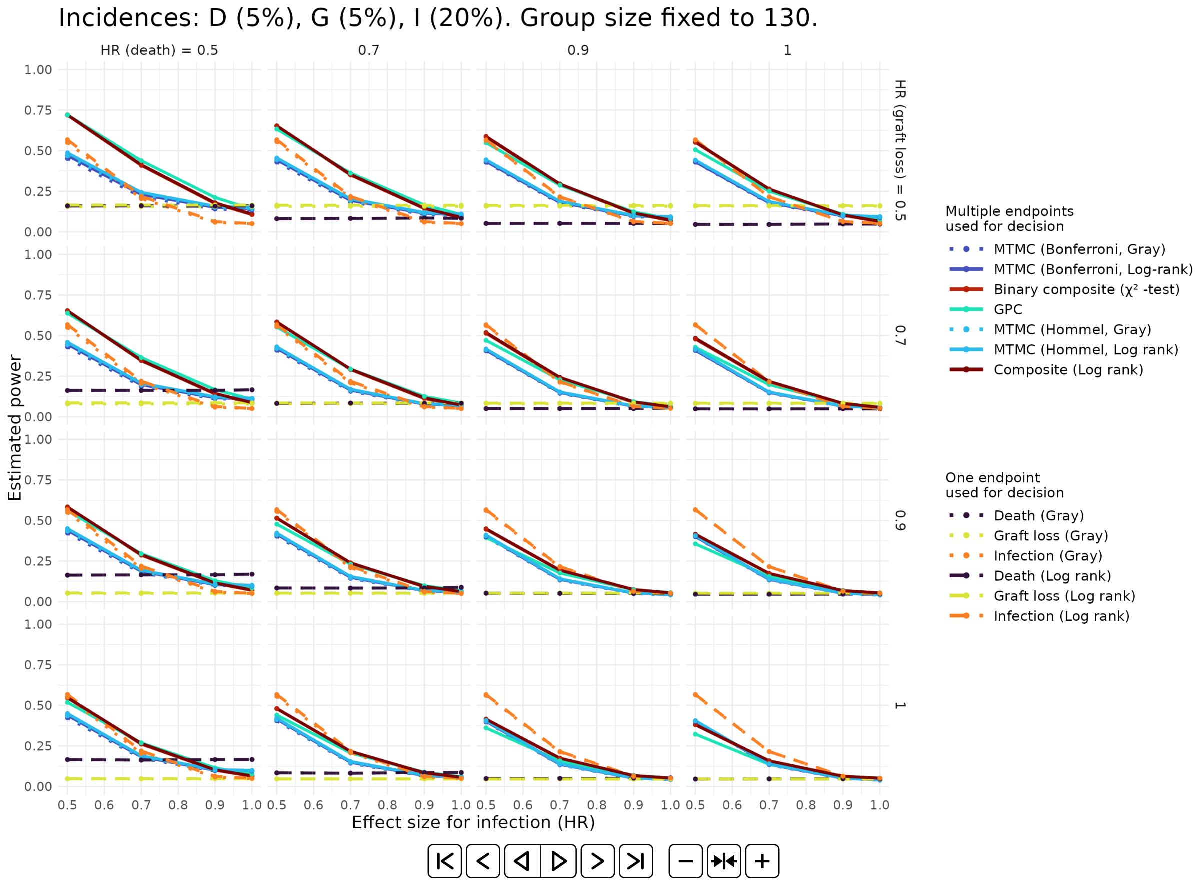

Supplementary Figure S24: ANIMATION VARYING INCIDENCES (values shown in the figure header)Estimated power of the approaches using time-to-event endpoint definitions and two-sided tests in various scenarios. The titles indicate the parameters not explicitly shown in the plots. These are the expected proportions of deaths (D), graft loss (G) and infections (I) in the control group. The group size is fixed to 130 participants. The x-axis of plots depicts the HR of infections (0.5, large difference, to 1, i.e., equal hazards for infection in both groups). On the y-axis the estimated power is plotted. The facet labels of each column give the hazard ratio (HR) of death, the rows the HR of graft loss. The HR of death increases within a row from left to right taking values 0.5, 0.7, 0.9, and 1 and likewise for graft loss in columns. Solid lines identify procedures taking all endpoints into account; Bonferroni correction (blue), Composite endpoint (darkred), GPC (turquoise). Dashed lines indicate that tests are performed on one single endpoint without multiplicity correction; log-rank tests for differences of hazards of graft loss (light green), infections (orange), and death (black). Dotted lines indicate that Gray's test was used instead of log-rank tests.

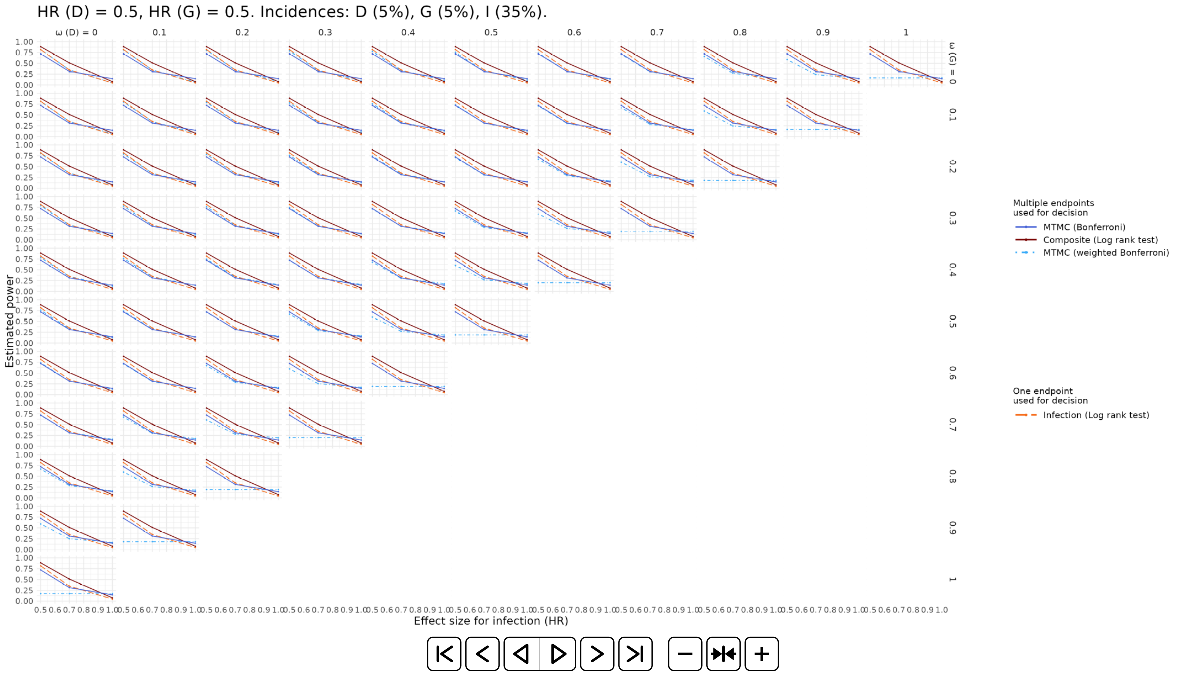

Supplementary Figure S25: ANIMATION VARYING EFFECT SIZES OF DEATH AND GRAFT LOSS (values shown in the figure header)Comparison of estimated power of a weighted Bonferroni procedure and aforementioned approaches in various scenarios. Time-to-event endpoint definitions are again used and differences between groups tested by log-rank tests. The expected proportion of deaths (D), graft losses (G) and infections (I) in the control group is fixed to 5%, 5% and 35%, respectively, in all shown plots. Hazard ratios (HR) of D and G vary in each plot and are given in the title of each figure. The x-axis of plots depicts the HR of infections (0.5, a strong treatment effect, to 1, i.e., no effect). On the y-axis the estimated power is plotted. The weights, ω, applied to adjust the tests p-values varies and is given in the column (weight of the test of D) and row (weight of the test of G) facet labels . Solid lines identify procedures taking all endpoints into account; Multiple testing and multiplicity correction (MTMC) using log-rank tests and Bonferroni correction (blue), Composite endpoint (darkred). Dashed lines indicate log-rank tests of infections (orange). Dashed-dotted lines are the weighted version of the Bonferroni correction (blue). Note that as the weights must sum to one, the weight of the tests of I follow from the other two weights. For the same reason, combinations of weights in the right-lower triangle do not exist and the plots remain empty.

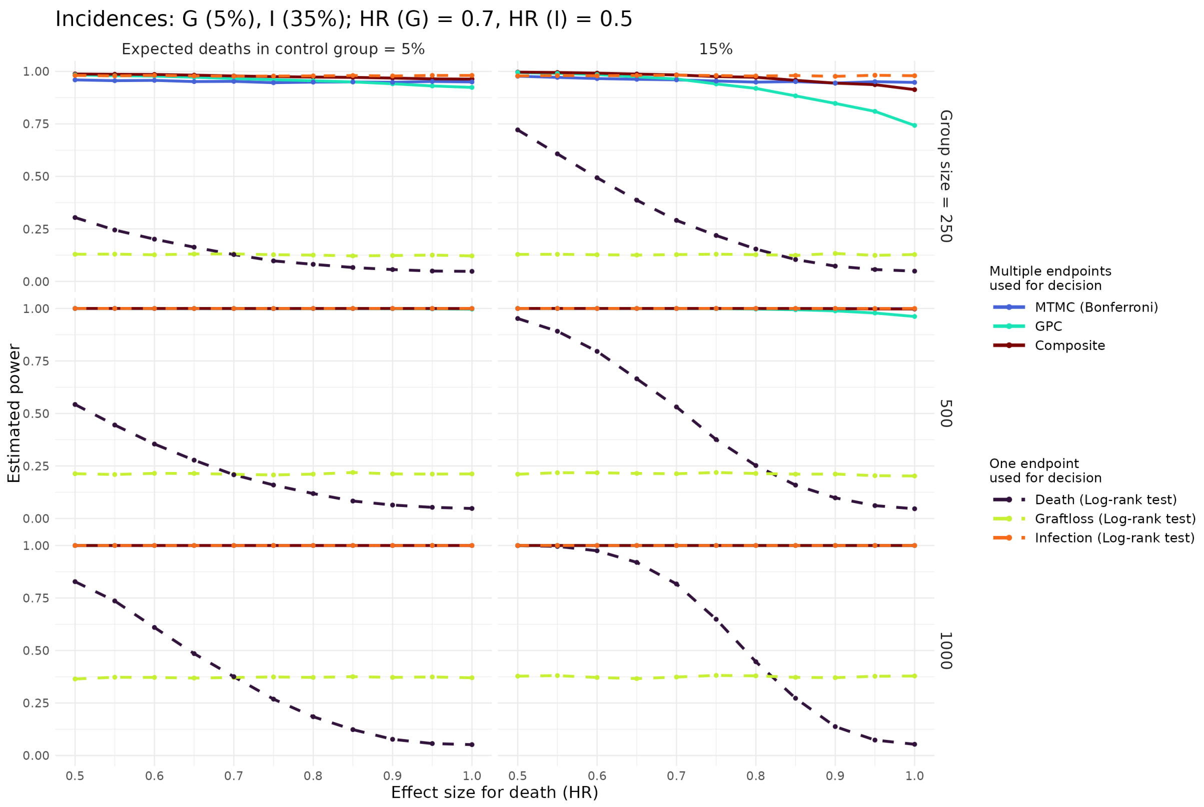

Supplementary Figure S26: Estimated power of the approaches using time-to-event endpoint definitions and two-sided tests in various scenarios including different group sizes. The titles list the parameters not explicitly shown in the plots. Incidences in the title mean the expected proportion of observed deaths (D), graft loss (G) and infections (I) in the control group. The x-axis depicts the hazard ratio (HR) of death (0.5, large difference, to 1, i.e., equal hazards for infection in both groups). On the y-axis the estimated power is plotted. The left column of plots are scenarios with incidences of death of 5%, in the right column the incidence of death is 15%. The group sizes increase by rows from 250 to 500 and 1000 participants per group. Solid lines identify procedures taking all endpoints into account; Bonferroni correction (blue), Composite endpoint (darkred), GPC (turquoise). Dashed lines indicate that tests are performed on one single endpoint without multiplicity correction; log-rank tests for differences of hazards of graft loss (light green), infections (orange), and death (black).

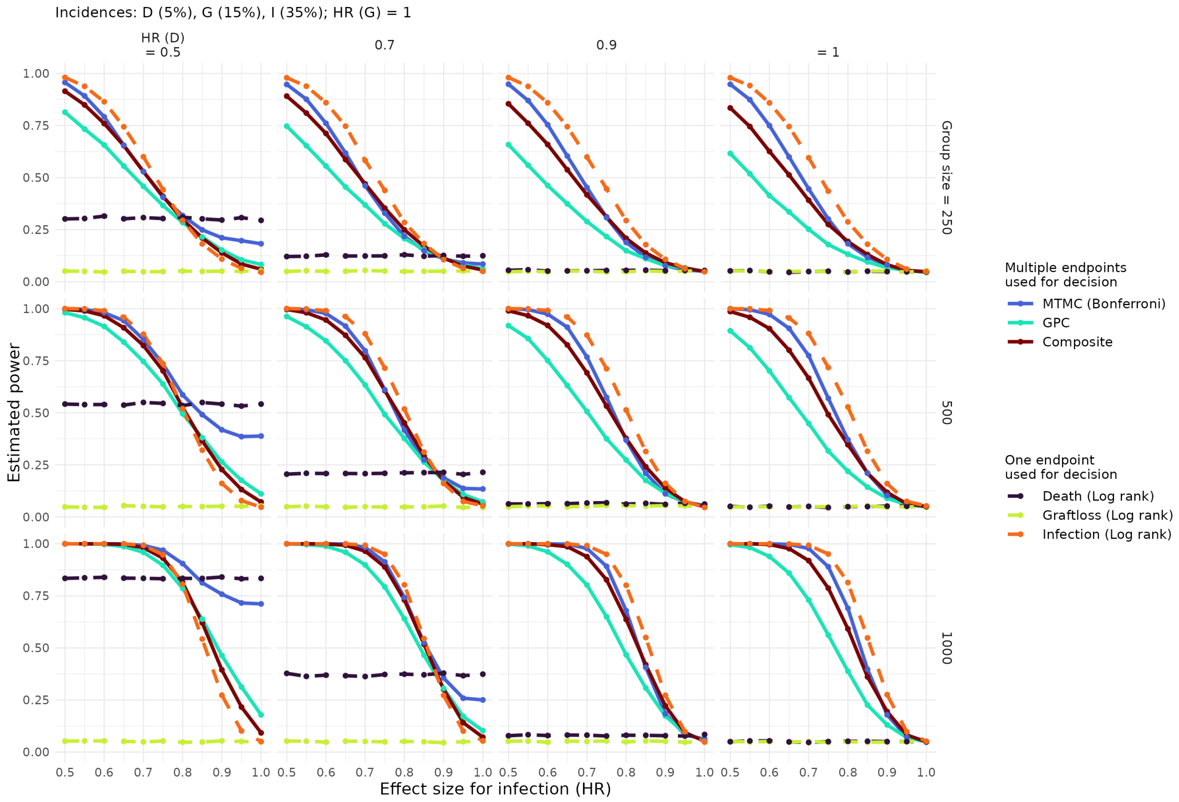

Supplementary Figure S27: Estimated power of the approaches using time-to-event endpoint definitions and two-sided tests in various scenarios including different group sizes. The titles list the parameters not explicitly shown in the plots. Incidences in the title mean the expected proportion of observed deaths (D), graft loss (G) and infections (I) in the control group. The x-axis depicts the hazard ratio (HR) of infections (0.5, large difference, to 1, i.e., equal hazards for infection in both groups). On the y-axis the estimated power is plotted. Hazard ratios of death vary by column and group sizes by row. Solid lines identify procedures taking all endpoints into account; Bonferroni correction (blue), Composite endpoint (darkred), GPC (turquoise). Dashed lines indicate that tests are performed on one single endpoint without multiplicity correction; log-rank tests for differences of hazards of graft loss (light green), infections (orange), and death (black).

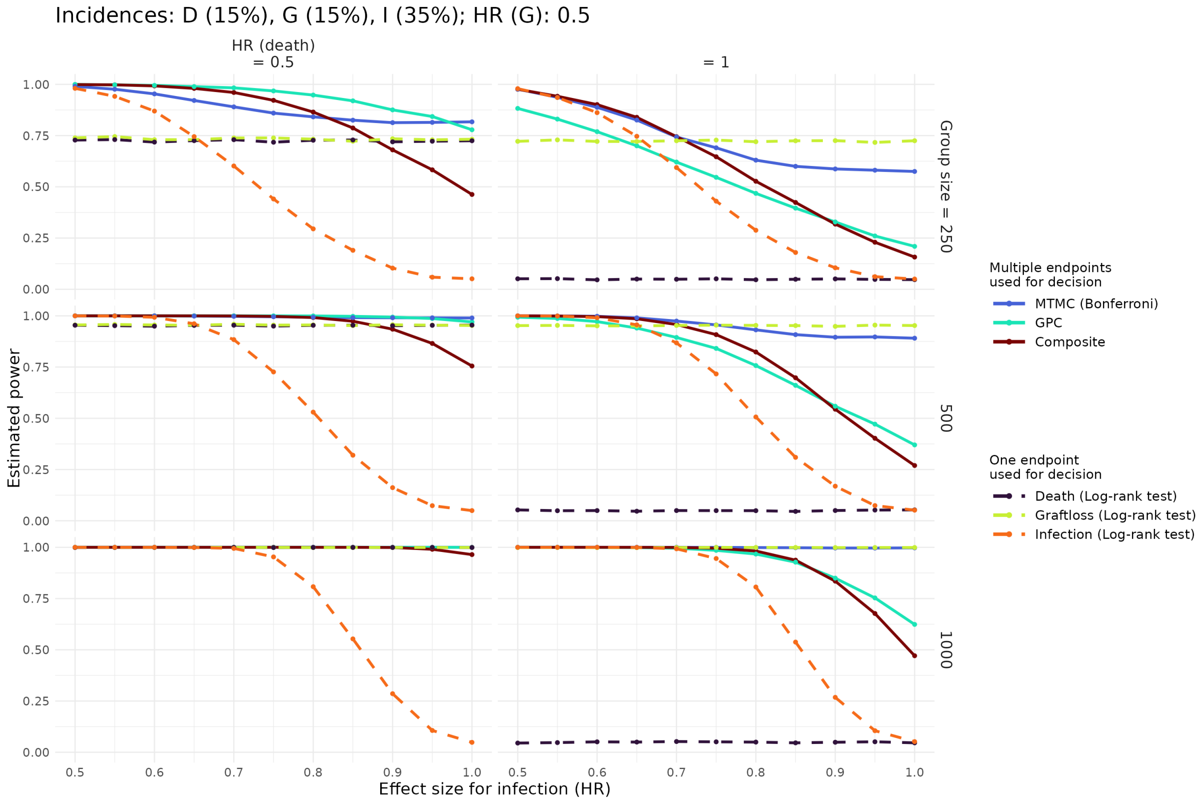

Supplementary Figure S28: Estimated power of the approaches using time-to-event endpoint definitions and two-sided tests in various scenarios including different group sizes. The titles list the parameters not explicitly shown in the plots. Incidences in the title mean the expected proportion of observed deaths (D), graft loss (G) and infections (I) in the control group. The x-axis depicts the hazard ratio (HR) of infections (0.5, large difference, to 1, i.e., equal hazards for infection in both groups). On the y-axis the estimated power is plotted. Hazard ratios of death vary by column (left column 0.5, right column 1) and group sizes by row (250, 500 and 1000 participants per group). Solid lines identify procedures taking all endpoints into account; Bonferroni correction (blue), Composite endpoint (darkred), GPC (turquoise). Dashed lines indicate that tests are performed on one single endpoint without multiplicity correction; log-rank tests for differences of hazards of graft loss (light green), infections (orange), and death (black).

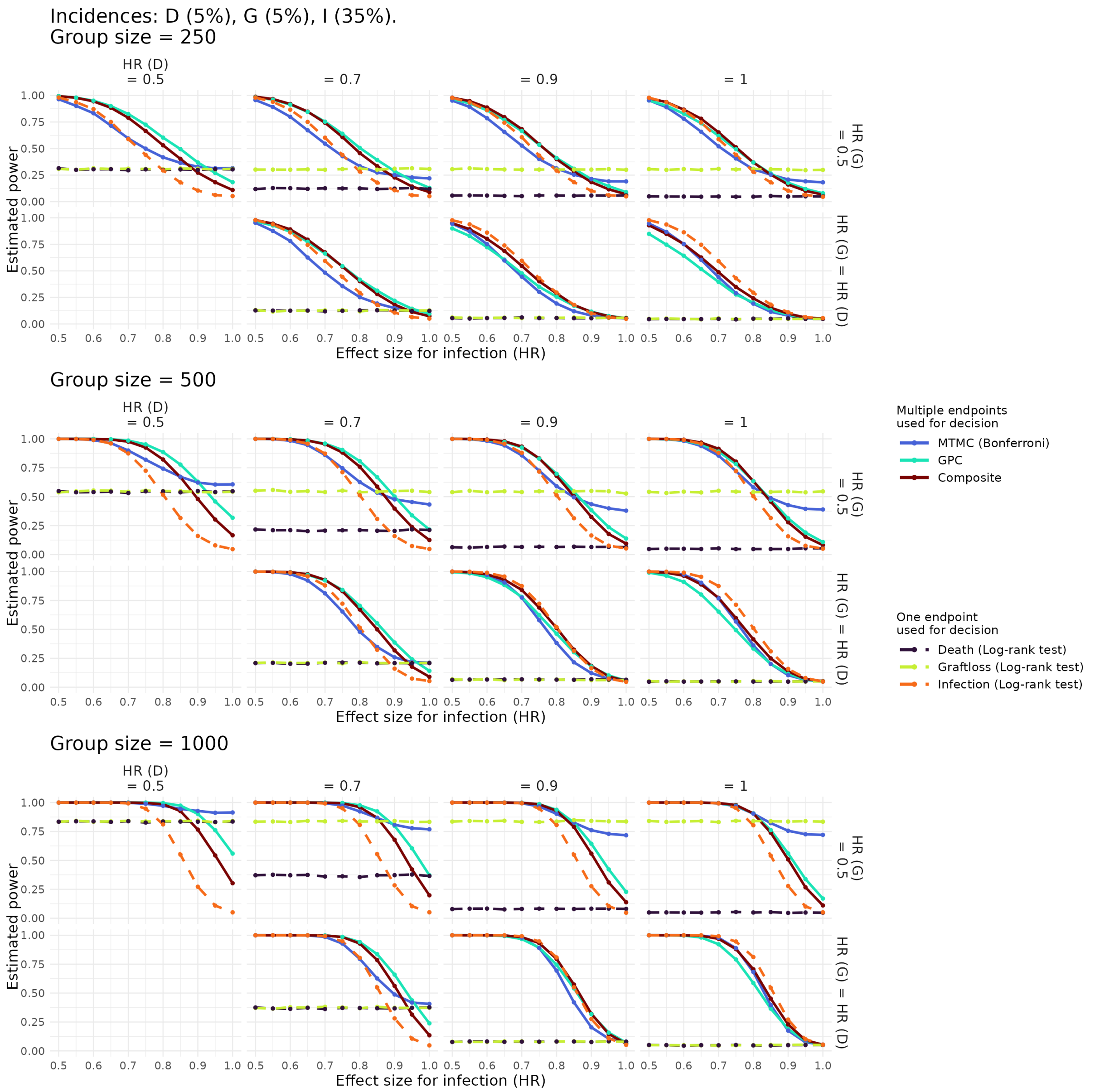

Supplementary Figure S29: Estimated power of the approaches using time-to-event endpoint definitions and two-sided tests in various scenarios including different group sizes. The first title list the parameters not explicitly shown in the plots but constant in all shown scenarios. Incidences in the title mean the expected proportion of observed deaths (D), graft loss (G) and infections (I) in the control group. Three plot grids are shown for Group sizes of 250 (top grid), 500 (middle grid) and 1000 (bottom grid) participants per group. The x-axis depicts the hazard ratio (HR) of infections (0.5, large difference, to 1, i.e., equal hazards for infection in both groups). On the y-axis the estimated power is plotted. Hazard ratios of death vary by column (again from 0.5, a strong treatment effect on deaths, to 1, treatment does not affect the event rate of death). The HR of graft loss varies by row; in the first row of plot grids, the HR of graft loss is 0.5. In the second row of plots, the HR of graft loss is equal to the HR of death in each plot (the first plot is empty, as the HRs of 0.5 are shown in the first plot of the first row). Solid lines identify procedures taking all endpoints into account; Bonferroni correction (blue), Composite endpoint (darkred), GPC (turquoise). Dashed lines indicate that tests are performed on one single endpoint without multiplicity correction; log-rank tests for differences of hazards of graft loss (light green), infections (orange), and death (black). Note: lower left panels are blank because it is identical to the respective plots above.

F List of abbreviations used

Supplementary Table S7: List of abbreviations used in the main manuscript and Supplementary Material

Abbreviation	Full term
BIN	Binary
BIN CE	Binary composite endpoint
CE	Composite endpoint
CIF	Cumulative incidence function
D	Denotes the endpoint death
EMA	European Medicines Agency
FDA	Food and Drug Administration
FWER	Family-wise error rate
G	Denotes the endpoint graft loss
GPC	Generalized pairwise comparisons
HR	Hazard ratio
I	Denotes the endpoint infection
ICH	International Council for Harmonisation
KM	Kaplan–Meier
MACE	Major adverse cardiovascular events
MCSE	Monte Carlo standard error
MTMC	Multiple testing and multiplicity correction
NTB	Net treatment benefit
OS	Overall survival
PH	Proportional hazards
PRO	Patient-reported outcome
R	Denotes the endpoint rejection
RCT	Randomized controlled trial
RMST	Restricted mean survival time
TTE	Time-to-event
TTE CE	Time-to-event composite endpoint

References

[1] Norbert Benda, Michael Branson, Willi Maurer, and Tim Friede. Aspects of modernizing drug development using clinical scenario planning and evaluation. *Drug information journal: DIJ/Drug Information Association*, 44:299–315, 2010.

[2] Jack Cuzick. The efficiency of the proportions test and the logrank test for censored survival data. *Biometrics*, pages 1033–1039, 1982.

[3] Tim Friede, Richard Nicholas, Nigel Stallard, Susan Todd, Nicholas Parsons, Elsa Valdés-Márquez, and Jeremy Chataway. Refinement of the clinical scenario evaluation framework for assessment of competing development strategies with an application to multiple sclerosis. *Drug information journal: DIJ/Drug Information Association*, 44:713–718, 2010.

[4] Mitchell H Gail. Applicability of sample size calculations based on a comparison of proportions for use with the logrank test. *Controlled clinical trials*, 6(2):112–119, 1985.

[5] Bob Gray. *cmprsk: Subdistribution Analysis of Competing Risks*, 2024. R package version 2.2-12.

[6] Marius Hofert, Ivan Kojadinovic, Martin Maechler, and Jun Yan. *copula: Multivariate Dependence with Copulas*, 2025. R package version 1.1-6.

[7] Tim P Morris, Ian R White, and Michael J Crowther. Using simulation studies to evaluate statistical methods. *Statistics in medicine*, 38(11):2074–2102, 2019.

[8] Brice Ozenne and Julien Peron. *BuyseTest: Implementation of the Generalized PairwiseComparisons*, 2025. R package version 3.2.0.

[9] R Core Team. *R: A Language and Environment for Statistical Computing.* R Foundation for Statistical Computing, Vienna, Austria, 2025.

[10] The European Agency for the Evaluation of Medicinal Products (EMEA). Points to consider on multiplicity issues in clinical trials. Technical Report CPMP/EWP/908/99, European Medicines Agency, 2002. Online; accessed 20-January-2025.

[11] Terry M Therneau. *A Package for Survival Analysis in R*, 2024. R package version 3.8-3.

[12] U.S. Food and Drug Administration. Multiple endpoints in clinical trials: guidance for industry. Technical Report FDA-2016-D-4460, U.S. Department of Health and Human Services, Food and Drug Administration,Center for Biologics Evaluation and Research (CBER), Center for Drug Evaluation and Research (CDER), 2022. Online; accessed 29-October-2024.
